# The associations between plasma soluble Trem1 and neurological diseases: a Mendelian randomization study

**DOI:** 10.1186/s12974-022-02582-z

**Published:** 2022-09-06

**Authors:** Xiaolei Shi, Tao Wei, Yachun Hu, Meng Wang, Yi Tang

**Affiliations:** 1 Geriatric Neuroscience Center, The Affiliated Brain Hospital of Guangzhou Medical University, Guangzhou, China; 2 Guangdong Engineering Technology Research Center for Translational Medicine of Mental Disorders, Guangzhou, China; 3 Innovation Center for Neurological Disorders, Department of Neurology, National Center for Neurological Disorders, Xuanwu Hospital, Capital Medical University, Beijing, China; 4 Department of Neurology, The Affiliated Brain Hospital of Guangzhou Medical University, Guangzhou, China; 5 Neurodegenerative Laboratory of Ministry of Education of the Peoples Republic of China, Beijing, China

**Keywords:** sTrem1, Neurological diseases, Alzheimer’s disease, Epilepsy, Mendelian randomization

## Abstract

**Background:**

Triggering receptor expressed on myeloid cell 1 (Trem1) is an important regulator of cellular inflammatory responses. Neuroinflammation is a common thread across various neurological diseases. Soluble Trem1 (sTrem1) in plasma is associated with the development of central nervous system disorders. However, the extent of any causative effects of plasma sTrem1 on the risk of these disorders is still unclear.

**Method:**

Genetic variants for plasma sTrem1 levels were selected as instrumental variables. Summary-level statistics of neurological disorders, including Alzheimer’s disease (AD), Parkinson’s disease (PD), amyotrophic lateral sclerosis (ALS), multiple sclerosis (MS), epilepsy, cerebrovascular diseases, and migraine were collected from genome-wide association studies (GWASs). Whether plasma sTrem1 was causally associated with neurological disorders was assessed using a two-sample Mendelian randomization (MR) analysis, with false discovery rate (FDR)-adjusted methods applied.

**Results:**

We inferred suggestive association of higher plasma sTrem1 with the risk of AD (odds ratio [OR] per one standard deviation [SD] increase = 1.064, 95% CI 1.012–1.119, *P* = 0.014, *P*_*FDR*_ = 0.056). Moreover, there was significant association between plasma sTrem1 level and the risk of epilepsy (OR per one SD increase = 1.044, 95% CI 1.016–1.072, *P* = 0.002, *P*_*FDR*_ = 0.032), with a modest statistical power of 41%. Null associations were found for plasma sTrem1 with other neurological diseases and their subtypes.

**Conclusions:**

Taken together, this study indicates suggestive association between plasma sTrem1 and AD. Moreover, higher plasma sTrem1 was associated with the increased risk of epilepsy. The findings support the hypothesis that sTrem1 may be a vital element on the causal pathway to AD and epilepsy.

**Supplementary Information:**

The online version contains supplementary material available at 10.1186/s12974-022-02582-z.

## Introduction

Neuroinflammation is a prominent feature of central nervous system (CNS) diseases [1, 2]. For instance, Alzheimer’s disease (AD), as a typical neurodegenerative disorder, represents progressive proteinopathy, along with the infiltration of immune cells in brains [1]. In multiple sclerosis (MS), the invasion of autoreactive inflammatory cells facilitates the damage of myelin and oligodendrocytes [3]. The common inflammatory thread connects infectious, neurodegenerative, and psychiatric conditions, rendering itself to interfere with the intrinsic pathological processes and further promote progression of CNS disorders [2]. Therefore, interest is increasing in the development of anti-inflammatory therapies targeting specific factors to alleviate the pathological changes across neurological disease continuum [4]. Although agents have been studied in the past decades, the lack of precise and effective target is still a major challenge.

Triggering receptor expressed on myeloid cell 1 (Trem1) belongs to the immunoglobulin Trem family, which is expressed on myeloid cells to modulate cell activation and differentiation [5]. Since Trem1 was discovered by Bouchon et al. [6] in 2000, it has been regarded as a promising marker for its importance in inflammatory responses [7, 8]. Human Trem1 consists of an ectodomain, a transmembrane domain and a short cytoplasmic structure. Once activated, it couples with TYRO protein tyrosine kinase-binding protein (TYROBP), contributing to the activation of immune cells and neuronal death in pathological scenarios [9–11]. Studies have also shown that a soluble form of Trem1 (sTrem1) can be measured in plasma, cerebrospinal fluid (CSF), and bronchoalveolar lavage fluid from patients suffering from inflammatory conditions [12–15]. The molecule in fluid is used to facilitate early diagnosis and determination of disease prognosis. Higher level of plasma sTrem1 is considered to be involved in systemic sepsis, acute myocardial infarction, and subarachnoid hemorrhage [16–18]. Moreover, increasing plasma sTrem1 is likely to be associated with AD severity [12]. Despite the recently observed findings between sTrem1 and neurological disorders, evidence is too inconclusive to establish reliable causal associations. The available findings tend to be susceptible to confounding factors and possible reverse causation. Therefore, it is of great importance to understand the causal effects of sTrem1 on the risk of neurological disorders.

Mendelian randomization (MR) provides a genetic epidemiological approach to explore the causality between clinical traits and disease phenotypes using genetic variants as instrumental variables [19, 20]. MR shows superiority in the control of confounding that can cause bias in observational studies, by leveraging the random allocation of genetic alleles to offspring before birth [19, 20]. It is believed that MR can help expose the unbiased causal effects of modifiable traits on the outcomes of interests among certain populations. Therefore, the current study investigated the genetic validity of plasma sTrem1 levels in the risk of neurological disorders, including AD, PD, amyotrophic lateral sclerosis (ALS), MS, epilepsy, cerebrovascular diseases, and migraine, by using a two-sample MR method.

## Materials and methods

### Study design

**Fig. 1:**
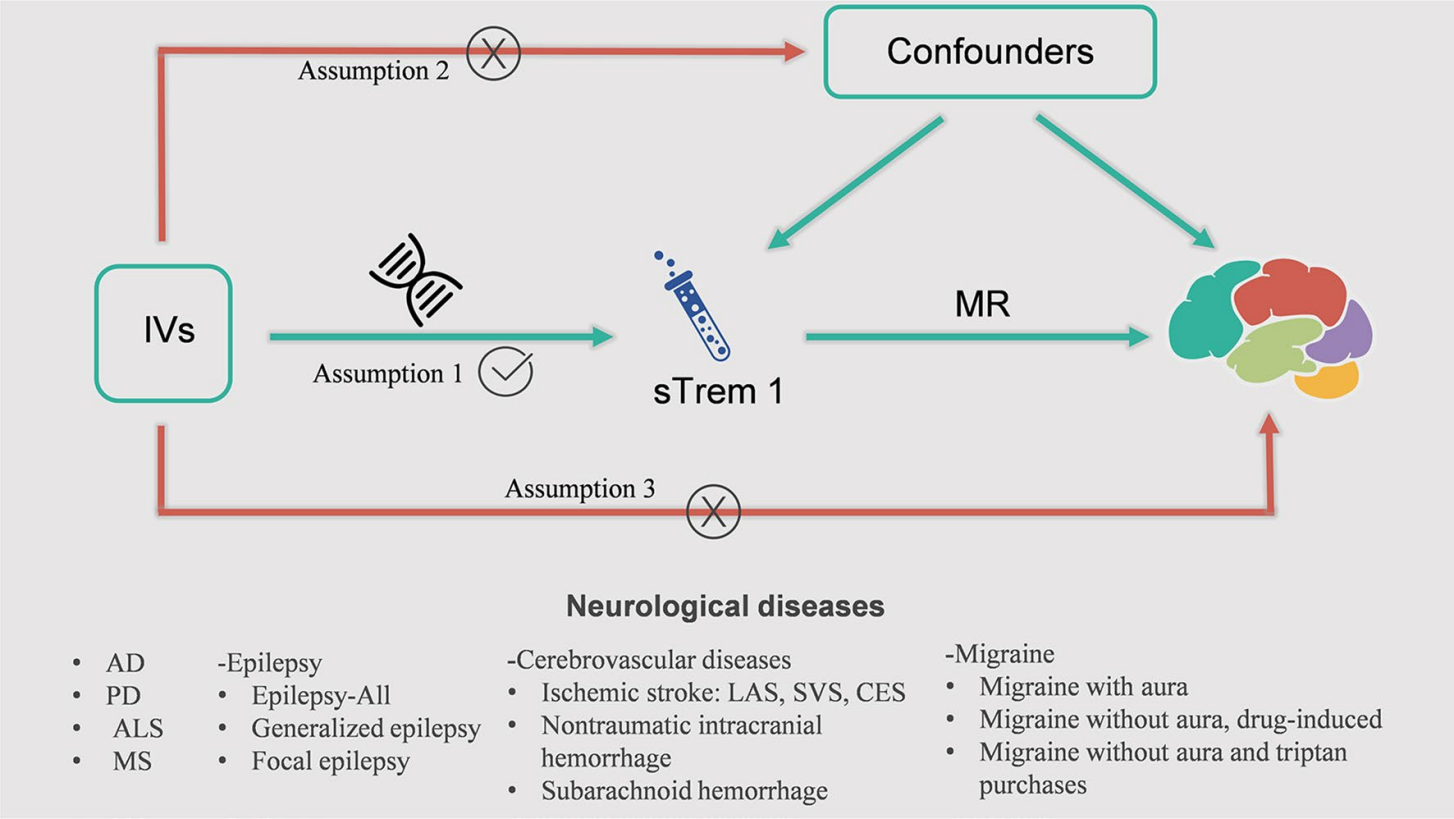
The overall design of Mendelian randomization analysis in the present study. Assumption 1, the genetic variants are supposed to be strongly associated with the risk of interest; Assumption 2, the genetic variants should not be associated with any confounding factors; and Assumption 3, the genetic variants should affect the risk of the outcome only mediated by the exposures. AD, Alzheimer’ s Disease; ALS, amyotrophic lateral sclerosis; CES, cardioembolic stroke; IV, instrumental variable; LAS, large-artery atherosclerosis stroke; MR, Mendelian randomization; MS, multiple sclerosis; PD, Parkinson’s Disease; sTrem1, soluble triggering receptor expressed on myeloid cell 1; SVS, small-vessel stroke

In the present study, we performed a univariable two-sample MR analysis to estimate the causal associations between plasma sTrem1 levels and neurological diseases (i.e., AD, PD, ALS, MS, epilepsy, cerebrovascular diseases, migraine along with their subtypes) (Table 1). The whole design of our MR framework is presented in Fig. 1. To explore the causal effects of exposure on outcome, MR analysis needs to satisfy three assumptions: (1) the genetic variants are supposed to be strongly associated with the risk of interest; (2) the genetic variants should not be associated with any confounding factors; and (3) the genetic variants should affect the risk of the outcome only mediated by the exposures [21]. In addition, the reverse-MR analysis was conducted to avoid the potential effects of reverse causality. This study used publicly available genome-wide association studies (GWASs) summary data and thus no separate ethical approval was required for this study.

**Table 1:** Detailed information of the studies and datasets used for Mendelian randomization analysis

Phenotype	Sample size (cases/controls)	Population	Consortium	Year	Journal	References
Plasma sTrem1	3301	European	–	2018	Nature	Sun et al. [22]
Neurodegenerative disease						
Alzheimer’s disease	21,982/41,944	European	IGAP	2019	Nat Genet	Kunkle et al. [23]
Parkinson’s disease	33,674/449,056	European	IPDGC	2019	Lancet Neurol	Nalls et al. [24]
Amyotrophic lateral sclerosis	20,806/59,804	European	AVS	2018	Neuron	Nicolas et al. [25]
Multiple sclerosis	47,429/68,374	European	IMSGC	2019	Science	Patsopoulos et al. [26]
Epilepsy						
Epilepsy	15,212/29,677	Mixed	ILAE	2018	Nat Commun	Abou-Khalil et al. [27]
Generalized epilepsy	3769/29677	Mixed	ILAE	2018	Nat Commun	Abou-Khalil et al. [27]
Focal epilepsy	9671/29677	Mixed	ILAE	2018	Nat Commun	Abou-Khalil et al. [27]
Cerebrovascular diseases						
Ischemic stroke	34,217/406111	European	MEGASTROKE	2018	Nat Genet	Malik et al. [28]
Large-artery atherosclerosis	4373/406,111	European	MEGASTROKE	2018	Nat Genet	Malik et al. [28]
Small-vessel	5386/192662	European	MEGASTROKE	2018	Nat Genet	Malik et al. [28]
Cardioembolic	7193/406111	European	MEGASTROKE	2018	Nat Genet	Malik et al. [28]
Nontraumatic intracranial hemorrhage	2794/203068	European	FinnGen	2021	–	–
Subarachnoid hemorrhage	1338/201230	European	FinnGen	2021	–	–
Migraine						
Migraine with aura	3541/176107	European	FinnGen	2021	–	–
Migraine without aura, drug-induced	180/218612	European	FinnGen	2021	–	–
Migraine without aura and triptan purchases	3215/164098	European	FinnGen	2021	–	–

AVS: ALS Variant Server; IGAP: International Genomics of Alzheimer’s Project; ILAE: International League Against Epilepsy; IMSGC: International Multiple Sclerosis Genetics Consortium; IPDGC: International Parkinson’s Disease Genomics Consortium; sTrem1: soluble Triggering receptor expressed on myeloid cell 1

### Data sources

#### GWAS of plasma sTrem1

In this MR analysis, genetic instruments for plasma sTrem1 were obtained from a large-scale GWAS, including 3301 individuals from the INTERVAL study [22]. The INTERVAL study is a prospective cohort study that recruited approximately 50,000 blood donors aged ≥ 18 years. The participants included in the dataset above were mainly of European ancestry. Plasma sTrem1 levels were measured using a multiplexed, aptamer-based approach (SOMAscan assay) and the reliability of protein levels was further verified by a series of subsequent experiments.

#### GWAS of neurological diseases

The analysis used genetic variants from the International Genomics of Alzheimer's Project (IGAP) (including 21,982 AD cases and 41,944 controls) [23], the International Parkinson's Disease Genomics Consortium (IPDGC) (including 33,674 PD cases and 449,056 controls) [24], the ALS Variant Server (including 20,806 ALS cases and 59,804 controls) [25], and the International Multiple Sclerosis Genetics Consortium (IMSGC) (including 47,429 MS cases and 68,374 controls) [26]. The individuals included in the datasets were also of European ancestry. Principal covariates, such as age and sex, were adjusted for in the association tests in all sources.

Summary-level data for epilepsy and subtypes were obtained from the International League Against Epilepsy (ILAE) consortium, which included 15,212 cases for all epilepsy, 3769 cases with generalized epilepsy, 9671 cases with focal epilepsy, and 29,677 controls [27]. Summary statistical data for migraine and subtypes were extracted from the FinnGen consortium, including 179,648 European individuals for migraine with aura (including 3541 cases and 176,107 controls), 218,792 European individuals for migraine without aura, drug-induced (including 180 cases and 218,612 controls), and 167,313 European individuals for migraine without aura and triptan purchases (including 3,215 cases and 164,098 controls) (https://fnngen.gitbook.io/documentation/).

Summary GWAS data for associations with ischemic stroke were obtained from the MEGASTROKE consortium, including 34,217 patients and 406,111 controls of European ancestry. The cases were further divided into three subtypes: large-artery atherosclerotic stroke (*n* = 4373), small-vessel stroke (*n* = 5386), and cardioembolic stroke (*n* = 7193) [28]. Summary-level data were also acquired from the FinnGen consortium to identify the genetic liability of both nontraumatic intracranial hemorrhage (2794 cases and 203,068 controls) and subarachnoid hemorrhage (1338 cases and 201,230 controls).

### Selection of instruments

Only two SNPs (rs3789204 and rs56302558) for sTrem1 were extracted when the threshold of genome-wide significance was set at 5 × 10^–8^. Thus, we relaxed the criteria to 1 × 10^–5^ for instrumental variable selection (Additional file 1: Table S4). In addition, the threshold was set at *P* < 5 × 10^–8^ in the inverse analysis (Additional file 1: Tables S5–S9). Next, we excluded SNPs that were in linkage disequilibrium (*r*^2^ threshold < 0.001 within a 10-Mb window), and we extracted the retained SNPs from the outcome datasets [29]. For the SNPs that were not available in the outcome GWAS dataset, proxy SNPs in LD (*r*^2^ > 0.8) were used to replace them [30]. Furthermore, we calculated the F statistic to ensure the strength of the exposures, and an *F* statistic > 10 was considered robust enough to against weak instrument bias [31]. The R^2^ and F statistic of each SNP were calculated according to the formulas: *R*^2^ = 2 × EAF × (1 − EAF) × β^2^ and *F* statistic = *R*^2^ × (*N* − 2)/(1 − *R*^2^). Moreover, we used the PhenoScanner database (Version 2, http://www.phenoscanner.medschl.cam.ac.uk/) [32] to detect other genome-wide significant (*P* < 5 × 10^–8^) traits associated with the chosen SNPs, which may affect the included neurological diseases. The GWAS Catalog (https://www.ebi.ac.uk/gwas) [33] was also reviewed to screen the SNPs. Then the related SNPs were removed from the respective analysis (Additional file 1: Table S3).

### Statistical analysis

In our main analysis, we applied the inverse-variance weighted (IVW) approach to combine the effect estimates from a single IV to acquire the causal analysis [34]. Alternative methods, including weighted median, MR-Egger, the MR-pleiotropy residual sum and outlier (MR-PRESSO), simple mode, and weighted mode were used as sensitivity analysis approaches. Detailed information about the MR methods mentioned above has been explained previously [35, 36]. Cochran's Q statistic and leave-one-out analysis were performed to evaluate the degree of heterogeneity across each SNP. The MR-Egger intercept test, the MR-PRESSO global test, and visual inspection of the funnel plot were conducted to detect horizontal pleiotropy. The *P* value < 0.05 indicated that the IVW results might be invalid due to horizontal pleiotropy.

MR associative analyses were adjusted for multiple testing by calculating false discovery rate (FDR)-corrected *P* values with the Benjamini–Hochberg method. In addition, unadjusted, raw *P* values were also depicted in the text. A threshold of 0.05 was set to declare significant association for FDR-adjusted *P* value; a raw *P* value less than 0.05, but a *P*_*FDR*_ above the criteria of 0.05 was considered as suggestive evidence for reported association. All statistical analyses were performed with the TwoSampleMR [37] and MR-PRESSO [38] packages in R version 4.1.1. Power calculation was performed using the online power calculator (mRnd) (https://cnsgenomics.com/shiny/mRnd/) [31].

## Results

### Associations of plasma sTrem1 levels with AD

**Fig. 2:**
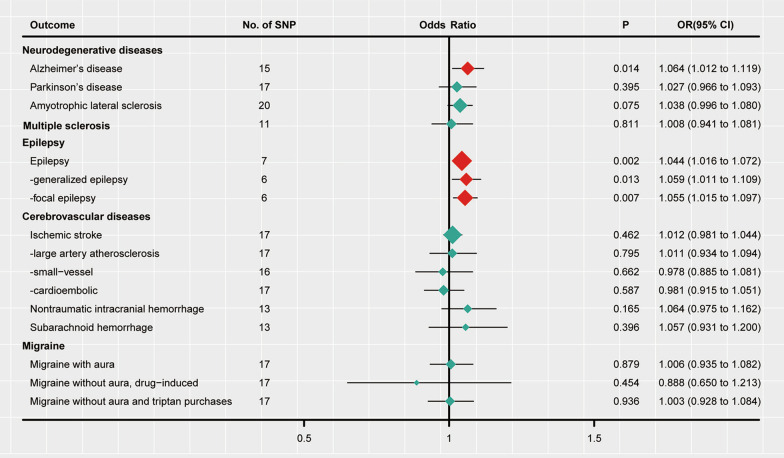
Mendelian randomization analysis estimates of plasma sTrem1 and the risk of neurological diseases. CI: confidence interval; OR: odds ratio; SNP: single-nucleotide polymorphism; sTrem1: soluble triggering receptor expressed on myeloid cell 1. The OR and their 95% CI are scaled to one SD increase in plasma sTrem1 level

**Fig. 3:**
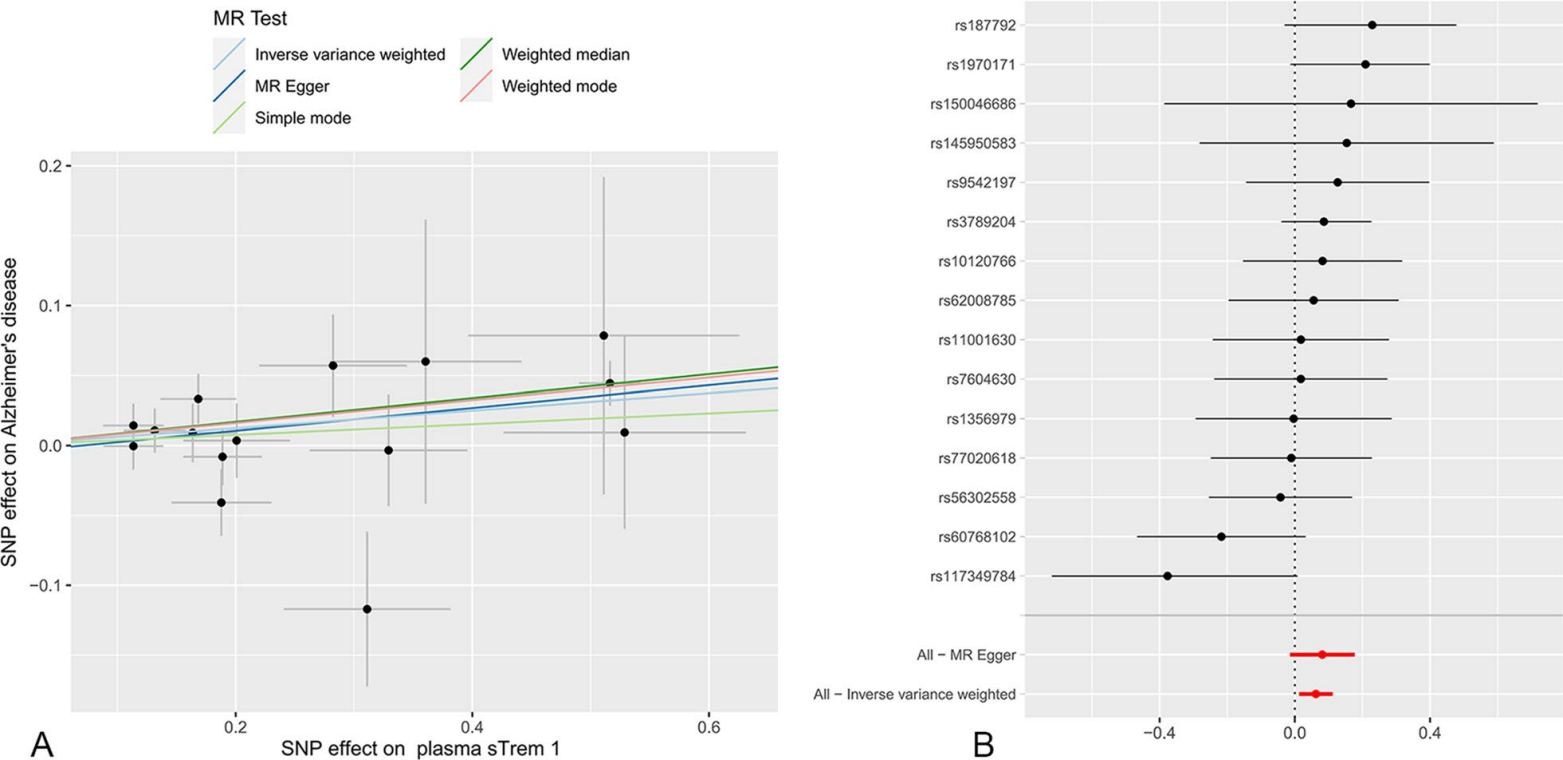
Scatter plot (**A**) and forest plot (**B**) of the causal effect of plasma sTrem1 on AD risk. AD: Alzheimer’ s Disease; sTrem1: soluble triggering receptor expressed on myeloid cell 1

**Fig. 4:**
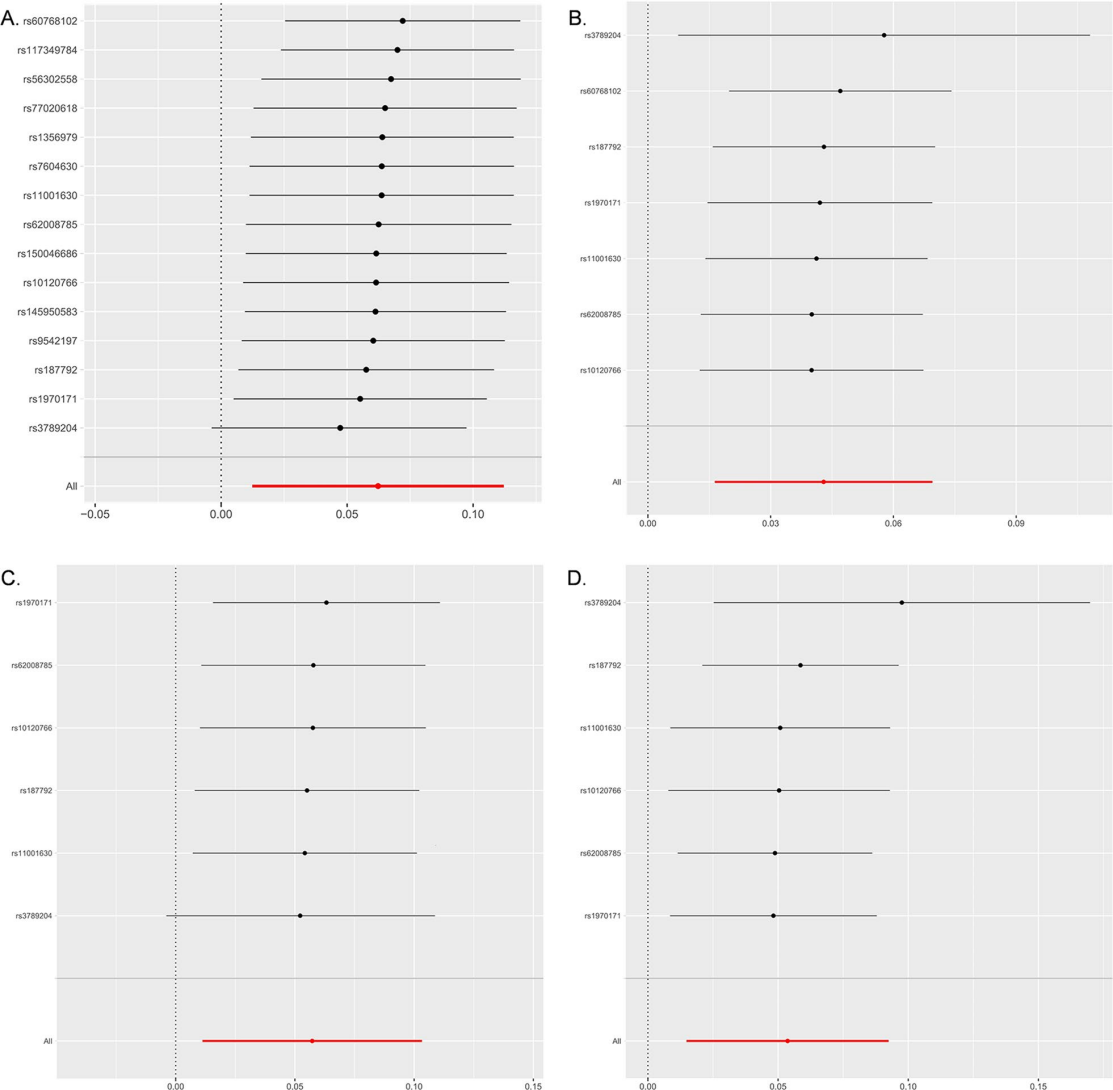
Leave-one-SNP-out sensitivity analysis for plasma sTrem1 on AD (**A**), epilepsy (**B**), generalized epilepsy (**C**) and focal epilepsy (**D**). AD: Alzheimer’s Disease; MR: Mendelian randomization; SNP: single-nucleotide polymorphism; sTrem1: soluble triggering receptor expressed on myeloid cell 1

Under the IVW model, we inferred suggestive evidence that each standard deviation (SD) increase in plasma sTrem1 was related to a higher risk of AD (OR 1.064, 95% CI 1.012–1.119, *P* = 0.014, *P*_*FDR*_ = 0.056) (Fig. 2 and Table 2). As shown in Fig. 3, the scatter plot and forest plot visually displayed the relationships between plasma sTrem1 and AD risk. The associations were almost consistent in sensitivity analysis using the weighted median, MR-PRESSO, and weighted mode (OR 1.089, 95% CI 1.027–1.154, *P* = 0.004; OR 1.064, 95% CI 1.010–1.212, *P* = 0.033; OR 1.084, 95% CI 1.021–1.152, *P* = 0.020, respectively, Table 3). Cochran’s *Q* test and MR-Egger intercept test indicated no heterogeneity (*Q*-value = 16.596, *P*_*Q*_ = 0.278) or horizontal pleiotropy (intercept = − 0.006, *P*_*intercept*_ = 0.653) (Table 4). In addition, the MR-PRESSO global test also failed to reveal substantial pleiotropy (*P* = 0.155). For SNP conformity, we conducted a leave-one-out analysis and generated forest plots (Fig. 4A). The reverse-MR analysis provided no evidence that AD could influence the level of plasma sTrem1 (OR 0.998, 95% CI 0.933–1.068, *P* = 0.949, Additional file 1: Table S2). The F statistics for all the SNPs ranged from 20 to 409 across the MR analysis, higher than the conventional threshold of 10, the rule of thumb to distinguish between strong and weak instrument bias. The R^2^ and F statistic for the IVs are shown in Additional file 1: Table S4.

**Table 2:** Main results of the Mendelian randomization analysis

Outcome	nSNPs	Method	OR (95% CI)	*P *value	*P*_*FDR*_
Neurodegenerative disease					
Alzheimer’s disease	15	IVW	1.064 (1.012 to 1.119)	**0.014**	0.056
Parkinson’s disease	17	IVW	1.027 (0.966 to 1.093)	0.395	0.903
Amyotrophic lateral sclerosis	20	IVW	1.038 (0.996 to 1.080)	0.075	0.240
Multiple sclerosis	11	IVW	1.008 (0.941 to 1.081)	0.811	0.927
Epilepsy					
Epilepsy	7	IVW	1.044 (1.016 to 1.072)	**0.002**	**0.032**
Generalized epilepsy	6	IVW	1.059 (1.011 to 1.109)	**0.013**	0.069
Focal epilepsy	6	IVW	1.055 (1.015 to 1.097)	**0.007**	0.056
Cerebrovascular diseases					
Ischemic stroke	17	IVW	1.012 (0.981 to 1.044)	0.462	0.739
Large-artery atherosclerosis	17	IVW	1.011 (0.934 to 1.094)	0.795	0.978
Small-vessel	16	IVW	0.978 (0.885 to 1.081)	0.662	0.883
Cardioembolic	17	IVW	0.981 (0.915 to 1.051)	0.587	0.854
Nontraumatic intracranial hemorrhage	13	IVW	1.064 (0.975 to 1.162)	0.165	0.440
Subarachnoid hemorrhage	13	IVW	1.057 (0.931 to 1.200)	0.396	0.792
Migraine					
Migraine with aura	17	IVW	1.006 (0.935 to 1.082)	0.879	0.938
Migraine without aura, drug-induced	17	IVW	0.888 (0.650 to 1.213)	0.454	0.807
Migraine without aura and triptan purchases	17	IVW	1.003 (0.928 to 1.084)	0.936	0.936

Bold symbol indicated statistically significances (*P* < 0.05)

CI: confidence interval; FDR: false discovery rate; IVW: inverse-variance weighted; nSNPs: number of single-nucleotide polymorphisms; OR: odds ratio

**Table 3:** Sensitivity analysis of the associations between plasma sTrem1 levels and neurological diseases

Outcome	Weighted Median	MR-PRESSO	MR-Egger	Simple mode	Weighted mode
	OR (95% CI)	OR (95% CI)	OR (95% CI)	OR (95% CI)	OR (95% CI)
	*P* value	*P* value	*P* value	*P* value	*P* value
Neurodegenerative diseases					
Alzheimer’s disease	1.089 (1.027, 1.154)	1.064 (1.010, 1.212)	1.085 (0.986, 1.194)	1.039 (0.926, 1.166)	1.084 (1.021, 1.152)
	**0.004**	**0.033**	0.120	0.526	**0.020**
Parkinson’s disease	1.080 (1.006, 1.160)	1.026 (0.967, 1.089)	1.157 (1.046, 1.280)	0.963 (0.764, 1.214)	1.087 (1.012, 1.168)
	**0.030**	0.401	**0.013**	0.755	**0.035**
Amyotrophic lateral sclerosis	1.037 (0.985, 1.092)	1.034 (1.007, 1.062)	1.055 (0.977, 1.139)	0.998 (0.909, 1.095)	1.035 (0.980, 1.092)
	0.167	0.021	0.189	0.961	0.235
Multiple sclerosis	0.997 (0.934, 1.065)	1.014 (0.953, 1.079)	1.029 (0.903, 1.174)	1.049 (0.902, 1.220)	1.000 (0.934, 1.072)
	0.934	0.667	0.674	0.549	1.000
Epilepsy					
Epilepsy	1.039 (1.008, 1.070)	1.042 (1.019, 1.065)	1.022 (0.971, 1.076)	1.064 (0.997, 1.135)	1.039 (1.008, 1.073)
	**0.012**	**0.008**	0.434	0.110	**0.049**
Generalized epilepsy	1.060 (1.009, 1.114)	1.059 (1.036, 1.083)	1.072 (0.982, 1.170)	1.059 (0.965, 1.161)	1.060 (1.005, 1.117)
	**0.020**	**0.002**	0.196	0.281	0.084
Focal epilepsy	1.052 (1.015, 1.092)	1.050 (1.009, 1.092)	1.001 (0.942, 1.063)	1.133 (1.013, 1.267)	1.041 (1.005, 1.077)
	**0.006**	**0.049**	0.984	0.080	0.074
Cerebrovascular diseases					
Ischemic stroke-all	1.012 (0.973, 1.054)	1.015 (0.985, 1.045)	1.016 (0.958, 1.077)	0.986 (0.906, 1.074)	1.013 (0.973, 1.054)
	0.546	0.357	0.600	0.757	0.548
Large-artery atherosclerosis	0.985 (0.893, 1.086)	1.016 (0.951, 1.085)	0.990 (0.855, 1.147)	0.994 (0.821, 1.204)	0.987 (0.889, 1.096)
	0.765	0.652	0.897	0.952	0.807
Small-vessel	0.977 (0.891, 1.072)	0.977 (0.888, 1.075)	0.958 (0.789, 1.162)	1.027 (0.848, 1.244)	0.981 (0.891, 1.081)
	0.626	0.638	0.669	0.790	0.707
Cardioembolic	0.986 (0.914, 1.064)	0.990 (0.922, 1.062)	1.005 (0.880, 1.147)	0.995 (0.857, 1.154)	0.987 (0.918, 1.062)
	0.721	0.781	0.947	0.943	0.738
Nontraumatic intracranial hemorrhage	1.094 (0.978, 1.223)	1.066 (0.984, 1.155)	1.100 (0.939, 1.288)	1.028 (0.840, 1.258)	1.089 (0.973, 1.218)
	0.116	0.140	0.265	0.793	0.162
Subarachnoid hemorrhage	1.036 (0.891, 1.203)	1.080 (0.960, 1.211)	1.041 (0.831, 1.306)	0.960 (0.715, 1.288)	1.036 (0.883, 1.216)
	0.648	0.225	0.732	0.790	0.671
Migraine					
Migraine with aura	0.944 (0.858, 1.039)	0.988 (0.911, 1.072)	0.919 (0.806, 1.048)	1.049 (0.841, 1.308)	0.944 (0.854, 1.043)
	0.242	0.781	0.228	0.679	0.272
Migraine without aura, drug-induced	0.793 (0.524, 1.199)	0.893 (0.720, 1.108)	0.756 (0.432, 1.322)	1.467 (0.671, 3.206)	0.772 (0.519, 1.150)
	0.272	0.320	0.342	0.351	0.221
Migraine without aura and triptan purchases	0.978 (0.881, 1.085)	0.999 (0.944, 1.058)	1.020 (0.887, 1.173)	0.980 (0.800, 1.200)	0.973 (0.873, 1.086)
	0.671	0.985	0.783	0.847	0.636

Bold symbol indicated statistically significances (*P* < 0.05)

CI: confidence interval; MR-PRESSO: Pleiotropy Residual Sum and Outlier; OR: odds ratio; sTrem1: soluble triggering receptor expressed on myeloid cell 1

**Table 4:** Heterogeneity and pleiotropy tests for the associations of plasma Trem1 levels with neurological diseases

Outcome	Cochrane’s *Q* test		MR-Egger intercept test		MRPRESSO global test
	Q-value	*P*_*Q*_	Intercept	*P*_intercept_	*P *value
Neurodegenerative disease					
Alzheimer’s disease	16.596	0.278	− 0.006	0.653	0.155
Parkinson’s disease	19.716	0.233	− 0.034	**0.014**	0.265
Amyotrophic lateral sclerosis	8.030	0.986	− 0.005	0.624	0.986
Multiple sclerosis	15.002	0.132	− 0.006	0.720	0.292
Epilepsy					
epilepsy	4.352	0.629	0.007	0.399	0.758
Generalized epilepsy	1.460	0.918	− 0.004	0.764	0.973
Focal epilepsy	7.540	0.183	0.018	0.113	0.352
Cerebrovascular diseases					
Ischemic stroke	14.960	0.528	− 0.001	0.869	0.632
Large-artery atherosclerosis	11.741	0.762	0.006	0.753	0.777
Small-vessel	27.771	**0.023**	0.006	0.807	0.068
Cardioembolic	20.504	0.198	− 0.007	0.682	0.245
Nontraumatic intracranial hemorrhage	11.070	0.523	− 0.011	0.638	0.654
Subarachnoid hemorrhage	7.993	0.786	0.005	0.882	0.657
Migraine					
Migraine with aura	13.523	0.634	0.027	0.127	0.229
Migraine without aura, drug-induced	8.291	0.940	0.048	0.508	0.932
Migraine without aura and triptan purchases	9.061	0.911	− 0.005	0.780	0.921

Bold symbol indicated statistically significances (*P* < 0.05)

MR-PRESSO: Pleiotropy Residual Sum and Outlier; sTrem1: soluble triggering receptor expressed on myeloid cell 1

### Associations of plasma sTrem1 levels with epilepsy

As shown in Fig. 2 and Table 2, the IVW model indicated statistically significant evidence of the relationship that plasma sTrem1 (per SD increase) was associated with the risk of epilepsy (OR 1.044, 95% CI 1.016–1.072, *P* = 0.002, *P*_*FDR*_ = 0.032), generalized epilepsy (OR 1.059, 95% CI 1.011–1.109, *P* = 0.013, *P*_*FDR*_ = 0.069), and focal epilepsy (OR 1.055, 95% CI 1.015–1.097, *P* = 0.007, *P*_*FDR*_ = 0.056). In the sensitivity analysis, the weighted median estimator and MR-PRESSO provided consistent results (all *P* values < 0.05, Table 3). In addition, the weighted mode specifically supported a mild causal effect of plasma sTrem1 on epilepsy risk (OR 1.039, 95% CI 1.008–1.073, *P* = 0.049; Table 3). In the analysis of epilepsy, no evidence of heterogeneity or directional pleiotropy were detected (all *P* value > 0.1, Table 4). We also applied the leave-one-out analysis and failed to identify any SNP that substantially influenced the IVW estimate (Fig. 4B–D). The reverse-MR analysis of all subtypes suggested that plasma sTrem1 may not be influenced causally by epilepsy (Additional file 1: Table S2).

No significant results were found for plasma sTrem1 levels with other neurological diseases and their subtypes (Fig. 2 and Table 2). The results of power analysis are presented in Additional file 1: Table S1. In addition, the scatter plot, funnel plot, forest plot and leave-one-out plot were all drawn and shown in Additional file 1: Figs. S1–S15.

## Discussion

The study for the first time presents the bidirectional assessment of the associations between genetically predicted plasma sTrem1 and a range of neurological disorders, using a two-sample MR method. Our findings add to the current knowledge by discovering suggestive association between plasma sTrem1 and AD. Moreover, the causal nature of the associations of increased plasma sTrem1 with higher risk of epilepsy was established. However, no evidence was indicated for the causal relationships between plasma sTrem1 and other evaluated neurological disorders, as well as their subtypes, such as PD, ALS, MS, cerebrovascular diseases, and migraine.

Trem1, encoded by a gene mapped to human chromosome 6, is a member of the immunoglobulin Trem superfamily [39]. It is widely expressed by a subset of myeloid cells, including monocytes and microglia [11, 39]. Two forms of the receptor, membrane and soluble (mTrem1 and sTrem1, respectively) have been identified in the human body [40]. mTrem1 is composed of a single extracellular Ig-like domain, a transmembrane structure and an intracellular domain [41]. mTrem1 participates in immune responses via its interaction with classic pattern recognition receptors, such as Toll-like receptors and Nod-like receptors [41]. It amplifies the production of proinflammatory factors, such as interleukin-8 (IL-8), monocyte chemoattractant protein-1 (MCP-1) and oxidative components [39]. Emerging evidence has indicated that sTrem1 can be found in body fluids, including plasma, CSF and bronchoalveolar fluids [12, 13, 15], although its origin remains unclear. It is believed that the soluble form of Trem1 can be generated from the proteolytic cleavage of mTrem1 by metalloproteinases or from the translation of an alternative spliced mRNA [40]. Studies have found increased concentrations of sTrem1 in plasma and CSF of AD patients [12, 13]. Moreover, a gradual increase of the molecule in plasma was observed during disease progression [12]. The findings from observational studies seem to echo our estimates for the effects of plasma sTrem1 that the per one SD increment in the genetically determined plasma sTrem1 corresponded to a 6.4% increase in the risk of AD. This indicated a detrimental effect of the increasement of sTrem1 on the disorder. Although the association analysis did not reach significance level after correction for multiple testing (*P*_FDR_ = 0.056), it implies suggestive involvement of sTrem1 in the risk of AD. In addition, The Benjamini–Hochberg FDR assumes that all statistical tests have the similar ability to detect potential discoveries [42]. However, FDR estimation is subject to variability due to difference in underlying biology, signal-to-noise ratio or features of trait, which can lead to greater power than others in certain tests [42, 43]. The link between sTrem1 and AD was further emphasized by the positive correlation between elevated sTrem1 in CSF and typical biomarkers of AD, including Aβ42, t-tau and p-tau [13]. The evidence, along with our MR estimates, concordantly supported the possibility of causal pathways by increased sTrem1 in CSF and progressive Alzheimer’s pathology.

**Fig. 5:**
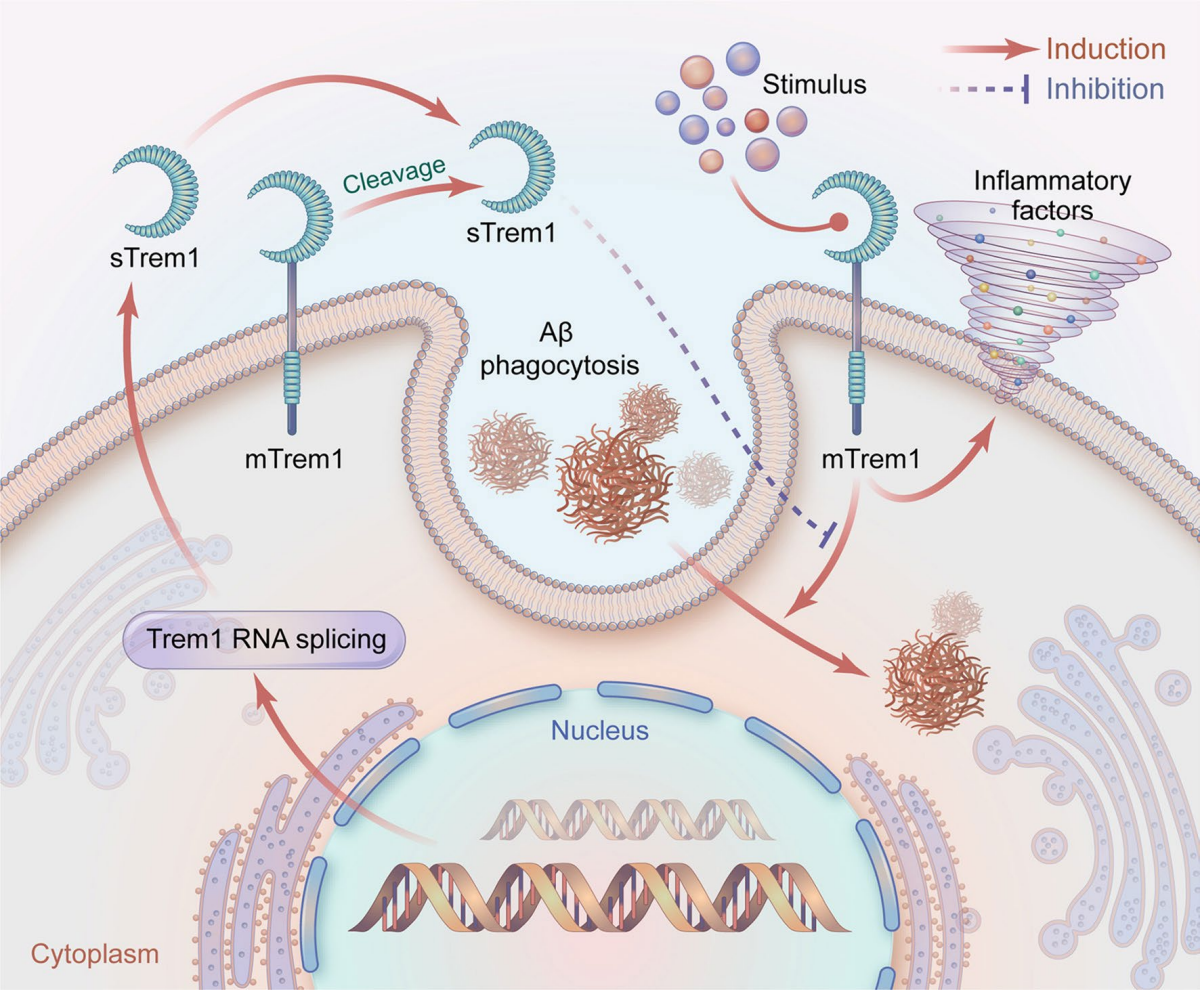
A schematic representation of sTrem1 function in AD. Ligands combine with mTrem1 on immune cells to induce the inflammatory responses and phagocytosis of Aβ. sTrem1 comes from the proteolytic cleavage of mTrem1 by metalloproteinases or from the translation of an alternative splicing of Trem1 mRNA. sTrem1 may perform as a decoy peptide to inhibit the binding of mTrem1 with the ligands. Subsequently, the clearance of Aβ gets inhibited, inducing the accumulation of the protein in brain. AD, Alzheimer’s disease; mTrem1: membrane triggering receptor expressed on myeloid cell 1; sTrem1: soluble triggering receptor expressed on myeloid cell 1

Aβ accumulation is one of the key pathological changes in AD [44]. It is believed that increasing Aβ burden is responsible for neuronal death and brain atrophy [45, 46]. Patients with accelerated Aβ deposition tend to show a higher rate of cognitive decline [47]. Studies have further indicated that the clearance of Aβ should be an effective way to alleviate neurodegeneration and cognitive impairment [48]. The possibility of the involvement of mTrem1 in Aβ clearance was raised, which may help the understanding of its role in AD. It was reported that mTrem1 could regulate microglia phagocytosis and facilitate Aβ clearance in the brains of APP/PS1 mice [10]. Knockdown of the gene may exacerbate Aβ pathology in the brain, which could be ameliorated by selective overexpression, indicating the protective role of mTrem1 targeting Aβ elimination. However, it might seem odd when we consider the associations of sTrem1 in body fluids with AD biomarkers. The soluble molecule is likely to be regarded as indicative of AD pathology; thus, there is a discrepancy between different forms of Trem1 in terms of their roles in AD. Herein, we may say that the effects of sTrem1 and mTrem1 may be in opposition during AD progression. Previous studies have provided support for our speculation by discovering that sTrem1 acts as a decoy peptide to inhibit the binding of mTrem1 with ligands [7], which might block the subsequent coupling activities with intracellular signaling proteins, and inhibit the phagocytosis of Aβ by immune cells. In addition, rs6910730^G^ within the *TREM1 gene* was found to be associated with an increased burden of neuritic plaques, Aβ deposition and cognitive decline in AD patients [49]. Another study using the Alzheimer’s Disease Neuroimaging Initiative (ADNI) database indicated that the rs2234246^A^ variant is responsible for brain amyloidosis [50]. Therefore, increasing sTrem1 or the specific variants could account for the interrupted mTrem1 operation, which participates in Aβ clearance to reduce AD pathology. The schematic interaction between sTrem1 and mTrem1 is depicted in Fig. 5.

Tau pathology is another typical feature of AD. Biomarkers, including p-tau and t-tau, can be detected in body fluid, and used to indicate CNS tauopathy, disease severity and progression. The associations between sTrem1 and the two markers have been confirmed [12, 13]. Moreover, Garwood et al. [51] found increased expression of sTrem1 in cortical lysates from a tauopathy mouse model. Another study shed light on the involvement of mTrem1 in diabetes-associated hyperphosphorylation in mouse brain [52]. However, the above studies only indicated the possible link between mTrem1 or sTrem1 and tauopathy with their observational evidence. The precise effects of Trem1 on tauopathy remain unclear. Studies should be performed to reveal the clear pattern.

Interestingly, given the risk for epilepsy as the outcome, the per one SD increment in plasma sTrem1 corresponded to a 4.4% increase of the disease risk. In addition, elevated sTrem1 increased odds of generalized epilepsy and focal epilepsy by 5.9% and 5.5%, respectively. However, the adjustment for multiple testing of generalized (*P*_FDR_ = 0.069) and focal epilepsy (*P*_FDR_ = 0.056) did not reach the corrected significance level within all evaluated diseases, providing the suggestive relationship between sTrem1 and epileptic subtypes. Epilepsy is one of the most common serious neurological conditions [53]. It is widely accepted that neurological insults predispose patients to a hyperexcitable network in the brain, inducing recurrent seizures [54]. Severe inflammation can be seen in the epileptogenic lesions [55]. Although the importance of sTrem1 in neuroinflammation has been addressed, the potential mechanism remains unknown. Our explorative work is an important supplement to reveal the role of sTrem1 in epilepsy. Considering the effects of sTrem1 in neuroinflammation, an interaction of the protein, epileptic stimulus and the surrounding inflammatory components should be responsible for the potential causality. sTrem1 can modulate the inflammatory responses of immune cells to influence the homeostasis of neuronal cells. Moreover, it may not be a specific marker for epilepsy, but could be a useful indicator to demonstrate the inflammatory conditions of the disease. However, we should interpret the findings with caution, due to the lack of further clinical and basic supporting studies.

We failed to discover a causality between plasma sTrem1 and PD, ALS, MS, cerebrovascular disease, as well as migraine. And moderate levels of MR power were indicated in PD (63%) and ALS (67%), which are among the most neurodegenerative diseases, characterized by protein aggregation [56, 57]. The inconsistent findings with AD under the neurodegeneration continuum may be attributed to the fact that the two diseases do not fall into an amyloid- or tauopathy-dominated category. We speculated that the effects of sTrem1 might depend on the pathological stimulus. A potential interactive mode between sTrem1 and the specific initiating agent should be explored. Indeed, because of the limited power of the analysis, we cannot preclude from the null associations that plasma sTrem1 having small or no effects on risks of MS, cerebrovascular disorders and migraine. Additionally, studies have reported Trem1 as an indicator of neuroinflammation across various brain disorders. In an ischemic stroke model, mTrem1 activated the NF-κB signaling pathway and NLRP3 inflammasome via its interaction with SYK in microglia [11]. Besides, the protein could be adopted as a reliable PET probe to visualize the presence of early immune activation in PD and MS [58, 59]. Therefore, neuroinflammation as the thread connecting the above diseases may explain the null results in our study. Higher plasma sTrem1 may be a concomitant factor along with inflammation in these disorders.

A key strength of the study is that we examined the genetic analysis of the effects of plasma sTrem1 on neurological disorders by exploring data from relatively large GWAS data sources. Another strength of the study is that the wide range of the included diseases ensures the general understanding of the role of plasma sTrem1 across neurological continuum under MR frameworks. Moreover, MR study needs to be checked adequately for horizontal pleiotropy and heterogeneity to ensure its own assumptions. We addressed the analysis of horizontal pleiotropy by performing MR-PRESSO global test and MR-Egger intercept test, and by the visual inspection of funnel plot, indicating no evidence of horizontal pleiotropic effect in AD and epilepsy. The robustness of the significant findings was further validated by detecting heterogeneity using Cochrane’s Q test and leave-one-out sensitivity analysis. Inevitably, there are also some limitations in the study. First, causal results in the study were novel and promising; however, findings in relation to several common types of neurological disorders tend to be accompanied by small statistical power (Additional file 1: Table S1), which shrunk the credibility of the results. The limited power across these diseases may be attributed to an insufficient sample size. This generated an impetus to perform MR studies on larger sample size populations. Second, the genetic variants associated with plasma sTrem1 may not be ideal if aging or other related factors may affect the activities of the molecule. We failed to comprehensively assess associations of individual genetic variants with potential confounding factors due to the lack of knowledge of individual data and adjusting factors. These findings need further in vitro and in vivo validation. Third, most of the GWAS data were from the European population, which implicated the careful application of our findings to other racial/ethnic populations. Fourth, although proteins in human plasma are effective and convenient biomarkers in clinical practice, they may not directly and fully demonstrate the changes in brain, because of the existence of the blood–brain barrier. Further exploration of the role of CSF sTrem1 or the protein from intracellular environment could be a better choice.

## Conclusions

In conclusion, the MR study for the first time indicates suggestive association between genetic predisposition to plasma sTrem1 and risk of AD. Moreover, it identified the potential causality between higher plasma sTrem1 and increased risk of epilepsy. Irrespective of the exact mechanism of the associations via a genetic background, our study provides novel insight for understanding the role of sTrem1 in neurological disorders.

## Supplementary Information

**Additional file 1: Table S1.** Power calculation for two-sample MR analyses of sTrem1 on neurological diseases. **Table S2.** Reverse causal relations of plasma sTrem1 with neurological diseases performed by MR. **Table S3.** Details of the removed SNPs for potential horizontal pleiotropy. **Table S4.** Characteristics of selected SNPs for plasma sTrem1. **Table S5.** Characteristics of selected SNPs for neurodegenerative diseases. **Table S6.** Characteristics of selected SNPs for multiple sclerosis. **Table S7.** Characteristics of selected SNPs for epilepsy. **Table S8.** Characteristics of selected SNPs for cerebrovascular diseases. **Table S9.** Characteristics of selected SNPs for migraine. **Figure S1.** The causal effect of plasma sTrem1 on Alzheimer’s Disease risk. **Figure S2.** The causal effect of plasma sTrem1 on Parkinson’s Disease risk. **Figure S3.** The causal effect of plasma sTrem1 on Amyotrophic lateral sclerosis risk. **Figure S4.** The causal effect of plasma sTrem1 on Multiple sclerosis risk. **Figure S5.** The causal effect of plasma sTrem1 on epilepsy risk. **Figure S6.** The causal effect of plasma sTrem1 on generalized epilepsy risk. **Figure S7.** The causal effect of plasma sTrem1 on focal epilepsy risk. **Figure S8.** The causal effect of plasma sTrem1 on ischemic stroke risk. **Figure S9.** The causal effect of plasma sTrem1 on ischemic stroke (large-artery atherosclerosis) risk. **Figure S10.** The causal effect of plasma sTrem1 on ischemic stroke (cardioembolic) risk. **Figure S11.** The causal effect of plasma sTrem1 on nontraumatic intracranial hemorrhage risk. **Figure S12. **The causal effect of plasma sTrem1 on subarachnoid haemmorrhage risk. **Figure S13.** The causal effect of plasma sTrem1 on migraine (with aura) risk. **Figure S14.** The causal effect of plasma sTrem1 on migraine (without aura, drug-induced) risk. **Figure S15.** The causal effect of plasma sTrem1 on migraine (without aura and triptan purchases) risk.

**Additional file 2.** Information of investigators of each GWAS dataset in the MR study.

## Data Availability

All data used in the current study were extracted from public genome-wide association study summary statistics.

## Abbreviations

AD: Alzheimer’s disease
ADNI: Alzheimer’s Disease Neuroimaging Initiative
ALS: Amyotrophic lateral sclerosis
CES: Cardioembolic stroke
CI: Confidence interval
CNS: Central nervous system
CSF: Cerebrospinal fluid
GWAS: Genome-wide association studies
IGAP: International Genomics of Alzheimer's Project
IL-8: Interleukin-8
ILAE: International League Against Epilepsy
IMSGC: International Multiple Sclerosis Genetics Consortium
IPDGC: International Parkinson's Disease Genomics Consortium
IVW: Inverse-variance weighted
LAS: Large-artery atherosclerosis stroke
MCP-1: Monocyte chemoattractant protein-1
MR: Mendelian randomization
MR-PRESSO: MR-pleiotropy residual sum and outlier
MS: Multiple sclerosis
mTrem1: Membrane triggering receptor expressed on myeloid cell 1
OR: Odds ratio
PD: Parkinson’s disease
SNP: Single-nucleotide polymorphism
sTrem1: Soluble triggering receptor expressed on myeloid cell 1
SVS: Small-vessel stroke
Trem1: Triggering receptor expressed on myeloid cell 1
TYROBP: TYRO protein tyrosine kinase-binding protein
