# Supplementary material for: The associations between plasma soluble Trem1 and neurological diseases: a Mendelian randomization study
Source: J Neuroinflammation. 2022 Sep 6;19:218. doi: 10.1186/s12974-022-02582-z (PMC9446564; doi:10.1186/s12974-022-02582-z)
Supplement: Supplementary file 1 — Additional file 1: Table S1. Power calculation for two-sample MR analyses of sTrem1 on neurological diseases. Table S2. Reverse causal relations of plasma sTrem1 with neurological diseases performed by MR. Table S3. Details of the removed SNPs for potential horizontal pleiotropy. Table S4. Characteristics of selected SNPs for plasma sTrem1. Table S5. Characteristics of selected SNPs for neurodegenerative diseases. Table S6. Characteristics of selected SNPs for multiple sclerosis. Table S7. Characteristics of selected SNPs for epilepsy. Table S8. Characteristics of selected SNPs for cerebrovascular diseases. Table S9. Characteristics of selected SNPs for migraine. Figure S1. The causal effect of plasma sTrem1 on Alzheimer’s Disease risk. Figure S2. The causal effect of plasma sTrem1 on Parkinson’s Disease risk. Figure S3. The causal effect of plasma sTrem1 on Amyotrophic lateral sclerosis risk. Figure S4. The causal effect of plasma sTrem1 on Multiple sclerosis risk. Figure S5. The causal effect of plasma sTrem1 on epilepsy risk. Figure S6. The causal effect of plasma sTrem1 on generalized epilepsy risk. Figure S7. The causal effect of plasma sTrem1 on focal epilepsy risk. Figure S8. The causal effect of plasma sTrem1 on ischemic stroke risk. Figure S9. The causal effect of plasma sTrem1 on ischemic stroke (large-artery atherosclerosis) risk. Figure S10. The causal effect of plasma sTrem1 on ischemic stroke (cardioembolic) risk. Figure S11. The causal effect of plasma sTrem1 on nontraumatic intracranial hemorrhage risk. Figure S12. The causal effect of plasma sTrem1 on subarachnoid haemmorrhage risk. Figure S13. The causal effect of plasma sTrem1 on migraine (with aura) risk. Figure S14. The causal effect of plasma sTrem1 on migraine (without aura, drug-induced) risk. Figure S15. The causal effect of plasma sTrem1 on migraine (without aura and triptan purchases) risk. [file 12974_2022_2582_MOESM1_ESM.docx]

**Supplementary files**

**Supplementary Tables**

Table S1. Power calculation for two-sample MR analyses of sTrem1 on neurological diseases ……………………………………………………….............…………………3

Table S2. Reverse causal relations of plasma sTrem1 with neurological diseases performed by MR ………………………………………………………….…………………4

Table S3. Details of the removed SNPs for potential horizontal pleiotropy …………………………………………………………………………………….…………………7

Table S4. Characteristics of selected SNPs for plasma sTrem1………………………………………………………………………………………………….…………………8

Table S5. Characteristics of selected SNPs for neurodegenerative diseases……………………………………………………………………………………..…………………9

Table S6. Characteristics of selected SNPs for multiple sclerosis …………………………………………………………………………………….………...…………………11

Table S7. Characteristics of selected SNPs for epilepsy…………………………………………………………………………………….………....………...………………..14

Table S8. Characteristics of selected SNPs for cerebrovascular diseases………………………………………………………………………………………..………………..15

Table S9. Characteristics of selected SNPs for migraine………………………………………………………………………………………………………………………….18

**Supplementary Figures**

Figure S1. The causal effect of plasma sTrem1 on Alzheimer’s Disease risk…………………………………………………………………………………….………………21

Figure S2. The causal effect of plasma sTrem1 on Parkinson’s Disease risk……………………………………………………………………………………..………………22

Figure S3. The causal effect of plasma sTrem1 on Amyotrophic lateral sclerosis risk…………………………………………………………………………..………………23

Figure S4. The causal effect of plasma sTrem1 on Multiple sclerosis risk………………………………………………………………………………………………………24

Figure S5. The causal effect of plasma sTrem1 on epilepsy risk ………………………………………………………………………………………………..………………25

Figure S6. The causal effect of plasma sTrem1 on generalized epilepsy risk……………………………………………………………………………………………………26

Figure S7. The causal effect of plasma sTrem1 on focal epilepsy risk…………………………………………………………………………………………..………………27

Figure S8. The causal effect of plasma sTrem1 on ischemic stroke risk…………………………………………………………………………………………………………28

Figure S9. The causal effect of plasma sTrem1 on ischemic stroke (large artery atherosclerosis) risk………………………………………………………….………………29

Figure S10. The causal effect of plasma sTrem1 on ischemic stroke (cardioembolic) risk………………………………………………………………………………………30

Figure S11. The causal effect of plasma sTrem1 on nontraumatic intracranial hemorrhage risk………………………………………………………………..………………31

Figure S12. The causal effect of plasma sTrem1 on subarachnoid haemmorrhage risk…………………………………………………………………………………………32

Figure S13. The causal effect of plasma sTrem1 on migraine (with aura) risk…………………………………………………………………………………………………..33

Figure S14. The causal effect of plasma sTrem1 on migraine (without aura, drug-induced) risk…………………………………………………………….………………….34

Figure S15. The causal effect of plasma sTrem1 on migraine (without aura and triptan purchases) risk……………………………………………………….……………….35

**Supplementary Tables**

**Table S1.** Power calculation for two-sample MR analysis of sTrem1 on neurological diseases.

| **Outcome** | **Sample size** | **Proportion of cases** | **OR** | **R^2^** | **Power** |
| --- | --- | --- | --- | --- | --- |
| **Neurodegenerative disease** |  |  |  |  |  |
| Alzheimer’s Disease | 63,926 | 0.34 | 1.064 | 0.234 | 95% |
| Parkinson’s Disease | 482,730 | 0.07 | 1.027 | 0.228 | 63% |
| Amyotrophic lateral sclerosis | 80,610 | 0.26 | 1.038 | 0.260 | 67% |
| **Multiple sclerosis** | 115,803 | 0.41 | 1.008 | 0.196 | 9% |
| **Epilepsy** |  |  |  |  |  |
| epilepsy | 44,889 | 0.34 | 1.044 | 0.159 | 41% |
| -generalized epilepsy | 33,446 | 0.11 | 1.059 | 0.152 | 26% |
| -focal epilepsy | 39,348 | 0.25 | 1.055 | 0.152 | 44% |
| **Cerebrovascular diseases** |  |  |  |  |  |
| Ischemic stroke | 440,328 | 0.08 | 1.012 | 0.233 | 18% |
| -large artery atherosclerosis | 410,484 | 0.01 | 0.991 | 0.233 | 6% |
| -small-vessel | 198,048 | 0.03 | 0.977 | 0.226 | 5% |
| -cardioembolic | 413,304 | 0.02 | 0.981 | 0.233 | 13% |
| Nontraumatic intracranial hemorrhage | 205,862 | 0.01 | 1.064 | 0.208 | 26% |
| Subarachnoid hemorrhage | 202,568 | 0.01 | 1.057 | 0.208 | 21% |
| **Migraine** |  |  |  |  |  |
| Migraine with aura | 179,648 | 0.02 | 1.006 | 0.238 | 5% |
| Migraine without aura, drug-induced | 218,792 | 0.001 | 0.888 | 0.238 | 13% |
| Migraine without aura and triptan purchases | 167,313 | 0.02 | 1.003 | 0.238 | 5% |

MR, Mendelian randomization; OR, odds ratio; sTrem1, soluble Triggering receptor expressed on myeloid cell 1. Type-I error rate (α) was set as 0.05.

**Table S2.** Reverse causal relationships of plasma sTrem1 with neurological diseases performed by MR.

| **Exposure** | **nSNPs** | **Method** | **OR (95%CI)** | ***P value*** | **Q pval** | **intercept**  ***p* value** | **Global *P*** |
| --- | --- | --- | --- | --- | --- | --- | --- |
| Alzheimer’ s Disease | 18 | IVW | 0.998 (0.933, 1.068) | 0.949 | 0.397 |  |  |
|  |  | MR Egger | 0.997 (0.906, 1.097) | 0.951 |  | 0.981 |  |
|  |  | MR-PRESSO | 0.983 (0.909, 1.062) | 0.669 |  |  | 0.155 |
|  |  | WM | 0.958 (0.877, 1.047) | 0.342 |  |  |  |
|  |  | Simple mode | 0.965 (0.832, 1.121) | 0.650 |  |  |  |
|  |  | Weighted mode | 0.959 (0.883, 1.041) | 0.332 |  |  |  |
| Parkinson’s Disease | 20 | IVW | 1.013 (0.938, 1.094) | 0.737 | 0.422 |  |  |
|  |  | MR Egger | 0.930 (0.773, 1.119) | 0.449 |  | 0.328 |  |
|  |  | MR-PRESSO | 1.012 (0.940, 1.090) | 0.746 |  |  | 0.430 |
|  |  | WM | 1.054 (0.938, 1.1826) | 0.377 |  |  |  |
|  |  | Simple mode | 1.120 (0.886, 1.416) | 0.354 |  |  |  |
|  |  | Weighted mode | 1.102 (0.886, 1.371) | 0.391 |  |  |  |
| Amyotrophic lateral sclerosis | 6 | IVW | 1.098 (0.865, 1.395) | 0.442 | 0.157 |  |  |
|  |  | MR Egger | 1.336 (0.738, 2.419) | 0.393 |  | 0.514 |  |
|  |  | MR-PRESSO | 1.098 (0.865, 1.400) | 0.477 |  |  | 0.201 |
|  |  | WM | 1.075 (0.825, 1.400) | 0.593 |  |  |  |
|  |  | Simple mode | 0.994 (0.690, 1.433) | 0.976 |  |  |  |
|  |  | Weighted mode | 1.008 (0.733, 1.386) | 0.962 |  |  |  |
| Multiple sclerosis | 65 | IVW | 1.034 (0.995, 1.074) | 0.088 | 0.730 |  |  |
|  |  | MR Egger | 1.000 (0.943, 1.060) | 0.993 |  | 0.144 |  |
|  |  | MR-PRESSO | 1.038 (1.001, 1.075) | **0.046** |  |  | 0.304 |
|  |  | WM | 1.033 (0.972, 1.098) | 0.297 |  |  |  |
|  |  | Simple mode | 1.109 (0.971, 1.266) | 0.131 |  |  |  |
|  |  | Weighted mode | 1.033 (0.972, 1.098) | 0.299 |  |  |  |
| Epilepsy | 3 | IVW | 1.011 (0.517, 1.979) | 0.973 | 0.293 |  |  |
|  |  | MR Egger | 0.494 (0.018, 13.465) | 0.748 |  | 0.736 |  |
|  |  | MR-PRESSO | 1.048 (1.002, 1.095) | **0.048** |  |  | 0.304 |
|  |  | WM | 1.093 (0.538, 2.221) | 0.806 |  |  |  |
|  |  | Simple mode | 1.571 (0.544, 4.537) | 0.492 |  |  |  |
|  |  | Weighted mode | 1.562 (0.548, 4.454) | 0.492 |  |  |  |
| Focal epilepsy | 1 | Wald ratio | 0.611 (0.234, 1.599) | 0.316 |  |  |  |
| Generalized epilepsy | 11 | IVW | 0.991 (0.808, 1.214) | 0.927 | 0.426 |  |  |
|  |  | MR Egger | 0.389 (0.062, 2.431) | 0.339 |  | 0.341 |  |
|  |  | MR-PRESSO | 0.991 (0.808, 1.214) | 0.929 |  |  | 0.427 |
|  |  | WM | 0.979 (0.733, 1.282) | 0.828 |  |  |  |
|  |  | Simple mode | 0.999 (0.621, 1.608) | 0.997 |  |  |  |
|  |  | Weighted mode | 1.003 (0.616, 1.635) | 0.989 |  |  |  |
| Ischemic stroke | 9 | IVW | 1.052 (0.811, 1.364) | 0.704 | 0.389 |  |  |
|  |  | MR Egger | 0.913 (0.122, 6.820) | 0.931 |  | 0.893 |  |
|  |  | MR-PRESSO | 1.052 (0.811, 1.364) | 0.714 |  |  | 0.400 |
|  |  | WM | 1.066 (0.759, 1.499) | 0.711 |  |  |  |
|  |  | Simple mode | 1.265 (0.680, 2.355) | 0.479 |  |  |  |
|  |  | Weighted mode | 1.271 (0.660, 2.451) | 0.494 |  |  |  |
| Ischemic stroke -LAS | 3 | IVW | 1.015 (0.834, 1.235) | 0.882 | 0.338 |  |  |
|  |  | MR Egger | 0.301 (0.056, 1.611) | 0.394 |  | 0.389 |  |
|  |  | MR-PRESSO | 0.951 (0.891, 1.014) | 0.224 |  |  | 0.914 |
|  |  | WM | 1.062 (0.844, 1.337) | 0.607 |  |  |  |
|  |  | Simple mode | 1.144 (0.837, 1.563) | 0.489 |  |  |  |
|  |  | Weighted mode | 1.146 (0.818, 1.607) | 0.510 |  |  |  |
| Ischemic stroke-SVS | 2 | IVW | 1.100 (0.748, 1.619) | 0.628 | 0.157 |  |  |
|  |  | MR-PRESSO | 0.951 (0.891, 1.014) | 0.224 |  |  | 0.914 |
| Ischemic stroke-CES | 4 | IVW | 0.951 (0.826, 1.095) | 0.485 | 0.890 |  |  |
|  |  | MR Egger | 1.048 (0.791, 1.389) | 0.774 |  | 0.515 |  |
|  |  | MR-PRESSO | 0.951 (0.891, 1.014) | 0.224 |  |  | 0.914 |
|  |  | WM | 0.959 (0.812, 1.132) | 0.620 |  |  |  |
|  |  | Simple mode | 0.958 (0.766, 1.197) | 0.729 |  |  |  |
|  |  | Weighted mode | 0.964 (0.802, 1.159) | 0.724 |  |  |  |
| Non-traumatic intracranial hemorrhage | 20 | IVW | 1.025 (0.938, 1.120) | 0.581 | 0.109 |  |  |
|  |  | MR Egger | 1.132 (0.950, 1.350) | 0.183 |  | 0.219 |  |
|  |  | MR-PRESSO | 1.025 (0.938, 1.120) | 0.587 |  |  | 0.086 |
|  |  | WM | 0.964 (0.863, 1.076) | 0.511 |  |  |  |
|  |  | Simple mode | 0.938 (0.771, 1.142) | 0.532 |  |  |  |
|  |  | Weighted mode | 0.922 (0.756, 1.124) | 0.433 |  |  |  |
| Subarachnoid hemorrhage | 19 | IVW | 1.002 (0.958, 1.048)) | 0.936 | 0.955 |  |  |
|  |  | MR Egger | 0.996 (0.927, 1.051) | 0.902 |  | 0.808 |  |
|  |  | MR-PRESSO | 1.001 (0.965, 1.039) | 0.941 |  |  | 0.807 |
|  |  | WM | 0.987 (0.881, 1.078) | 0.680 |  |  |  |
|  |  | Simple mode | 0.974 (0.881, 1.078) | 0.618 |  |  |  |
|  |  | Weighted mode | 0.983 (0.923, 1.047) | 0.595 |  |  |  |
| Migraine with aura | 18 | IVW | 1.015 (0.925, 1.114) | 1.114 | 0.621 |  |  |
|  |  | MR Egger | 1.041 (0.858, 1.265) | 0.687 |  | 0.775 |  |
|  |  | MR-PRESSO | 1.015 (0.931, 1.107) | 0.732 |  |  | 0.636 |
|  |  | WM | 1.013 (0.888, 1.156) | 0.850 |  |  |  |
|  |  | Simple mode | 1.070 (0.842, 1.359) | 0.588 |  |  |  |
|  |  | Weighted mode | 1.034 (0.850, 1.259) | 0.740 |  |  |  |
| Migraine without aura, drug-induced | 7 | IVW | 1.007 (0.967, 1.048) | 0.741 | 0.147 |  |  |
|  |  | MR Egger | 0.964 (0.898, 1.035) | 0.357 |  | 0.215 |  |
|  |  | MR-PRESSO | 1.008 (0.995, 1.021) | 0.285 |  |  | 0.579 |
|  |  | WM | 1.001 (0.959, 1.045) | 0.948 |  |  |  |
|  |  | Simple mode | 1.011 (0.955, 1.069) | 0.726 |  |  |  |
|  |  | Weighted mode | 1.005 (0.950, 1.063) | 0.861 |  |  |  |
| Migraine without aura and triptan purchases | 24 | IVW | 1.042 (0.969, 1.120) | 0.268 | 0.759 |  |  |
|  |  | MR Egger | 0.937 (0.811, 1.082) | 0.385 |  | 0.109 |  |
|  |  | MR-PRESSO | 1.042 (0.977, 1.111) | 0.222 |  |  | 0.745 |
|  |  | WM | 0.997 (0.891, 1.117) | 0.961 |  |  |  |
|  |  | Simple mode | 0.960 (0.799, 1.153) | 0.669 |  |  |  |
|  |  | Weighted mode | 0.969 (0.839, 1.119) | 0.674 |  |  |  |

CES, cardioembolic; CI, confidence interval; IVW, inverse-variance weighted; LAS, large artery atherosclerosis; MR, Mendelian randomization; MR-PRESSO, Pleiotropy Residual Sum and Outlier; nSNPs, number of single nucleotide polymorphisms; OR, odds ratio; SVS, small-vessel; Q_pval, *P*-value of the Cochran Q statistic; WM, weighted median.

**Table S3.** Details of the removed SNPs for potential horizontal pleiotropy.

| **SNP** | **Chr** | **Pos.** | **Effect allele** | **Other allele** | **Trait** | **Type** | **Related disease** | ***P value*** |
| --- | --- | --- | --- | --- | --- | --- | --- | --- |
| rs1044856 | 13 | 43462422 | T | A | Gene expression of *EPSTI1* | Gene expression | Epilepsy (unspecified) | 7.92E-63 |
| rs56302558 | 6 | 41165271 | A | G | Gene expression of *ADCY10P1* | Gene expression | Cerebrovascular disease (unspecified) | 5.72E-08 |
| rs138486985 | 3 | 40193475 | C | T | Cause of death: cerebrovascular disease, unspecified | Diseases and traits | Cerebrovascular disease (unspecified) | 4.95E-06 |
| rs1356979 | 11 | 18219240 | T | C | Gene expression of *SAAL1* | Gene expression | Cerebrovascular disease (unspecified) | 2.75E-14 |
| rs62008785 | 14 | 102932572 | A | G | Gene expression of *TECPR2* | Gene expression | Cerebrovascular disease (nontraumatic intracranial hemorrhage) | 3.35E-14 |
| rs187792 | 7 | 158770822 | T | C | Gene expression of *WDR60* | Gene expression | Cerebrovascular disease (subarachnoid hemorrhage) | 1.02E-24 |

ADCY10P1, Adenylyl cyclase 10 Pseudogene 1; Chr, chromosome; EPSTI1, Epithelial Stromal Interaction 1; Pos, position; SAAL1, Serum Amyloid A Like 1; SNP, single-nucleotide polymorphism; TECPR2, Tectonin Beta-Propeller Repeat Containing 2; WDR60. The threshold was set at *P* < 5×10^-8^.

**Table S4.** Characteristics of selected SNPs for plasma sTrem1.

| **SNP** | **Trait** | **Chr** | **Pos.** | **Effect allele** | **Other allele** | **EAF** | **Beta** | **SE** | ***P value*** | **R^2^** | **F statistic** |
| --- | --- | --- | --- | --- | --- | --- | --- | --- | --- | --- | --- |
| rs7604630 | sTrem1 | 2 | 53583585 | G | A | 0.98208 | 0.5287 | 0.1024 | 2.39883E-07 | 0.009839 | 32.78007 |
| rs111215245 | sTrem1 | 3 | 197899868 | A | G | 0.2369 | 0.1303 | 0.029 | 7.07946E-06 | 0.006139 | 20.37613 |
| rs150046686 | sTrem1 | 3 | 94952138 | T | C | 0.03046 | -0.3605 | 0.0811 | 8.70964E-06 | 0.007676 | 25.51908 |
| rs138486985 | sTrem1 | 3 | 40193475 | T | C | 0.00953 | -0.6341 | 0.1401 | 6.0256E-06 | 0.007591 | 25.23313 |
| rs77020618 | sTrem1 | 3 | 151841388 | G | T | 0.03599 | -0.3292 | 0.0667 | 7.94328E-07 | 0.00752 | 24.99617 |
| rs10059982 | sTrem1 | 5 | 178263716 | A | T | 0.03506 | -0.3733 | 0.0772 | 1.31826E-06 | 0.009429 | 31.40182 |
| rs116425365 | sTrem1 | 5 | 161744600 | T | A | 0.05876 | -0.2388 | 0.0526 | 5.62341E-06 | 0.006308 | 20.94167 |
| rs56302558 | sTrem1 | 6 | 41165271 | A | G | 0.17819 | -0.1889 | 0.0333 | 1.44544E-08 | 0.010451 | 34.84124 |
| rs3789204 | sTrem1 | 6 | 41254741 | T | G | 0.29265 | 0.5163 | 0.0261 | 4.2658E-87 | 0.110361 | 409.2473 |
| rs117349784 | sTrem1 | 7 | 81315786 | A | G | 0.03752 | -0.3111 | 0.0704 | 9.77237E-06 | 0.00699 | 23.22273 |
| rs187792 | sTrem1 | 7 | 158770822 | T | C | 0.04185 | -0.2823 | 0.0624 | 6.0256E-06 | 0.006391 | 21.22011 |
| rs694028 | sTrem1 | 8 | 68556007 | T | A | 0.56181 | 0.1096 | 0.0247 | 9.12011E-06 | 0.005914 | 19.62734 |
| rs60768102 | sTrem1 | 8 | 96197515 | C | T | 0.10117 | 0.1879 | 0.0422 | 8.31764E-06 | 0.006421 | 21.32028 |
| rs10120766 | sTrem1 | 9 | 21542208 | T | C | 0.6497 | 0.1316 | 0.0262 | 4.89779E-07 | 0.007883 | 26.21285 |
| rs11001630 | sTrem1 | 10 | 54093852 | T | C | 0.09115 | -0.2008 | 0.045 | 7.94328E-06 | 0.00668 | 22.18705 |
| rs1970171 | sTrem1 | 10 | 8745348 | A | G | 0.19146 | 0.1684 | 0.032 | 1.47911E-07 | 0.00878 | 29.22173 |
| rs1356979 | sTrem1 | 11 | 18219240 | C | T | 0.3792 | 0.1136 | 0.0252 | 6.30957E-06 | 0.006076 | 20.16674 |
| rs1044856 | sTrem1 | 13 | 43462422 | T | A | 0.35871 | 0.1292 | 0.0267 | 1.31826E-06 | 0.00768 | 25.53192 |
| rs9542197 | sTrem1 | 13 | 70741009 | G | A | 0.60757 | -0.1136 | 0.0256 | 9.12011E-06 | 0.006154 | 20.42718 |
| rs62008785 | sTrem1 | 14 | 102932572 | G | A | 0.13618 | -0.1637 | 0.0366 | 7.76247E-06 | 0.006305 | 20.93115 |
| rs113550916 | sTrem1 | 15 | 101140249 | T | C | 0.0273 | 0.4212 | 0.0777 | 6.0256E-08 | 0.009422 | 31.37921 |
| rs145950583 | sTrem1 | 18 | 67648777 | G | A | 0.01316 | 0.5111 | 0.1147 | 8.31764E-06 | 0.006785 | 22.53634 |

Chr, chromosome; EAF, Effect allele frequency; Pos, position; SE, standard error; SNP, single-nucleotide polymorphism. The threshold was set at *P* < 1×10^-5^.

**Table S5.** Characteristics of selected SNPs for neurodegenerative diseases.

| **SNP** | **Trait** | **Chr** | **Pos.** | **Effect allele** | **Other allele** | **EAF** | **Beta** | **SE** | ***P value*** | **R^2^** | **F statistic** |
| --- | --- | --- | --- | --- | --- | --- | --- | --- | --- | --- | --- |
| rs679515 | AD | 1 | 207750568 | C | T | 0.1715 | -0.1508 | 0.0183 | 1.55E-16 | 0.006462 | 415.7851 |
| rs6733839 | AD | 2 | 127892810 | T | C | 0.382 | 0.1693 | 0.0154 | 4.02E-28 | 0.013533 | 876.9547 |
| rs114812713 | AD | 6 | 41034000 | C | G | 0.0189 | 0.298 | 0.0431 | 4.47E-12 | 0.003293 | 211.2196 |
| rs34665982 | AD | 6 | 32560306 | C | T | 0.3012 | -0.0967 | 0.0166 | 5.8E-09 | 0.003936 | 252.6200 |
| rs9381563 | AD | 6 | 47432637 | T | C | 0.6443 | -0.0821 | 0.0148 | 2.93E-08 | 0.00309 | 198.1053 |
| rs11767557 | AD | 7 | 143109139 | C | T | 0.8033 | -0.1028 | 0.0182 | 1.56E-08 | 0.00334 | 214.1978 |
| rs867230 | AD | 8 | 27468503 | A | C | 0.6065 | 0.1333 | 0.0158 | 3.49E-17 | 0.008481 | 546.8005 |
| rs73223431 | AD | 8 | 27219987 | T | C | 0.3665 | 0.0936 | 0.0153 | 8.34E-10 | 0.004068 | 261.1179 |
| rs11257242 | AD | 10 | 11721119 | G | C | 0.3673 | 0.0841 | 0.0154 | 4.64E-08 | 0.003287 | 210.8311 |
| rs1582763 | AD | 11 | 60021948 | A | G | 0.3511 | -0.1232 | 0.0149 | 1.19E-16 | 0.006916 | 445.1824 |
| rs3851179 | AD | 11 | 85868640 | C | T | 0.3607 | 0.1198 | 0.0148 | 5.81E-16 | 0.006619 | 425.9343 |
| rs3740688 | AD | 11 | 47380340 | T | G | 0.5532 | 0.0935 | 0.0144 | 9.7E-11 | 0.004322 | 277.4556 |
| rs12590654 | AD | 14 | 92938855 | A | G | 0.3365 | -0.0906 | 0.0157 | 8.73E-09 | 0.003665 | 235.1641 |
| rs72654445 | AD | 19 | 45417200 | A | G | 0.0104 | -0.5425 | 0.0811 | 2.27E-11 | 0.006058 | 389.6058 |
| rs7412 | AD | 19 | 45412079 | T | C | 0.0721 | -0.4673 | 0.0305 | 6.4E-53 | 0.029219 | 1923.98 |
| rs1081105 | AD | 19 | 45412955 | C | A | 0.9739 | 0.942 | 0.0436 | 1.5E-103 | 0.045111 | 3019.937 |
| rs12151021 | AD | 19 | 1050874 | G | A | 0.3192 | -0.1071 | 0.0169 | 2.56E-10 | 0.004985 | 320.277 |
| rs111278137 | AD | 19 | 45215081 | A | G | 0.0211 | -0.4735 | 0.0713 | 3.2E-11 | 0.009262 | 597.5796 |
| rs147711004 | AD | 19 | 45337918 | A | G | 0.028 | 1.1354 | 0.0366 | 1E-200 | 0.07017 | 4824.058 |
| rs139136389 | AD | 19 | 45427136 | T | C | 0.0197 | -0.4938 | 0.0851 | 6.43E-09 | 0.009418 | 607.7583 |
| rs150685845 | AD | 19 | 45675180 | G | A | 0.9875 | 0.5561 | 0.0645 | 6.62E-18 | 0.007635 | 491.7849 |
| rs35749011 | PD | 1 | 155135036 | A | G | 0.0191 | 0.7508 | 0.0659 | 5.02E-30 | 0.021122 | 10416.23 |
| rs823106 | PD | 1 | 205656453 | C | G | 0.8488 | -0.1492 | 0.0239 | 4.1E-10 | 0.005714 | 2774.06 |
| rs4613239 | PD | 2 | 169119609 | G | C | 0.1326 | 0.1784 | 0.0248 | 6.21E-13 | 0.007321 | 3560.216 |
| rs6741007 | PD | 2 | 135537119 | G | T | 0.4507 | -0.1233 | 0.0175 | 2.09E-12 | 0.007528 | 3661.317 |
| rs4488803 | PD | 3 | 58218352 | A | G | 0.3746 | -0.1136 | 0.0199 | 1.08E-08 | 0.006047 | 2936.627 |
| rs10513789 | PD | 3 | 182760073 | G | T | 0.1826 | -0.1596 | 0.0219 | 3.18E-13 | 0.007604 | 3698.695 |
| rs7695720 | PD | 4 | 77183300 | C | A | 0.2091 | -0.1255 | 0.0208 | 1.53E-09 | 0.005209 | 2527.923 |
| rs34311866 | PD | 4 | 951947 | C | T | 0.1958 | 0.2272 | 0.0231 | 7.97E-23 | 0.016256 | 7977.080 |
| rs4698412 | PD | 4 | 15737348 | A | G | 0.553 | 0.1258 | 0.0168 | 7.05E-14 | 0.007824 | 3806.604 |
| rs356203 | PD | 4 | 90666041 | T | C | 0.6169 | -0.2398 | 0.0178 | 3.01E-41 | 0.027180 | 13487.31 |
| rs75646569 | PD | 5 | 60345424 | G | T | 0.1117 | 0.1916 | 0.0266 | 5.62E-13 | 0.007285 | 3542.516 |
| rs35265698 | PD | 6 | 32561334 | G | C | 0.1547 | -0.2000 | 0.0303 | 3.93E-11 | 0.010461 | 5103.416 |
| rs858295 | PD | 7 | 23245569 | G | A | 0.3947 | -0.1039 | 0.0176 | 3.83E-09 | 0.005158 | 2502.922 |
| rs620490 | PD | 8 | 16697579 | G | T | 0.2762 | -0.1174 | 0.019 | 6.46E-10 | 0.005511 | 2674.920 |
| rs144814361 | PD | 10 | 121410917 | T | C | 0.0174 | 0.4411 | 0.068 | 9.07E-11 | 0.006653 | 3233.194 |
| rs329647 | PD | 11 | 133764666 | C | G | 0.6662 | -0.1133 | 0.0178 | 1.94E-10 | 0.005709 | 2771.851 |
| rs75505347 | PD | 12 | 40885549 | T | C | 0.0195 | 0.3917 | 0.0674 | 6.12E-09 | 0.005867 | 2848.901 |
| rs10847864 | PD | 12 | 123326598 | T | G | 0.3625 | 0.1274 | 0.0179 | 9.81E-13 | 0.007502 | 3648.629 |
| rs4774417 | PD | 15 | 61993702 | A | G | 0.7397 | 0.1052 | 0.0192 | 4.63E-08 | 0.004262 | 2066.087 |
| rs12934900 | PD | 16 | 30923602 | T | A | 0.6571 | 0.1215 | 0.0184 | 4.33E-11 | 0.006652 | 3232.829 |
| rs58879558 | PD | 17 | 44095467 | C | T | 0.2229 | -0.2383 | 0.025 | 1.36E-21 | 0.019673 | 9687.160 |
| rs10451230 | PD | 17 | 16035225 | T | A | 0.565 | -0.096 | 0.0175 | 4.42E-08 | 0.004530 | 2196.770 |
| rs4588066 | PD | 18 | 40672964 | A | G | 0.326 | 0.1046 | 0.0178 | 4.45E-09 | 0.004808 | 2332.204 |
| rs10463311 | ALS | 5 | 150410835 | T | C | 0.744 | 0.0156 | -0.0854 | 3.99E-08 | 0.002777 | 224.509 |
| rs3849943 | ALS | 9 | 27543382 | T | C | 0.752 | 0.0155 | -0.1764 | 3.77E-30 | 0.011612 | 947.070 |
| rs142321490 | ALS | 12 | 58676132 | C | G | 0.018 | 0.0513 | 0.3172 | 6.15E-10 | 0.003615 | 292.467 |
| rs74654358 | ALS | 12 | 64881967 | A | G | 0.047 | 0.0337 | 0.1976 | 4.66E-09 | 0.003519 | 284.662 |
| rs12973192 | ALS | 19 | 17753239 | G | C | 0.325 | 0.0153 | 0.1205 | 3.92E-15 | 0.006368 | 516.578 |
| rs75087725 | ALS | 21 | 45753117 | A | C | 0.015 | 0.0672 | 0.5145 | 1.85E-14 | 0.007976 | 648.115 |

AD, Alzheimer’s Disease; ALS, amyotrophic lateral sclerosis; Chr, chromosome; EAF, Effect allele frequency; PD, Parkinson’s Disease; Pos, position; SE, standard error; SNP, single nucleotide polymorphism.

**Table S6.** Characteristics of selected SNPs for multiple sclerosis.

| **SNP** | **Trait** | **Chr** | **Pos.** | **Effect allele** | **Other allele** | **EAF** | **Beta** | **SE** | ***P value*** | **R^2^** | **F statistic** |
| --- | --- | --- | --- | --- | --- | --- | --- | --- | --- | --- | --- |
| rs10063294 | MS | 5 | 35877505 | A | G | 0.52945 | -0.09905 | 0.016264 | 1.13E-09 | 0.004888 | 568.834 |
| rs1014486 | MS | 3 | 159691112 | C | T | 0.41914 | 0.10508 | 0.016368 | 1.36E-10 | 0.005377 | 625.9711 |
| rs1026916 | MS | 17 | 40529835 | G | A | 0.65208 | -0.12965 | 0.017431 | 1.02E-13 | 0.007627 | 890.047 |
| rs1077667 | MS | 19 | 6668972 | T | C | 0.20694 | -0.15186 | 0.021225 | 8.37E-13 | 0.00757 | 883.265 |
| rs10801908 | MS | 1 | 117090493 | T | C | 0.11981 | -0.21495 | 0.026363 | 3.54E-16 | 0.009745 | 1139.566 |
| rs1112718 | MS | 10 | 94479107 | G | A | 0.4069 | -0.10562 | 0.016688 | 2.46E-10 | 0.005384 | 626.8953 |
| rs11256593 | MS | 10 | 6117322 | T | C | 0.52953 | 0.186314 | 0.017352 | 6.78E-27 | 0.017296 | 2038.135 |
| rs114872782 | MS | 6 | 32241452 | T | C | 0.01429 | -2.45044 | 0.255159 | 7.72E-22 | 0.169161 | 23577.34 |
| rs11666263 | MS | 19 | 10590684 | G | A | 0.36364 | -0.10283 | 0.018039 | 1.2E-08 | 0.004893 | 569.4579 |
| rs11749040 | MS | 5 | 40396425 | A | G | 0.1284 | 0.196745 | 0.023346 | 3.54E-17 | 0.008664 | 1012.071 |
| rs1177228 | MS | 2 | 61242410 | G | A | 0.72224 | 0.107418 | 0.018659 | 8.57E-09 | 0.00463 | 538.5957 |
| rs11809700 | MS | 1 | 93152635 | T | C | 0.26821 | 0.144448 | 0.018351 | 3.51E-15 | 0.008191 | 956.3097 |
| rs12147246 | MS | 14 | 103265844 | G | A | 0.65446 | -0.09938 | 0.016923 | 4.29E-09 | 0.004467 | 519.5795 |
| rs12365699 | MS | 11 | 118743286 | A | G | 0.15944 | -0.14375 | 0.022849 | 3.15E-10 | 0.005539 | 645.0011 |
| rs12434551 | MS | 14 | 69253364 | T | A | 0.45554 | -0.10391 | 0.016299 | 1.83E-10 | 0.005356 | 623.553 |
| rs12478539 | MS | 2 | 43355324 | C | G | 0.27942 | -0.12319 | 0.018691 | 4.37E-11 | 0.006111 | 712.0364 |
| rs1250551 | MS | 10 | 81059335 | T | G | 0.3656 | 0.115748 | 0.017369 | 2.66E-11 | 0.006215 | 724.1793 |
| rs12612620 | MS | 2 | 112488876 | A | G | 0.23258 | 0.211833 | 0.036063 | 4.26E-09 | 0.016019 | 1885.159 |
| rs12925972 | MS | 16 | 79111297 | C | T | 0.54711 | 0.094583 | 0.017081 | 3.07E-08 | 0.004433 | 515.658 |
| rs13327021 | MS | 3 | 27783015 | T | C | 0.35028 | 0.115186 | 0.017122 | 1.73E-11 | 0.006039 | 703.5808 |
| rs140522 | MS | 22 | 50971266 | C | T | 0.67141 | -0.1106 | 0.017536 | 2.85E-10 | 0.005397 | 628.3672 |
| rs1465697 | MS | 19 | 49837246 | T | C | 0.25459 | 0.124317 | 0.018766 | 3.48E-11 | 0.005866 | 683.274 |
| rs17124032 | MS | 14 | 88546009 | A | G | 0.07122 | -0.2168 | 0.031622 | 7.08E-12 | 0.006218 | 724.5973 |
| rs1738074 | MS | 6 | 159465977 | C | T | 0.58157 | 0.113729 | 0.016706 | 9.91E-12 | 0.006295 | 733.5878 |
| rs1860545 | MS | 12 | 6446777 | A | G | 0.39238 | 0.116534 | 0.017031 | 7.79E-12 | 0.006476 | 754.7584 |
| rs2150879 | MS | 17 | 57859210 | A | G | 0.55875 | -0.10355 | 0.016477 | 3.29E-10 | 0.005287 | 615.515 |
| rs2248461 | MS | 20 | 52792202 | A | G | 0.36959 | -0.10814 | 0.017417 | 5.33E-10 | 0.00545 | 634.5233 |
| rs2317231 | MS | 1 | 157686337 | T | G | 0.45005 | -0.10057 | 0.016745 | 1.9E-09 | 0.005007 | 582.6857 |
| rs2546890 | MS | 5 | 158759900 | G | A | 0.47513 | -0.11698 | 0.016418 | 1.04E-12 | 0.006826 | 795.8413 |
| rs2681424 | MS | 3 | 121769522 | C | T | 0.50438 | -0.12116 | 0.016578 | 2.71E-13 | 0.007339 | 856.112 |
| rs2857700 | MS | 6 | 31572481 | C | T | 0.86719 | -0.76722 | 0.02375 | 1E-200 | 0.135587 | 18163.96 |
| rs28703878 | MS | 8 | 79417222 | G | A | 0.27734 | 0.133646 | 0.021434 | 4.51E-10 | 0.00716 | 835.067 |
| rs34695601 | MS | 14 | 76014298 | C | T | 0.26362 | -0.10948 | 0.019791 | 3.16E-08 | 0.004654 | 541.4195 |
| rs354033 | MS | 7 | 149289464 | A | G | 0.24993 | -0.10796 | 0.018943 | 1.21E-08 | 0.00437 | 508.2367 |
| rs35486093 | MS | 1 | 85729820 | G | A | 0.09316 | 0.179486 | 0.028064 | 1.6E-10 | 0.005443 | 633.7734 |
| rs35540610 | MS | 2 | 231121829 | C | T | 0.2458 | 0.135143 | 0.019352 | 2.88E-12 | 0.006772 | 789.4937 |
| rs35703946 | MS | 16 | 86021505 | A | G | 0.16303 | -0.17252 | 0.02874 | 1.94E-09 | 0.008123 | 948.333 |
| rs3809627 | MS | 16 | 30103160 | A | C | 0.40014 | -0.09695 | 0.01754 | 3.25E-08 | 0.004512 | 524.9009 |
| rs415759 | MS | 16 | 1066917 | C | T | 0.18611 | 0.118784 | 0.021756 | 4.76E-08 | 0.004274 | 497.1117 |
| rs4325907 | MS | 3 | 101749022 | T | C | 0.64345 | -0.09927 | 0.016831 | 3.68E-09 | 0.004522 | 525.9739 |
| rs438613 | MS | 3 | 28072086 | C | T | 0.47042 | 0.138021 | 0.016606 | 9.43E-17 | 0.009492 | 1109.665 |
| rs478093 | MS | 1 | 120255126 | G | A | 0.6835 | 0.105138 | 0.017904 | 4.3E-09 | 0.004783 | 556.4882 |
| rs4808760 | MS | 19 | 18301979 | G | C | 0.29253 | -0.13456 | 0.018612 | 4.84E-13 | 0.007494 | 874.4194 |
| rs4896153 | MS | 6 | 135833463 | A | T | 0.63104 | -0.13834 | 0.018759 | 1.65E-13 | 0.008912 | 1041.312 |
| rs4939490 | MS | 11 | 60793651 | G | C | 0.39717 | 0.136627 | 0.01741 | 4.25E-15 | 0.008939 | 1044.446 |
| rs4947255 | MS | 6 | 32207483 | T | C | 0.03455 | -0.55222 | 0.055504 | 2.54E-23 | 0.020344 | 2404.728 |
| rs55970742 | MS | 7 | 2441337 | T | C | 0.67365 | -0.10037 | 0.017899 | 2.05E-08 | 0.00443 | 515.2549 |
| rs56232455 | MS | 11 | 321235 | A | G | 0.48729 | 0.15841 | 0.028125 | 1.78E-08 | 0.012539 | 1470.438 |
| rs58546351 | MS | 6 | 32483611 | G | A | 0.00785 | -1.89446 | 0.053176 | 1E-200 | 0.055905 | 6857.161 |
| rs59655222 | MS | 1 | 200875897 | C | T | 0.29811 | -0.12319 | 0.018628 | 3.76E-11 | 0.006351 | 740.1382 |
| rs6032662 | MS | 20 | 44734310 | T | C | 0.75593 | -0.13383 | 0.018329 | 2.85E-13 | 0.006609 | 770.4271 |
| rs62420820 | MS | 6 | 137438057 | A | G | 0.23797 | 0.137237 | 0.018751 | 2.5E-13 | 0.006831 | 796.4449 |
| rs6496663 | MS | 15 | 90887584 | C | A | 0.29487 | 0.100594 | 0.018109 | 2.78E-08 | 0.004208 | 489.3478 |
| rs6670198 | MS | 1 | 2520527 | C | T | 0.34621 | -0.14503 | 0.017642 | 2.03E-16 | 0.009521 | 1113.184 |
| rs6763437 | MS | 3 | 119145390 | A | G | 0.04097 | -0.77914 | 0.132145 | 3.72E-09 | 0.047705 | 5800.974 |
| rs6990534 | MS | 8 | 128814091 | G | A | 0.70972 | 0.10714 | 0.018154 | 3.6E-09 | 0.00473 | 550.3117 |
| rs701006 | MS | 12 | 58106836 | G | A | 0.60021 | 0.113864 | 0.016836 | 1.35E-11 | 0.006222 | 725.0384 |
| rs7190580 | MS | 16 | 11403470 | G | A | 0.71089 | -0.09803 | 0.01794 | 4.64E-08 | 0.00395 | 459.2801 |
| rs7200146 | MS | 16 | 11213449 | T | G | 0.6574 | -0.17079 | 0.016949 | 7E-24 | 0.013139 | 1541.765 |
| rs7207542 | MS | 17 | 45697549 | G | C | 0.49689 | 0.108765 | 0.016527 | 4.67E-11 | 0.005915 | 689.0015 |
| rs72928038 | MS | 6 | 90976768 | A | G | 0.1737 | 0.160521 | 0.024761 | 9.01E-11 | 0.007397 | 862.9146 |
| rs743771 | MS | 6 | 32976909 | A | C | 0.46633 | -0.09939 | 0.016203 | 8.55E-10 | 0.004917 | 572.2136 |
| rs74449127 | MS | 1 | 101290432 | G | A | 0.29214 | -0.19696 | 0.02558 | 1.36E-14 | 0.016045 | 1888.336 |
| rs7592560 | MS | 2 | 68647001 | A | G | 0.54881 | 0.10409 | 0.016508 | 2.87E-10 | 0.005366 | 624.7099 |
| rs7855251 | MS | 9 | 100868189 | C | T | 0.25557 | -0.11011 | 0.02009 | 4.23E-08 | 0.004613 | 536.6978 |
| rs7975763 | MS | 12 | 123604053 | T | C | 0.21037 | 0.121038 | 0.020968 | 7.8E-09 | 0.004867 | 566.3856 |
| rs9277647 | MS | 6 | 33083750 | T | C | 0.18951 | -0.21381 | 0.021168 | 5.47E-24 | 0.014043 | 1649.409 |
| rs9393975 | MS | 6 | 29802217 | A | T | 0.1769 | -0.76635 | 0.043155 | 1.49E-70 | 0.171027 | 23891.21 |
| rs9591325 | MS | 13 | 50811220 | C | T | 0.06771 | -0.21237 | 0.033989 | 4.16E-10 | 0.005694 | 663.1259 |
| rs9610458 | MS | 22 | 22205353 | T | C | 0.52202 | 0.114221 | 0.01651 | 4.57E-12 | 0.006511 | 758.8708 |
| rs9955954 | MS | 18 | 56348044 | G | A | 0.23618 | -0.11004 | 0.019451 | 1.54E-08 | 0.004369 | 508.1165 |
| rs9992763 | MS | 4 | 109058718 | T | G | 0.56754 | -0.09003 | 0.016461 | 4.51E-08 | 0.003979 | 462.6263 |

Chr, chromosome; EAF, Effect allele frequency; MS, multiple sclerosis; Pos, position; SE, standard error; SNP, single nucleotide polymorphism.

**Table S7.** Characteristics of selected SNPs for epilepsy.

| **SNP** | **Trait** | **Chr** | **Pos.** | **Effect allele** | **Other allele** | **EAF** | **Beta** | **SE** | ***P value*** | **R^2^** | **F statistic** |
| --- | --- | --- | --- | --- | --- | --- | --- | --- | --- | --- | --- |
| rs6432877 | epilepsy | 2 | 166998767 | G | C | 0.255 | 0.063437 | 0.008608 | 1.7E-13 | 0.001529 | 68.73886 |
| rs4671319 | epilepsy | 2 | 57950346 | A | G | 0.5415 | -0.04188 | 0.007262 | 8.07E-09 | 0.000871 | 39.12265 |
| rs4638568 | epilepsy | 16 | 50045839 | A | G | 0.0605 | -0.0861 | 0.015681 | 4E-08 | 0.000843 | 37.86146 |
| rs2212656 | focal epilepsy | 2 | 167000843 | A | C | 0.2504 | 0.058726 | 0.010153 | 7.3E-09 | 0.001295 | 51.00544 |
| rs11890028 | generalized epilepsy | 2 | 166943277 | G | T | 0.2789 | -0.07566 | 0.013849 | 4.68E-08 | 0.002302 | 77.18214 |
| rs887696 | generalized epilepsy | 2 | 191583507 | T | C | 0.6662 | -0.07268 | 0.013115 | 3E-08 | 0.002349 | 78.75855 |
| rs4665630 | generalized epilepsy | 2 | 23898317 | T | C | 0.8886 | -0.10899 | 0.019895 | 4.3E-08 | 0.002352 | 78.83365 |
| rs1402398 | generalized epilepsy | 2 | 58042241 | A | G | 0.6247 | -0.08662 | 0.012766 | 1.16E-11 | 0.003518 | 118.0732 |
| rs1044352 | generalized epilepsy | 4 | 31147874 | T | G | 0.4213 | 0.075176 | 0.012563 | 2.17E-09 | 0.002756 | 92.41735 |
| rs11943905 | generalized epilepsy | 4 | 46397617 | T | C | 0.2736 | 0.077543 | 0.014112 | 3.9E-08 | 0.00239 | 80.12519 |
| rs10060382 | generalized epilepsy | 5 | 114401519 | T | C | 0.4968 | -0.07253 | 0.01255 | 7.5E-09 | 0.00263 | 88.18907 |
| rs13200150 | generalized epilepsy | 6 | 128309768 | G | A | 0.3076 | -0.07818 | 0.013435 | 5.92E-09 | 0.002603 | 87.2901 |
| rs68082256 | generalized epilepsy | 6 | 16971575 | A | G | 0.2059 | -0.0934 | 0.015505 | 1.7E-09 | 0.002853 | 95.68689 |
| rs4794333 | generalized epilepsy | 17 | 46045495 | C | T | 0.3943 | -0.07359 | 0.012697 | 6.81E-09 | 0.002587 | 86.73844 |
| rs2833098 | generalized epilepsy | 21 | 32183996 | A | G | 0.6299 | 0.073068 | 0.012955 | 1.7E-08 | 0.002489 | 83.4595 |

Chr, chromosome; EAF, Effect allele frequency; Pos, position; SE, standard error; SNP, single nucleotide polymorphism.

**Table S8.** Characteristics of selected SNPs for Cerebrovascular diseases.

| **SNP** | **Trait** | **Chr** | **Pos.** | **Effect allele** | **Other allele** | **EAF** | **Beta** | **SE** | ***P value*** | **R^2^** | **F statistic** |
| --- | --- | --- | --- | --- | --- | --- | --- | --- | --- | --- | --- |
| rs2758612 | Ischemic stroke | 1 | 156205301 | C | T | 0.3547 | -0.0653 | 0.0111 | 3.68E-09 | 0.001952 | 861.1962 |
| rs34311906 | Ischemic stroke | 4 | 113732090 | C | T | 0.4024 | 0.0649 | 0.0113 | 1.07E-08 | 0.002026 | 893.8053 |
| rs2634074 | Ischemic stroke | 4 | 111677041 | A | T | 0.7877 | -0.0941 | 0.0121 | 5.9E-15 | 0.002962 | 1307.924 |
| rs2066864 | Ischemic stroke | 4 | 155525695 | A | G | 0.2452 | 0.0634 | 0.0115 | 3.51E-08 | 0.001488 | 656.1178 |
| rs11242678 | Ischemic stroke | 6 | 1337180 | T | C | 0.255 | 0.0723 | 0.0114 | 2.7E-10 | 0.001986 | 876.2757 |
| rs2107595 | Ischemic stroke | 7 | 19049388 | A | G | 0.1673 | 0.0882 | 0.0132 | 2.33E-11 | 0.002167 | 956.4634 |
| rs473238 | Ischemic stroke | 11 | 102700360 | C | T | 0.8674 | -0.0831 | 0.0147 | 1.65E-08 | 0.001589 | 700.5833 |
| rs3184504 | Ischemic stroke | 12 | 111884608 | C | T | 0.5278 | -0.0779 | 0.0101 | 1.23E-14 | 0.003025 | 1335.95 |
| rs4942561 | Ischemic stroke | 13 | 47209347 | T | G | 0.759 | 0.0655 | 0.0116 | 1.77E-08 | 0.00157 | 692.1941 |
| rs7610618 | Ischemic stroke-LAS | 3 | 149157706 | T | C | 0.013 | 0.149 | 0.8449 | 1.44E-08 | 0.018319 | 7659.931 |
| rs2107595 | Ischemic stroke-LAS | 7 | 19049388 | A | G | 0.1677 | 0.0319 | 0.2358 | 1.44E-13 | 0.015521 | 6471.700 |
| rs10820405 | Ischemic stroke-LAS | 9 | 106010237 | A | G | 0.1847 | 0.0331 | 0.181 | 4.51E-08 | 0.009889 | 4099.593 |
| rs476762 | Ischemic stroke-LAS | 11 | 102710707 | A | T | 0.133 | 0.0353 | 0.2010 | 1.22E-08 | 0.009317 | 3860.579 |
| rs7766042 | Ischemic stroke-SVS | 6 | 1366718 | C | T | 0.1016 | 0.2129 | 0.0397 | 7.97E-08 | 0.008275 | 1652.416 |
| rs12445022 | Ischemic stroke-SVS | 16 | 87575332 | A | G | 0.3367 | 0.1301 | 0.0244 | 9.26E-08 | 0.00756 | 1508.689 |
| rs146390073 | Ischemic stroke-CES | 1 | 2.41E+08 | T | C | 0.0215 | 0.6688 | 0.1195 | 2.2E-08 | 0.01882 | 7927.581 |
| rs2466455 | Ischemic stroke-CES | 4 | 1.12E+08 | T | C | 0.7826 | -0.2992 | 0.0222 | 2.75E-41 | 0.030462 | 12985.391 |
| rs6838973 | Ischemic stroke-CES | 4 | 1.12E+08 | T | C | 0.4341 | -0.1079 | 0.0196 | 3.58E-08 | 0.00572 | 2377.723 |
| rs12932445 | Ischemic stroke-CES | 16 | 73069888 | C | T | 0.1805 | 0.1758 | 0.0245 | 6.88E-13 | 0.009143 | 3813.735 |
| rs56760526 | Nontraumatic intracranial hemorrhage | 1 | 53651529 | A | G | 0.01886 | 0.5582 | 0.1041 | 8.12E-08 | 0.011531 | 2401.549 |
| rs72846646 | Nontraumatic intracranial hemorrhage | 2 | 85904055 | T | G | 0.03096 | 0.4089 | 0.0832 | 9.02E-07 | 0.010032 | 2086.209 |
| rs986697 | Nontraumatic intracranial hemorrhage | 2 | 168438360 | C | T | 0.0843 | -0.2406 | 0.0519 | 3.57E-06 | 0.008937 | 1856.405 |
| rs1214072 | Nontraumatic intracranial hemorrhage | 2 | 124667912 | C | A | 0.5176 | -0.1237 | 0.0275 | 7.08E-06 | 0.007641 | 1585.164 |
| rs17700864 | Nontraumatic intracranial hemorrhage | 2 | 18732723 | G | A | 0.05501 | 0.3047 | 0.0622 | 9.58E-07 | 0.009653 | 2006.449 |
| rs11127227 | Nontraumatic intracranial hemorrhage | 2 | 29798825 | T | A | 0.5859 | 0.1273 | 0.0282 | 6.32E-06 | 0.007863 | 1631.609 |
| rs79757240 | Nontraumatic intracranial hemorrhage | 3 | 62945992 | C | T | 0.1249 | 0.1918 | 0.0418 | 4.43E-06 | 0.008042 | 1668.883 |
| rs62357396 | Nontraumatic intracranial hemorrhage | 5 | 28315548 | A | C | 0.2351 | -0.1518 | 0.0329 | 3.88E-06 | 0.008288 | 1720.351 |
| rs79829591 | Nontraumatic intracranial hemorrhage | 6 | 125593275 | A | G | 0.03651 | 0.3507 | 0.0746 | 2.57E-06 | 0.008653 | 1796.829 |
| rs13227082 | Nontraumatic intracranial hemorrhage | 7 | 96659653 | G | A | 0.3859 | 0.1564 | 0.0283 | 3.12E-08 | 0.011594 | 2414.648 |
| rs151160311 | Nontraumatic intracranial hemorrhage | 8 | 66816894 | A | G | 0.004986 | 0.9583 | 0.215 | 8.32E-06 | 0.009112 | 1893.049 |
| rs2673593 | Nontraumatic intracranial hemorrhage | 8 | 133444342 | G | T | 0.3706 | 0.1455 | 0.0286 | 3.69E-07 | 0.009876 | 2053.386 |
| rs549427728 | Nontraumatic intracranial hemorrhage | 9 | 33575398 | A | G | 0.02442 | 0.4328 | 0.0944 | 4.55E-06 | 0.008925 | 1853.867 |
| rs7861405 | Nontraumatic intracranial hemorrhage | 9 | 81389995 | A | G | 0.2141 | 0.1572 | 0.0337 | 3.09E-06 | 0.008316 | 1726.306 |
| rs7358643 | Nontraumatic intracranial hemorrhage | 12 | 95489131 | G | A | 0.2844 | 0.1374 | 0.0306 | 7.26E-06 | 0.007684 | 1594.136 |
| rs9527141 | Nontraumatic intracranial hemorrhage | 13 | 33725687 | T | C | 0.3169 | 0.1349 | 0.0296 | 5.3E-06 | 0.007879 | 1634.811 |
| rs143645831 | Nontraumatic intracranial hemorrhage | 16 | 76338960 | G | A | 0.007594 | 0.7592 | 0.1695 | 7.46E-06 | 0.008688 | 1804.113 |
| rs111841004 | Nontraumatic intracranial hemorrhage | 17 | 71437622 | T | C | 0.01792 | 0.4907 | 0.1074 | 4.96E-06 | 0.008475 | 1759.606 |
| rs12606544 | Nontraumatic intracranial hemorrhage | 18 | 47123237 | C | T | 0.2597 | 0.1533 | 0.0315 | 1.13E-06 | 0.009036 | 1877.19 |
| rs78756118 | Nontraumatic intracranial hemorrhage | 20 | 47417281 | G | T | 0.07288 | -0.2451 | 0.053 | 3.75E-06 | 0.008118 | 1684.896 |
| rs16838082 | Subarachnoid hemorrhage | 1 | 195140113 | G | A | 0.3588 | -0.1911 | 0.0413 | 3.7E-06 | 0.016803 | 3461.972 |
| rs10465884 | Subarachnoid hemorrhage | 1 | 147211325 | C | T | 0.03687 | 0.4841 | 0.1062 | 5.16E-06 | 0.016644 | 3428.578 |
| rs792108 | Subarachnoid hemorrhage | 2 | 5532793 | C | T | 0.5901 | -0.2011 | 0.0404 | 6.27E-07 | 0.019564 | 4042.081 |
| rs11127227 | Subarachnoid hemorrhage | 2 | 29798825 | T | A | 0.5858 | 0.1804 | 0.0406 | 8.62E-06 | 0.015793 | 3250.443 |
| rs141918148 | Subarachnoid hemorrhage | 4 | 162217380 | C | G | 0.0707 | 0.3559 | 0.0798 | 8.22E-06 | 0.016644 | 3428.603 |
| rs188736573 | Subarachnoid hemorrhage | 6 | 139515199 | T | C | 0.03705 | 0.5187 | 0.1084 | 1.71E-06 | 0.019198 | 3964.967 |
| rs13265849 | Subarachnoid hemorrhage | 8 | 39139198 | A | G | 0.3907 | -0.1812 | 0.0406 | 7.95E-06 | 0.015632 | 3216.845 |
| rs2673593 | Subarachnoid hemorrhage | 8 | 133444342 | G | T | 0.3706 | 0.1935 | 0.0411 | 2.49E-06 | 0.017467 | 3601.17 |
| rs41306063 | Subarachnoid hemorrhage | 9 | 6415643 | G | A | 0.03393 | 0.5071 | 0.1131 | 7.28E-06 | 0.016858 | 3473.442 |
| rs10759244 | Subarachnoid hemorrhage | 9 | 110341137 | C | G | 0.3584 | -0.1852 | 0.0413 | 7.44E-06 | 0.015774 | 3246.505 |
| rs2050970 | Subarachnoid hemorrhage | 10 | 515870 | T | C | 0.5734 | 0.1778 | 0.0401 | 9.2E-06 | 0.015466 | 3182.056 |
| rs2505256 | Subarachnoid hemorrhage | 10 | 38369818 | T | A | 0.3319 | 0.2504 | 0.0533 | 2.57E-06 | 0.027807 | 5793.77 |
| rs16930998 | Subarachnoid hemorrhage | 11 | 5462702 | A | G | 0.01178 | 0.9366 | 0.1976 | 2.14E-06 | 0.020424 | 4223.433 |
| rs142664188 | Subarachnoid hemorrhage | 12 | 123137721 | G | C | 0.004516 | 1.7156 | 0.3529 | 1.17E-06 | 0.026464 | 5506.361 |
| rs11609982 | Subarachnoid hemorrhage | 12 | 107709150 | A | G | 0.3344 | 0.1989 | 0.0421 | 2.35E-06 | 0.017611 | 3631.3 |
| rs187128461 | Subarachnoid hemorrhage | 13 | 74786576 | G | A | 0.01839 | 0.6817 | 0.1527 | 8.09E-06 | 0.016778 | 3456.624 |
| rs6602891 | Subarachnoid hemorrhage | 13 | 114517085 | T | G | 0.4052 | 0.1902 | 0.0405 | 2.71E-06 | 0.017438 | 3594.992 |
| rs10163011 | Subarachnoid hemorrhage | 15 | 93760024 | C | T | 0.9792 | -0.6811 | 0.1476 | 3.94E-06 | 0.018897 | 3901.56 |
| rs2738814 | Subarachnoid hemorrhage | 16 | 75516282 | A | C | 0.6363 | -0.2003 | 0.0412 | 1.13E-06 | 0.018569 | 3832.693 |
| rs117080463 | Subarachnoid hemorrhage | 17 | 46694734 | A | G | 0.004078 | 1.7269 | 0.3794 | 5.32E-06 | 0.024224 | 5028.67 |
| rs2826730 | Subarachnoid hemorrhage | 21 | 22571465 | G | C | 0.1129 | -0.2883 | 0.0636 | 5.82E-06 | 0.016649 | 3429.602 |

CES, cardioembolic; Chr, chromosome; EAF, Effect allele frequency; LAS, large artery atherosclerosis; Pos, position; SE, standard error; SNP, single nucleotide polymorphism; SVS, small-vessel. The threshold was set at *P* < 1×10^-5^ for nontraumatic intracranial hemorrhage and subarachnoid hemorrhage.

**Table S9.** Characteristics of selected SNPs for migraine.

| **SNP** | **Trait** | **Chr** | **Pos.** | **Effect allele** | **Other allele** | **EAF** | **Beta** | **SE** | ***P value*** | **R^2^** | **F statistic** |
| --- | --- | --- | --- | --- | --- | --- | --- | --- | --- | --- | --- |
| rs146240626 | Migraine with aura | 2 | 149112325 | T | C | 0.2347 | 0.132 | 0.029 | 5.4E-06 | 0.006259 | 1131.533 |
| rs4849052 | Migraine with aura | 2 | 112948678 | C | T | 0.9835 | -0.5351 | 0.1018 | 1.49E-07 | 0.009293 | 1685.119 |
| rs73928973 | Migraine with aura | 2 | 40621139 | C | A | 0.08106 | 0.2004 | 0.0447 | 7.33E-06 | 0.005983 | 1081.292 |
| rs13088480 | Migraine with aura | 3 | 157496844 | C | T | 0.4693 | 0.1187 | 0.0247 | 1.58E-06 | 0.007018 | 1269.718 |
| rs139699059 | Migraine with aura | 3 | 83150462 | C | T | 0.01138 | 0.5615 | 0.1235 | 5.5E-06 | 0.007094 | 1283.544 |
| rs3856982 | Migraine with aura | 4 | 1328863 | C | T | 0.1571 | -0.1683 | 0.0339 | 7.02E-07 | 0.007502 | 1357.807 |
| rs189638748 | Migraine with aura | 4 | 75377048 | A | G | 0.009006 | 0.6609 | 0.1436 | 4.19E-06 | 0.007797 | 1411.631 |
| rs7724203 | Migraine with aura | 5 | 61119308 | C | T | 0.1742 | 0.1436 | 0.0324 | 9.54E-06 | 0.005933 | 1072.17 |
| rs73004193 | Migraine with aura | 6 | 147042158 | G | A | 0.03346 | 0.3389 | 0.0717 | 2.29E-06 | 0.007429 | 1344.543 |
| rs62457050 | Migraine with aura | 7 | 45487985 | T | C | 0.132 | 0.1752 | 0.0376 | 3.1E-06 | 0.007034 | 1272.55 |
| rs953255 | Migraine with aura | 8 | 108540020 | C | A | 0.114 | 0.1731 | 0.0387 | 7.79E-06 | 0.006053 | 1093.999 |
| rs17271445 | Migraine with aura | 10 | 4415069 | G | T | 0.0937 | -0.1883 | 0.0423 | 8.32E-06 | 0.006022 | 1088.386 |
| rs7398375 | Migraine with aura | 12 | 57540848 | G | C | 0.3322 | 0.1174 | 0.0265 | 9.27E-06 | 0.006115 | 1105.335 |
| rs7135461 | Migraine with aura | 12 | 100037404 | A | C | 0.5373 | 0.1195 | 0.0247 | 1.36E-06 | 0.007100 | 1284.678 |
| rs142937471 | Migraine with aura | 15 | 67828241 | T | C | 0.0175 | 0.4343 | 0.0973 | 8.08E-06 | 0.006486 | 1172.8 |
| rs144755021 | Migraine with aura | 17 | 72659621 | A | G | 0.01272 | 0.5324 | 0.1192 | 8.01E-06 | 0.007119 | 1288.113 |
| rs16035 | Migraine with aura | 19 | 13356150 | G | T | 0.5029 | -0.1279 | 0.0248 | 2.45E-07 | 0.008179 | 1481.429 |
| rs382583 | Migraine with aura | 21 | 15589167 | C | T | 0.8607 | 0.1643 | 0.036 | 4.87E-06 | 0.006473 | 1170.431 |
| rs62177052 | Migraine without aura, drug-induced | 2 | 133587775 | A | C | 0.1455 | 0.6944 | 0.1546 | 7.08E-06 | 0.119901 | 29807.16 |
| rs7687061 | Migraine without aura, drug-induced | 4 | 126962120 | C | T | 0.9403 | -1.1609 | 0.2502 | 3.49E-06 | 0.151307 | 39006.54 |
| rs16902785 | Migraine without aura, drug-induced | 5 | 87048800 | A | T | 0.003542 | 5.9589 | 1.3364 | 8.24E-06 | 0.250651 | 73183.5 |
| rs185370443 | Migraine without aura, drug-induced | 5 | 133376987 | T | C | 0.008891 | 3.1176 | 0.6934 | 6.93E-06 | 0.171294 | 45224.1 |
| rs4977739 | Migraine without aura, drug-induced | 9 | 21876407 | G | A | 0.3243 | -0.5193 | 0.1127 | 4.08E-06 | 0.118186 | 29323.66 |
| rs117283049 | Migraine without aura, drug-induced | 11 | 7690627 | G | T | 0.05611 | 1.3892 | 0.2536 | 4.28E-08 | 0.204419 | 56216.56 |
| rs117847543 | Migraine without aura, drug-induced | 14 | 74651913 | G | A | 0.0163 | 2.3438 | 0.5283 | 9.16E-06 | 0.176166 | 46785.25 |
| rs12976357 | Migraine without aura, drug-induced | 19 | 18467597 | C | T | 0.6891 | -0.5145 | 0.1142 | 6.59E-06 | 0.113424 | 27990.78 |
| rs115668283 | Migraine without aura and triptan purchases | 1 | 95251044 | C | T | 0.02808 | 0.3667 | 0.0812 | 6.24E-06 | 0.00734 | 1237.096 |
| rs34583461 | Migraine without aura and triptan purchases | 1 | 2753227 | C | A | 0.01835 | 0.4778 | 0.1026 | 3.23E-06 | 0.008225 | 1387.478 |
| rs112253950 | Migraine without aura and triptan purchases | 2 | 219809241 | A | G | 0.03103 | 0.3571 | 0.0768 | 3.34E-06 | 0.007668 | 1292.913 |
| rs11681583 | Migraine without aura and triptan purchases | 2 | 39129935 | G | A | 0.314 | 0.1301 | 0.0282 | 3.98E-06 | 0.007292 | 1228.97 |
| rs75280455 | Migraine without aura and triptan purchases | 3 | 30654362 | A | C | 0.02016 | 0.4528 | 0.0956 | 2.17E-06 | 0.008100 | 1366.297 |
| rs2191685 | Migraine without aura and triptan purchases | 4 | 14392437 | C | G | 0.7119 | 0.1287 | 0.029 | 9.01E-06 | 0.006794 | 1144.550 |
| rs2431529 | Migraine without aura and triptan purchases | 5 | 102125564 | G | A | 0.3821 | -0.1274 | 0.027 | 2.35E-06 | 0.007664 | 1292.200 |
| rs6944957 | Migraine without aura and triptan purchases | 7 | 89338551 | A | G | 0.7021 | 0.1277 | 0.0286 | 7.95E-06 | 0.006822 | 1149.155 |
| rs13230396 | Migraine without aura and triptan purchases | 7 | 17712428 | A | G | 0.2273 | 0.1417 | 0.0312 | 5.58E-06 | 0.007053 | 1188.443 |
| rs9642404 | Migraine without aura and triptan purchases | 7 | 55856799 | A | G | 0.1552 | -0.1732 | 0.0359 | 1.42E-06 | 0.007866 | 1326.556 |
| rs111823172 | Migraine without aura and triptan purchases | 8 | 52014801 | T | C | 0.004813 | 1.0058 | 0.2161 | 3.27E-06 | 0.009691 | 1637.298 |
| rs111513989 | Migraine without aura and triptan purchases | 9 | 94675146 | T | C | 0.1641 | 0.1605 | 0.0353 | 5.42E-06 | 0.007067 | 1190.824 |
| rs76757807 | Migraine without aura and triptan purchases | 10 | 57405513 | G | C | 0.02848 | 0.3847 | 0.0823 | 2.91E-06 | 0.008190 | 1381.535 |
| rs1577816 | Migraine without aura and triptan purchases | 10 | 23919860 | C | G | 0.2269 | -0.139 | 0.0313 | 8.88E-06 | 0.006778 | 1141.848 |
| rs6490179 | Migraine without aura and triptan purchases | 12 | 118891189 | C | T | 0.5364 | 0.1184 | 0.0268 | 9.64E-06 | 0.006972 | 1174.705 |
| rs1155688 | Migraine without aura and triptan purchases | 12 | 128887700 | A | G | 0.7745 | -0.1498 | 0.0313 | 1.75E-06 | 0.007838 | 1321.794 |
| rs10774231 | Migraine without aura and triptan purchases | 12 | 4515374 | C | T | 0.553 | -0.1239 | 0.0263 | 2.49E-06 | 0.007589 | 1279.494 |
| rs6563653 | Migraine without aura and triptan purchases | 13 | 39524160 | G | A | 0.7681 | 0.1474 | 0.0311 | 2.17E-06 | 0.007740 | 1305.096 |
| rs61980275 | Migraine without aura and triptan purchases | 14 | 94227645 | A | G | 0.1247 | 0.1827 | 0.0402 | 5.58E-06 | 0.007287 | 1228.093 |
| rs7203655 | Migraine without aura and triptan purchases | 16 | 88826862 | T | C | 0.5352 | 0.1166 | 0.0263 | 9.07E-06 | 0.006764 | 1139.414 |
| rs71370493 | Migraine without aura and triptan purchases | 17 | 7303996 | T | C | 0.241 | 0.1398 | 0.0308 | 5.71E-06 | 0.007150 | 1204.881 |
| rs72932480 | Migraine without aura and triptan purchases | 18 | 55136254 | T | C | 0.01229 | 0.5554 | 0.1243 | 7.93E-06 | 0.007489 | 1262.444 |
| rs11087103 | Migraine without aura and triptan purchases | 20 | 14695004 | T | C | 0.1381 | 0.1704 | 0.0381 | 7.79E-06 | 0.006912 | 1164.546 |
| rs73145783 | Migraine without aura and triptan purchases | 20 | 61670038 | G | A | 0.1258 | 0.212 | 0.0396 | 8.56E-08 | 0.009885 | 1670.445 |
| rs74925396 | Migraine without aura and triptan purchases | 22 | 22124381 | G | A | 0.02806 | 0.4025 | 0.0823 | 1E-06 | 0.008837 | 1491.654 |
| rs115668283 | Migraine without aura and triptan purchases | 1 | 95251044 | C | T | 0.02808 | 0.3667 | 0.0812 | 6.24E-06 | 0.007340 | 1237.096 |

Chr, chromosome; EAF, Effect allele frequency; LAS, large artery atherosclerosis; Pos, position; SE, standard error; SNP, single nucleotide polymorphism. The threshold was set at *P* < 1×10^-5^.

**Supplementary Figures**

**Figure S1.** The causal effect of plasma sTrem1 on AD risk. (A) Scatter plot, (B) Funnel plot, (C) Forest plot, and (D) Leave one out plot. AD, Alzheimer’s Disease.

**
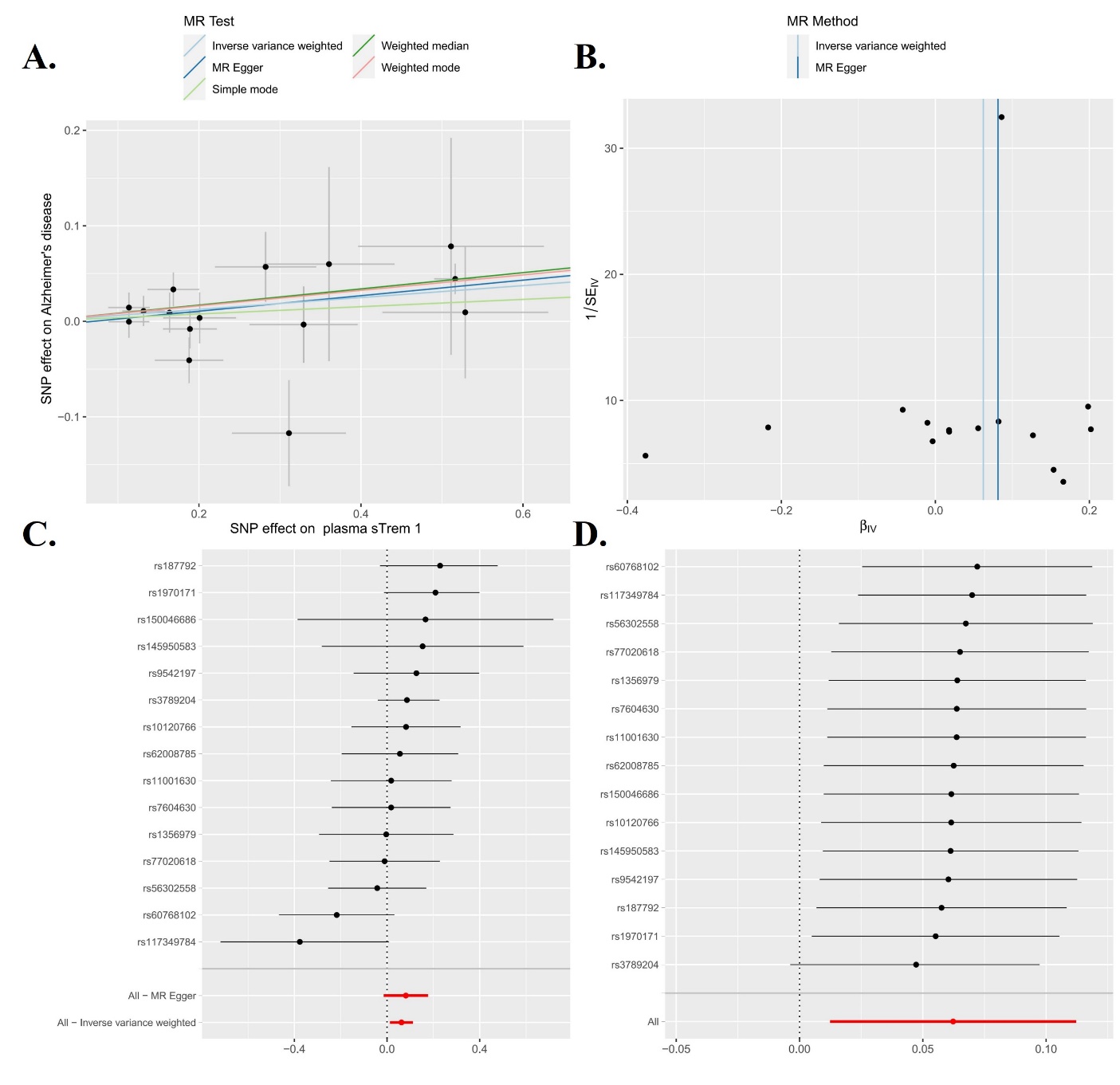
**

**Figure S2.** The causal effect of plasma sTrem1 on PD risk. (A) Scatter plot, (B) Funnel plot, (C) Forest plot, and (D) Leave one out plot. PD, Parkinson’s Disease.

**
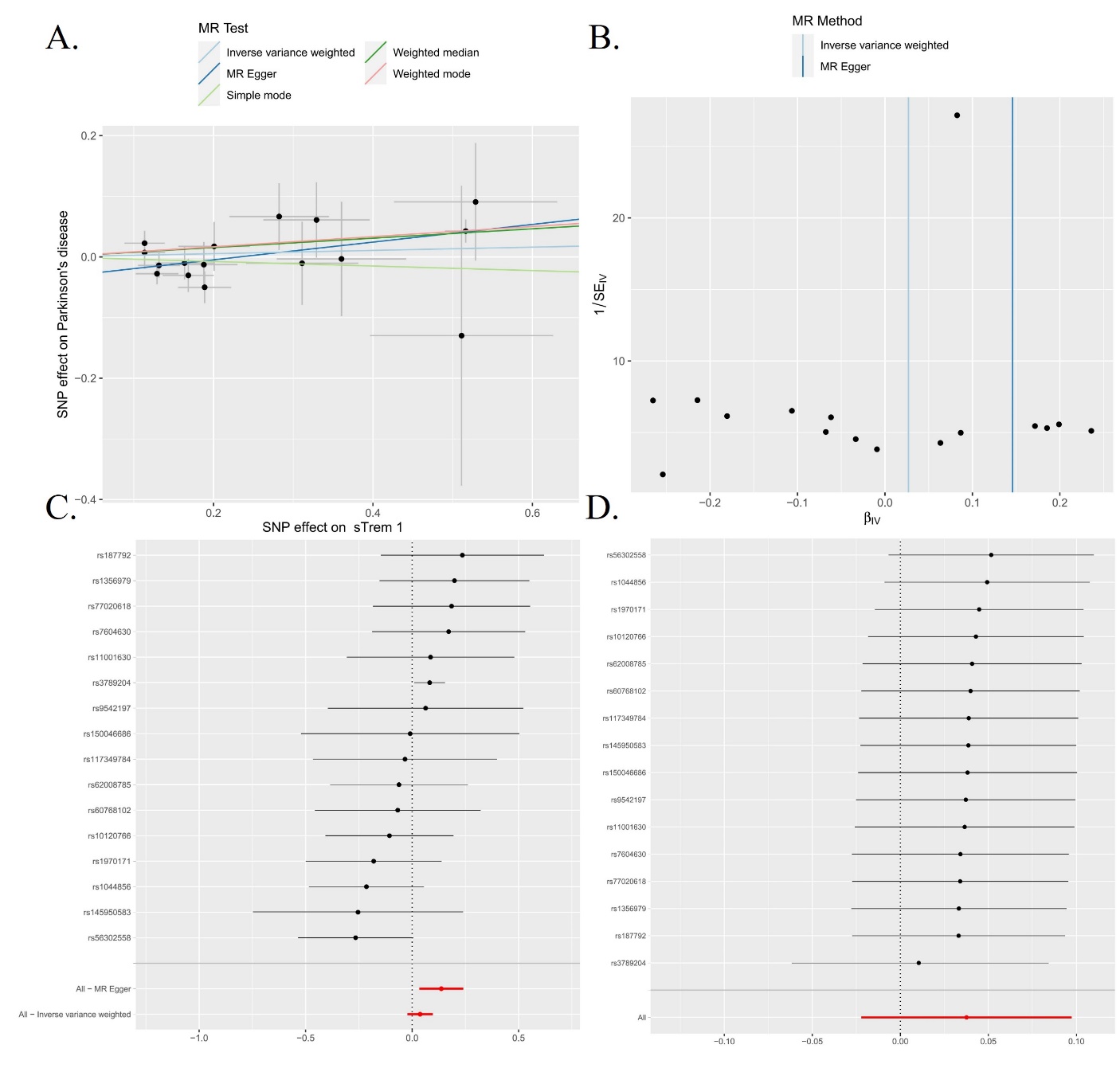
**

**Figure S3.** The causal effect of plasma sTrem1 on ALS risk. (A) Scatter plot, (B) Funnel plot, (C) Forest plot, and (D) Leave one out plot. ALS, amyotrophic lateral sclerosis.

**
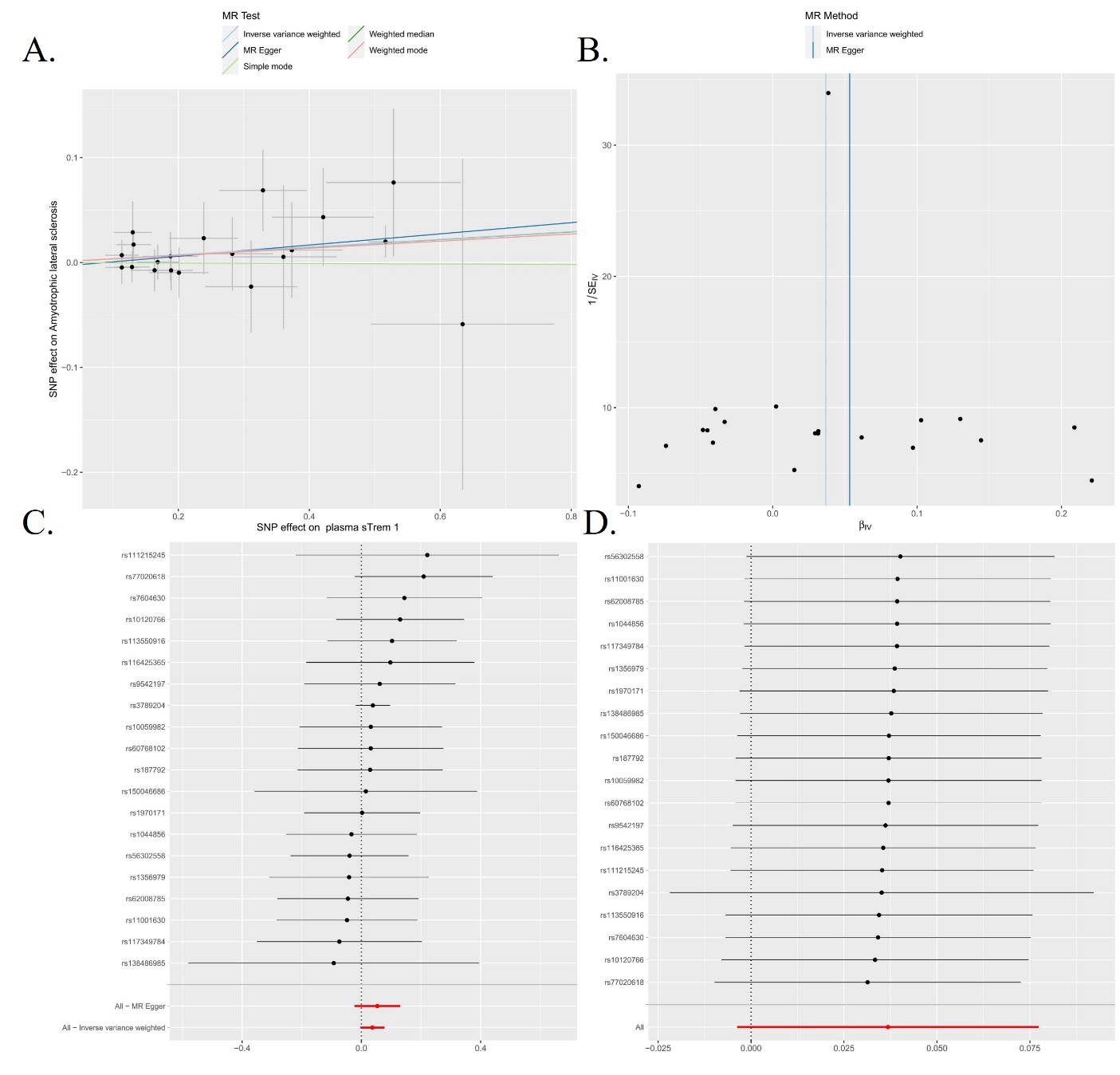
**

**Figure S4.** The causal effect of plasma sTrem1 on MS risk. (A) Scatter plot, (B) Funnel plot, (C) Forest plot, and (D) Leave one out plot. MS, multiple sclerosis.

**
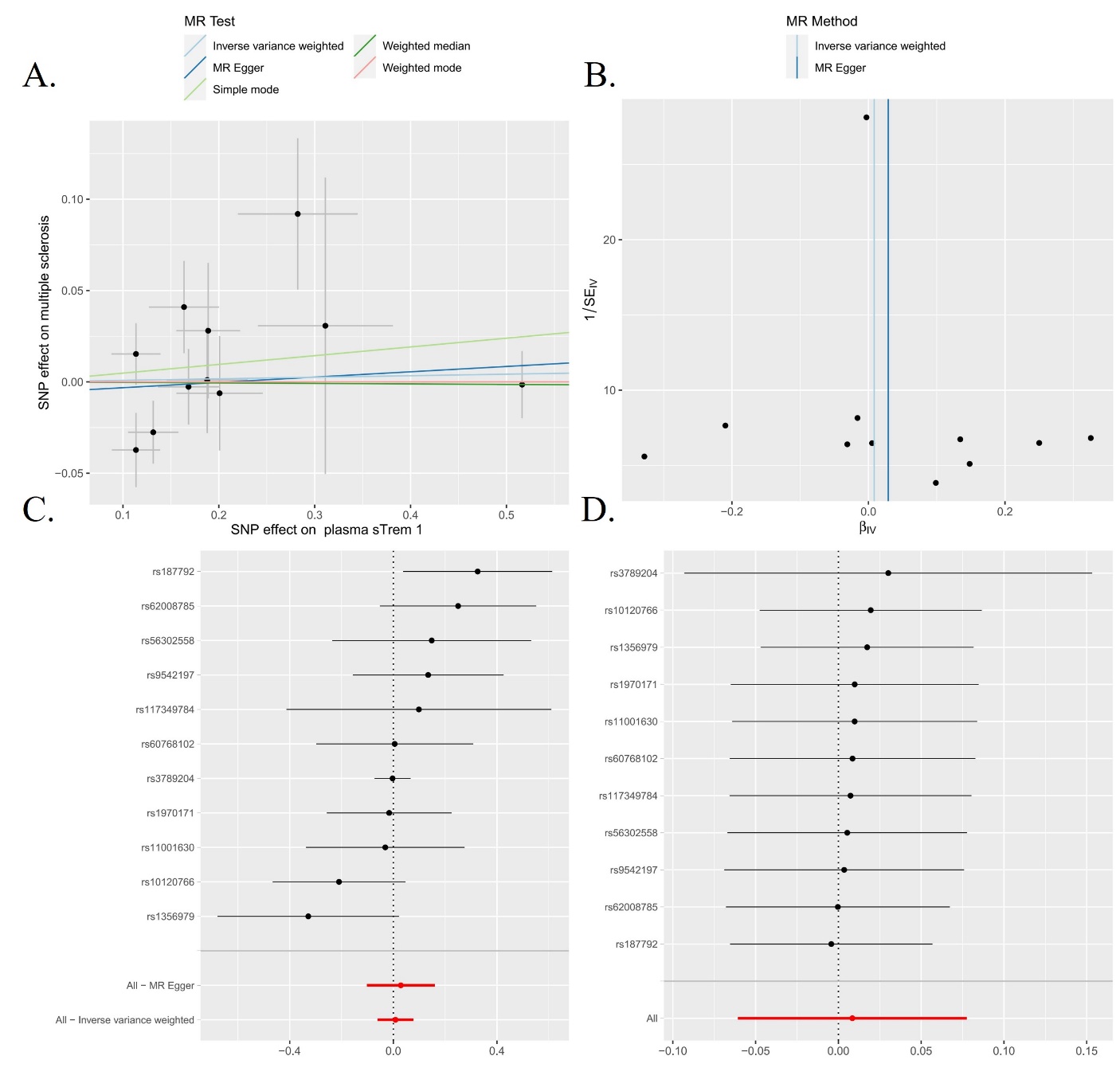
**

**Figure S5.** The causal effect of plasma sTrem1 on epilepsy risk. (A) Scatter plot, (B) Funnel plot, (C) Forest plot, and (D) Leave one out plot.

**
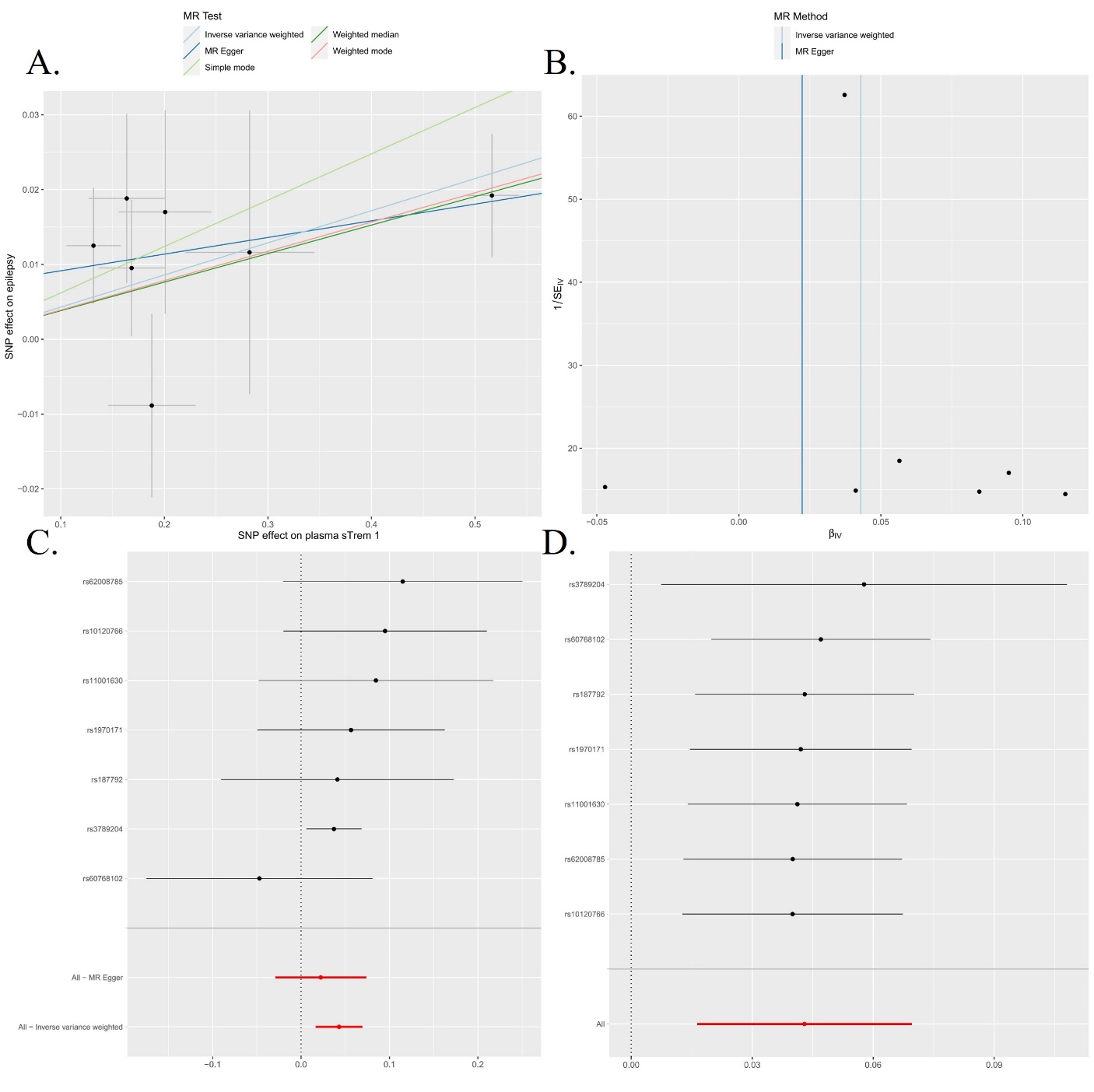
**

**Figure S6.** The causal effect of plasma sTrem1 on generalized epilepsy risk. (A) Scatter plot, (B) Funnel plot, (C) Forest plot, and (D) Leave one out plot.

**
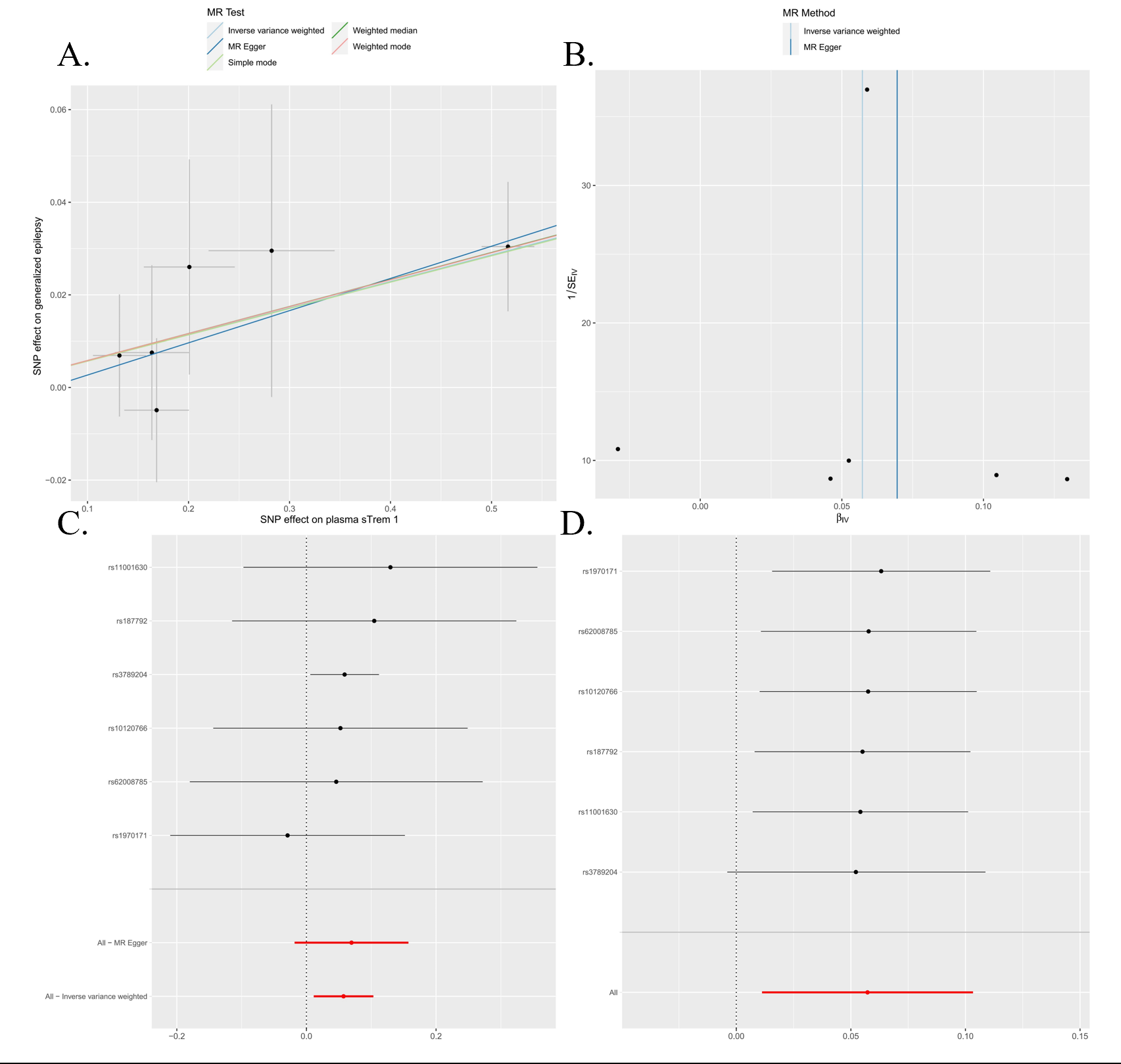
**

**Figure S7.** The causal effect of plasma sTrem1 on focal epilepsy risk. (A) Scatter plot, (B) Funnel plot, (C) Forest plot, and (D) Leave one out plot.

**
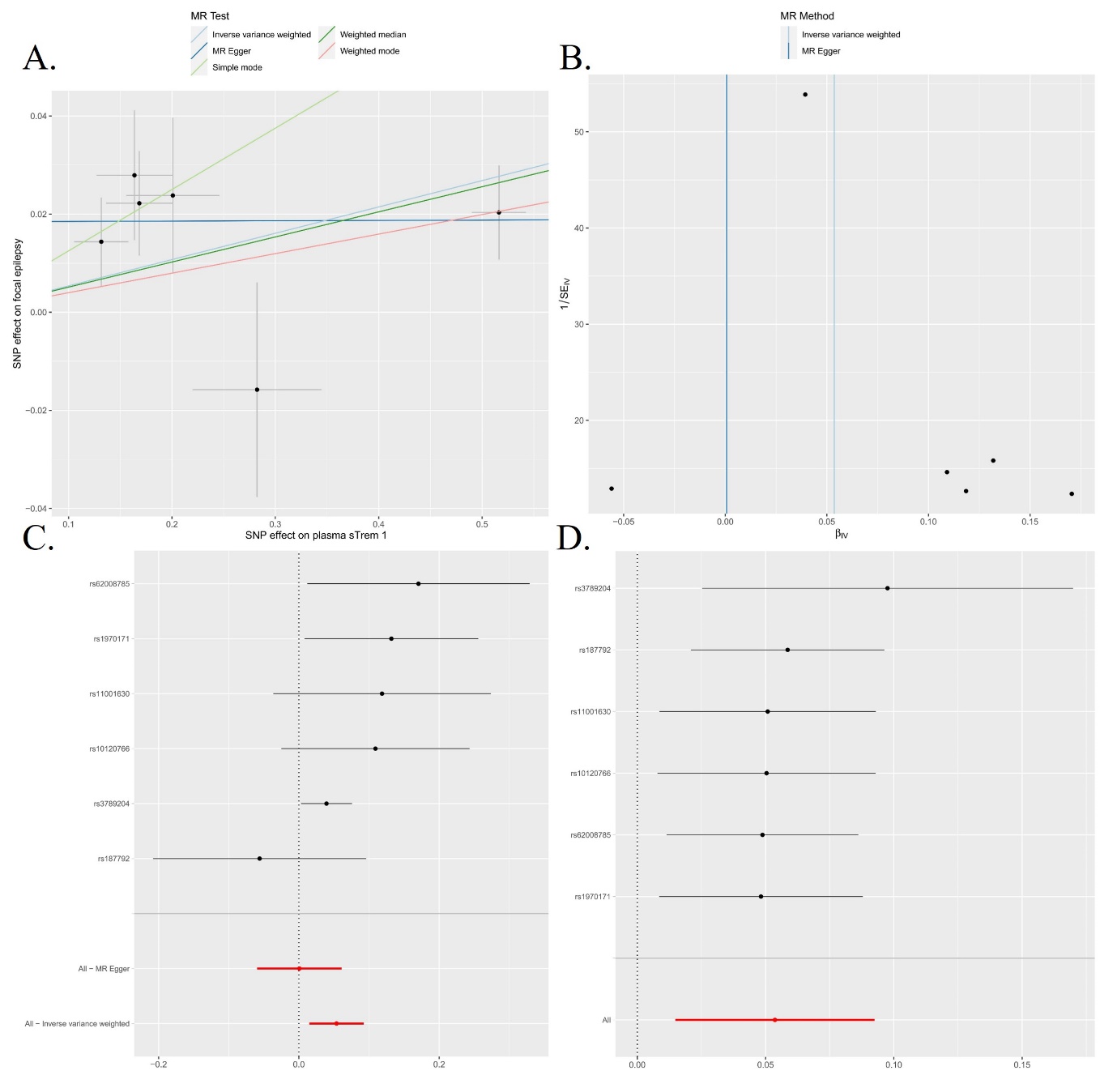
**

**Figure S8.** The causal effect of plasma sTrem1 on ischemic stroke risk. (A) Scatter plot, (B) Funnel plot, (C) Forest plot, and (D) Leave one out plot.

**
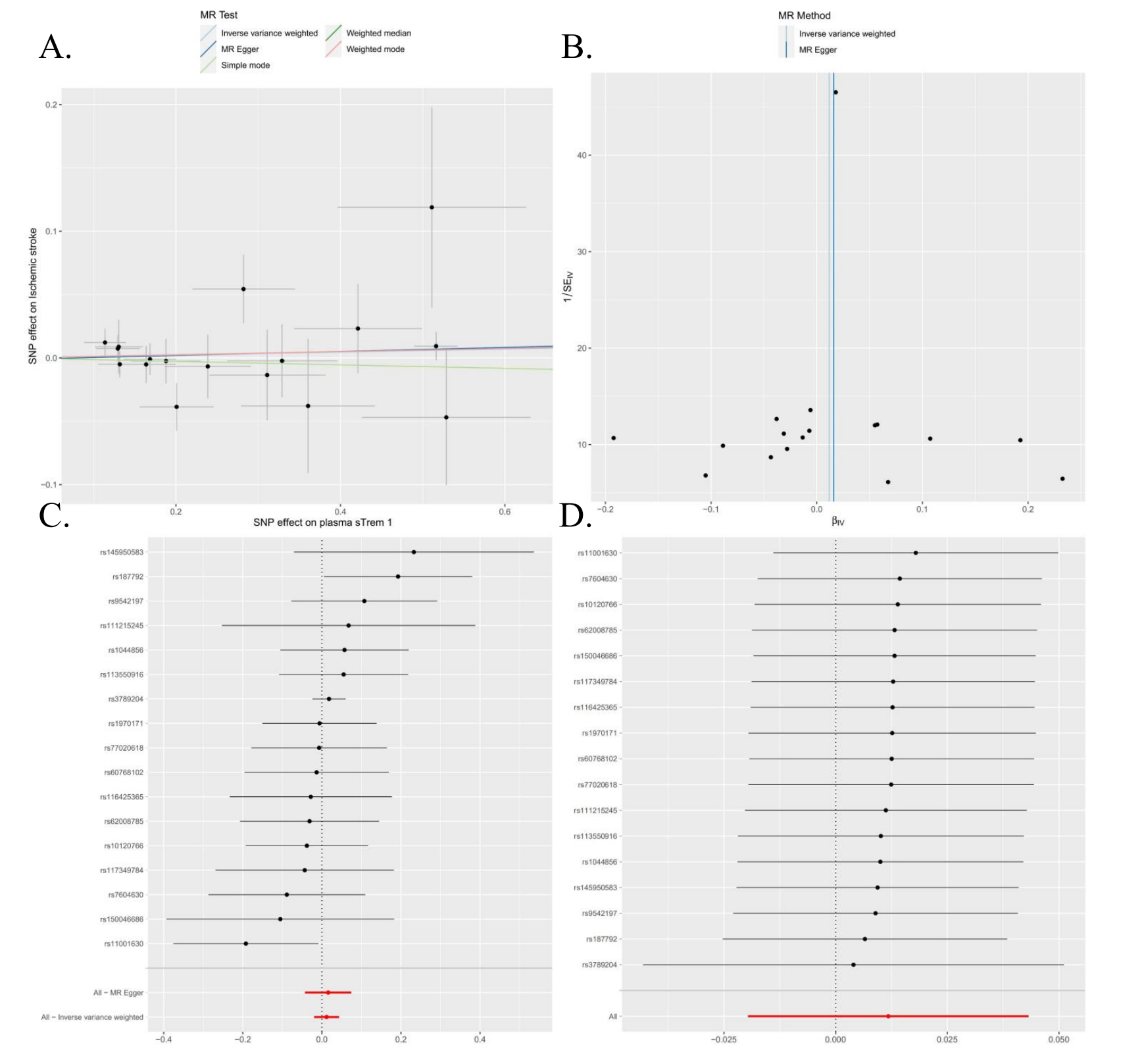
**

**Figure S9.** The causal effect of plasma sTrem1 on ischemic stroke (large artery atherosclerosis) risk. (A) Scatter plot, (B) Funnel plot, (C) Forest plot, and (D) Leave one out plot.

**
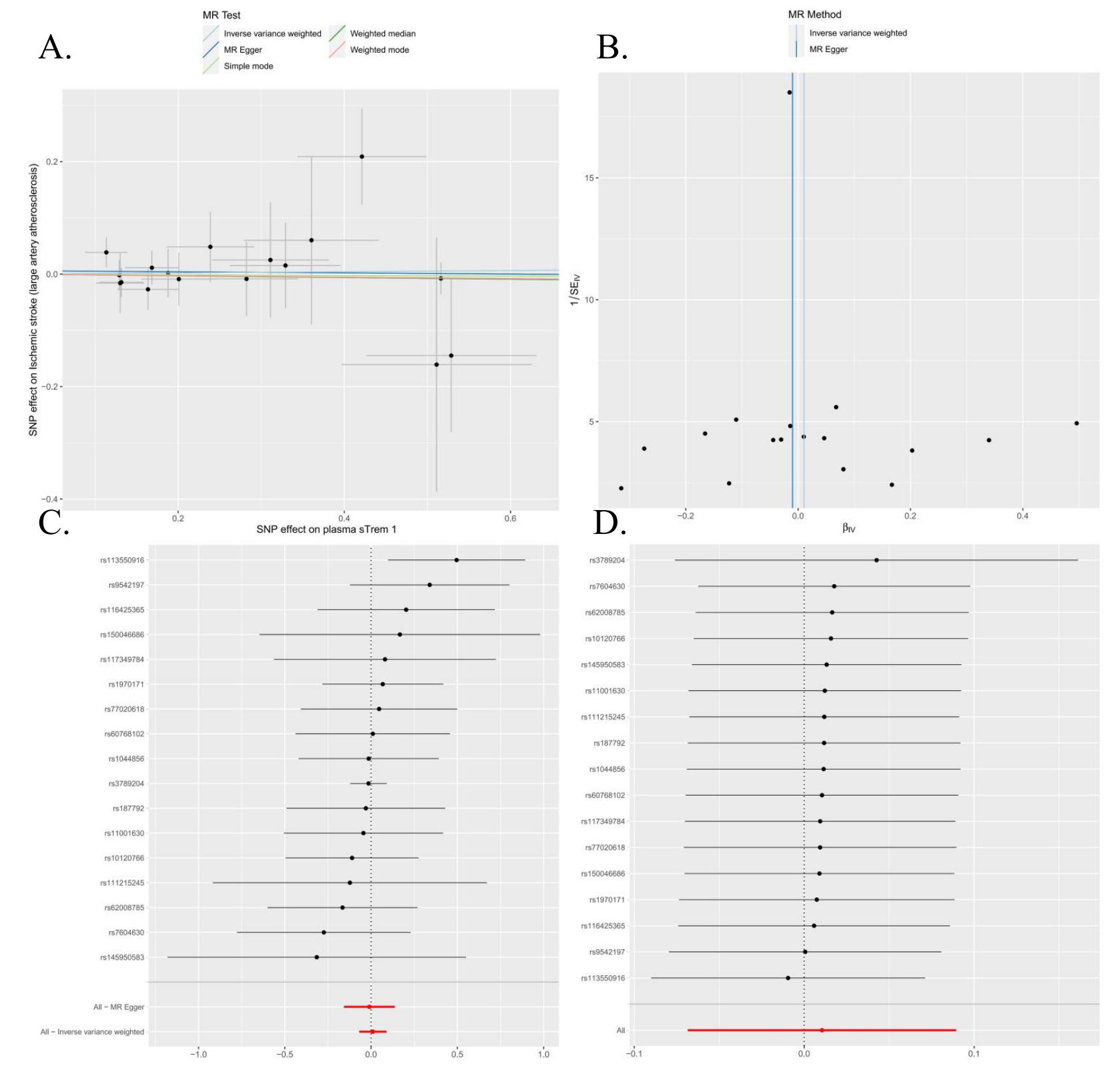
**

**Figure S10.** The causal effect of plasma sTrem1 on ischemic stroke (cardioembolic) risk. (A) Scatter plot, (B) Funnel plot, (C) Forest plot, and (D) Leave one out plot.

**
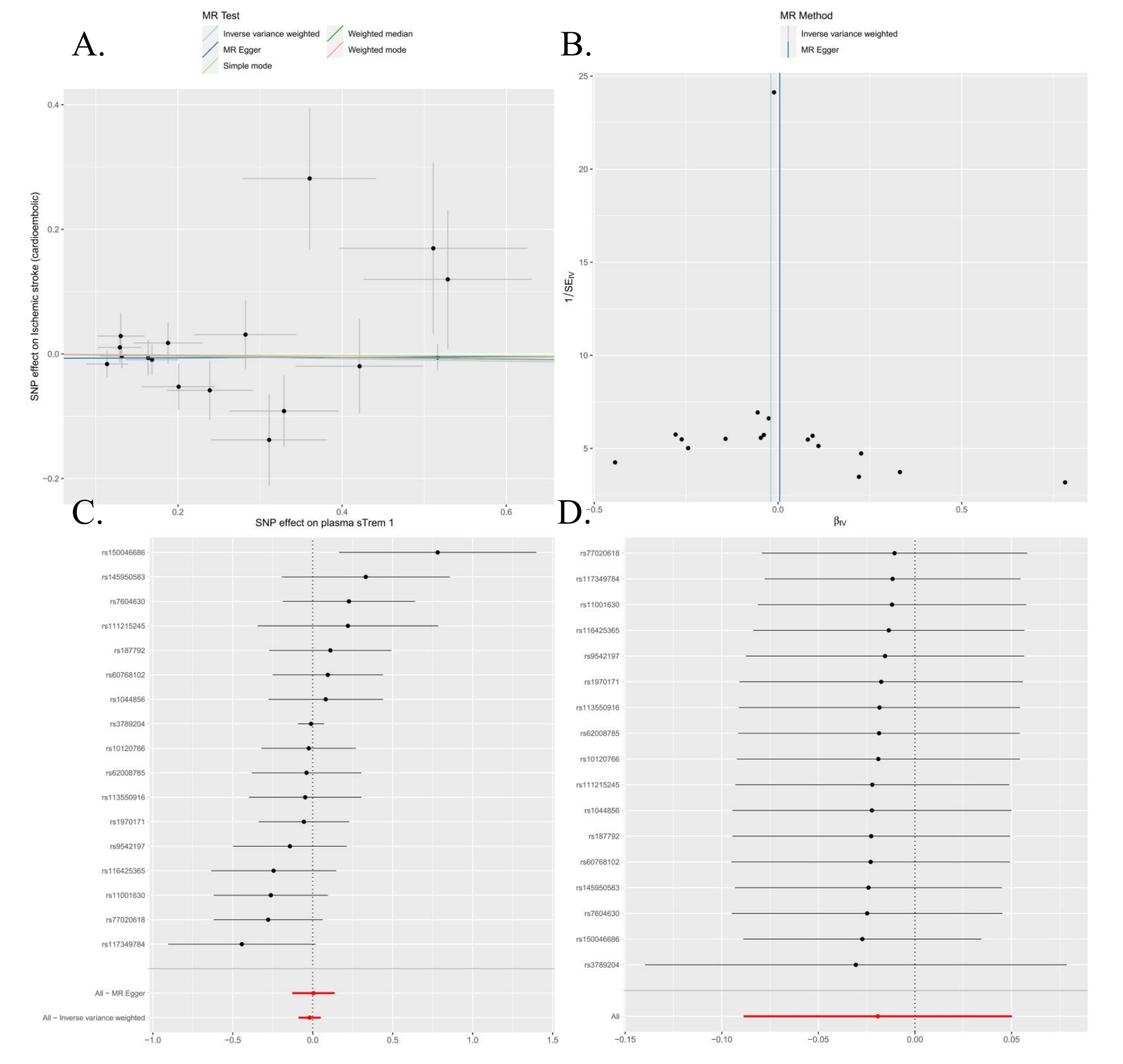
**

**Figure S11.** The causal effect of plasma sTrem1 on nontraumatic intracranial hemorrhage risk. (A) Scatter plot, (B) Funnel plot, (C) Forest plot, and (D) Leave one out plot.

**
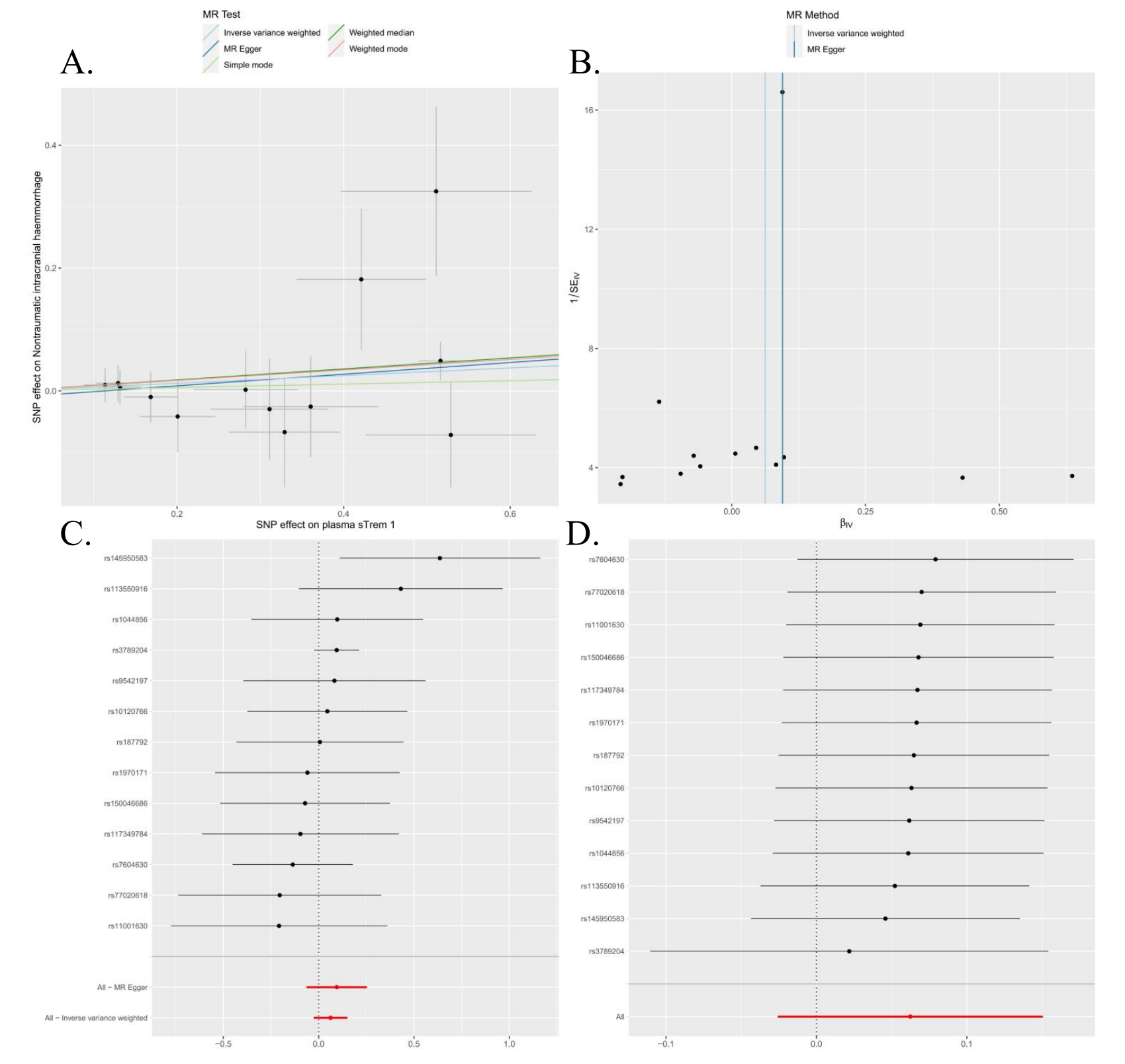
**

**Figure S12.** The causal effect of plasma sTrem1 on subarachnoid haemmorrhage risk. (A) Scatter plot, (B) Funnel plot, (C) Forest plot, and (D) Leave one out plot.

**
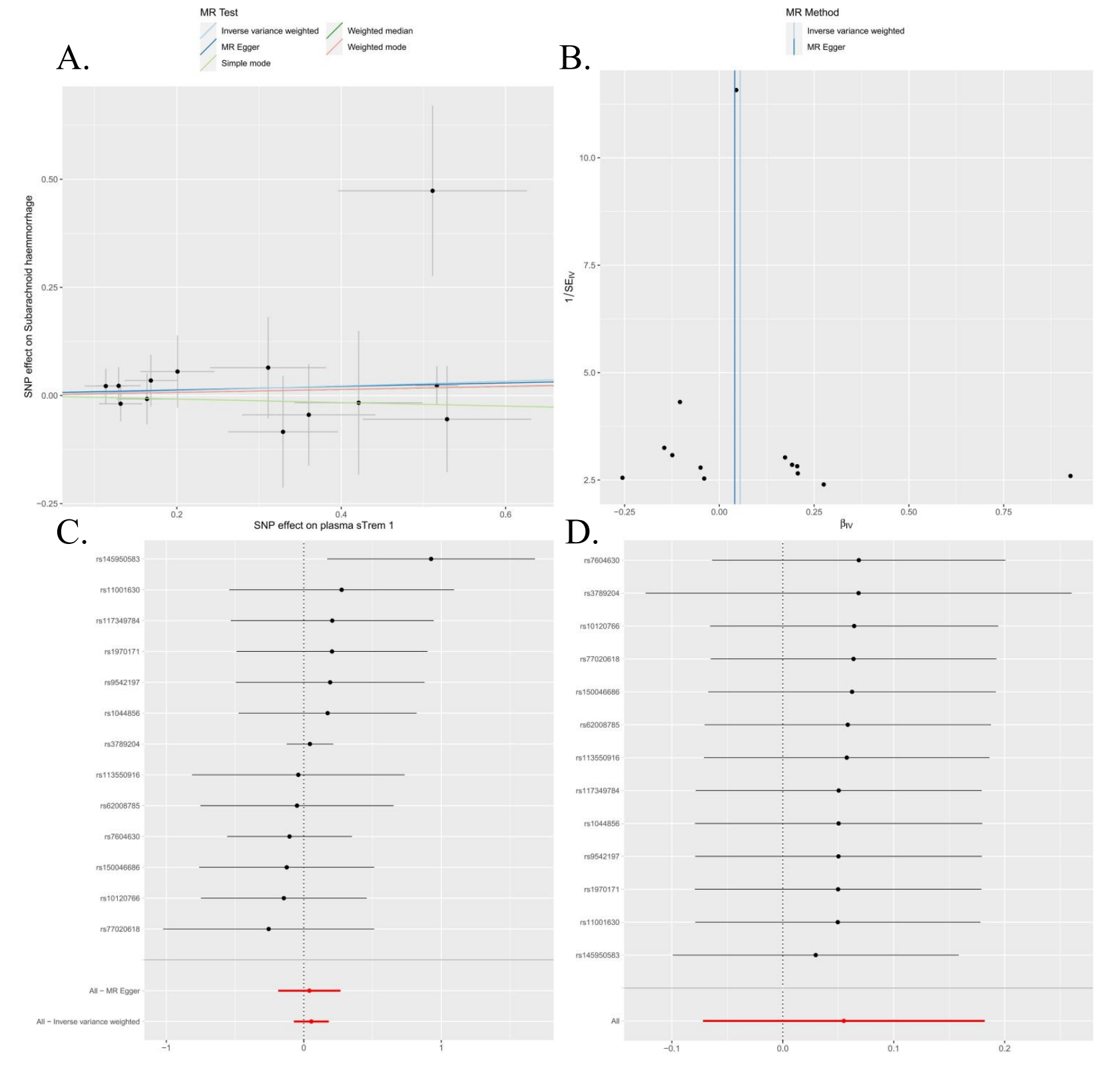
**

**Figure S13.** The causal effect of plasma sTrem1 on migraine (with aura) risk. (A) Scatter plot, (B) Funnel plot, (C) Forest plot, and (D) Leave one out plot.

**
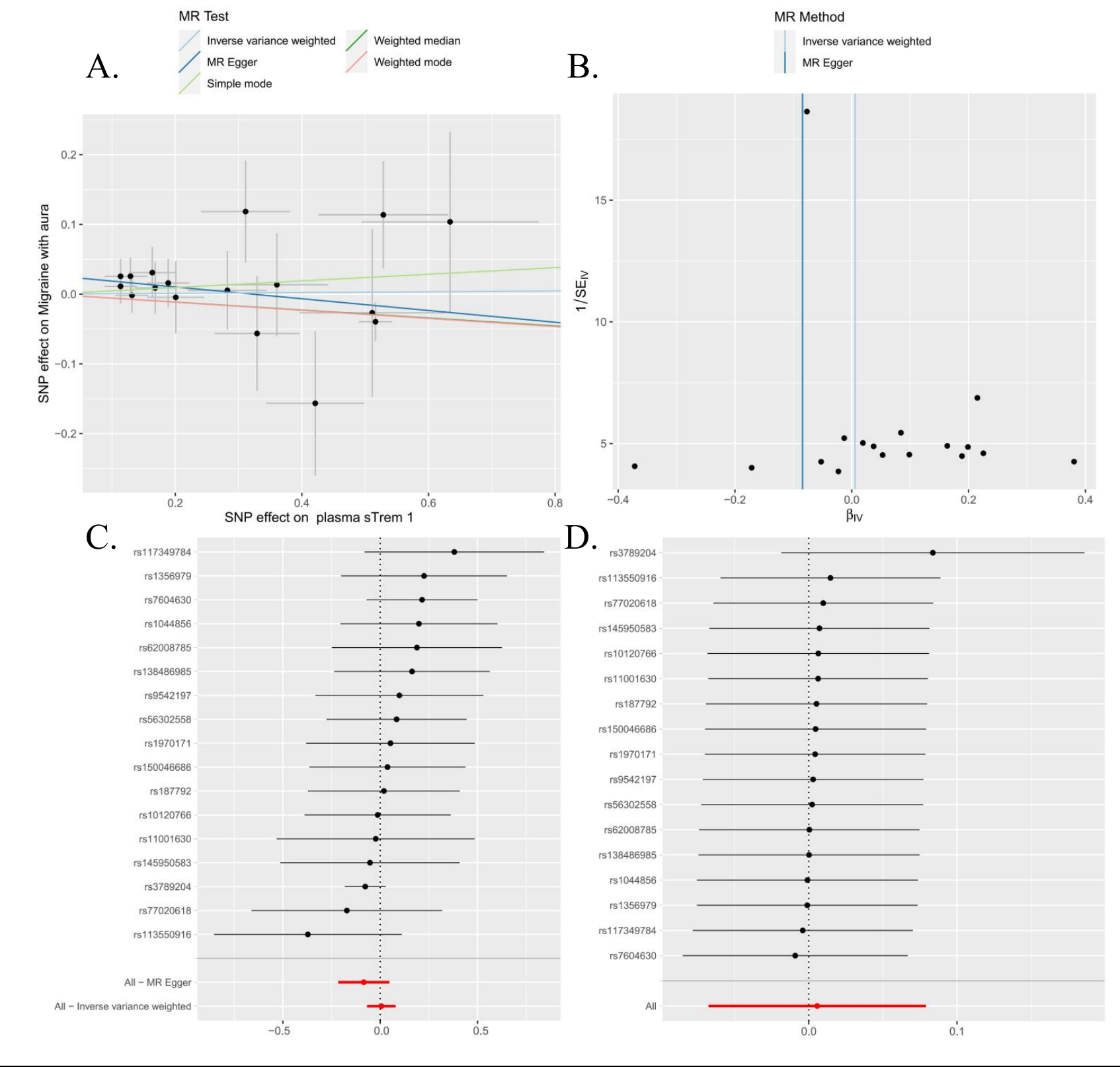
**

**Figure S14.** The causal effect of plasma sTrem1 on migraine (without aura, drug-induced) risk. (A) Scatter plot, (B) Funnel plot, (C) Forest plot, and (D) Leave one out plot.

**
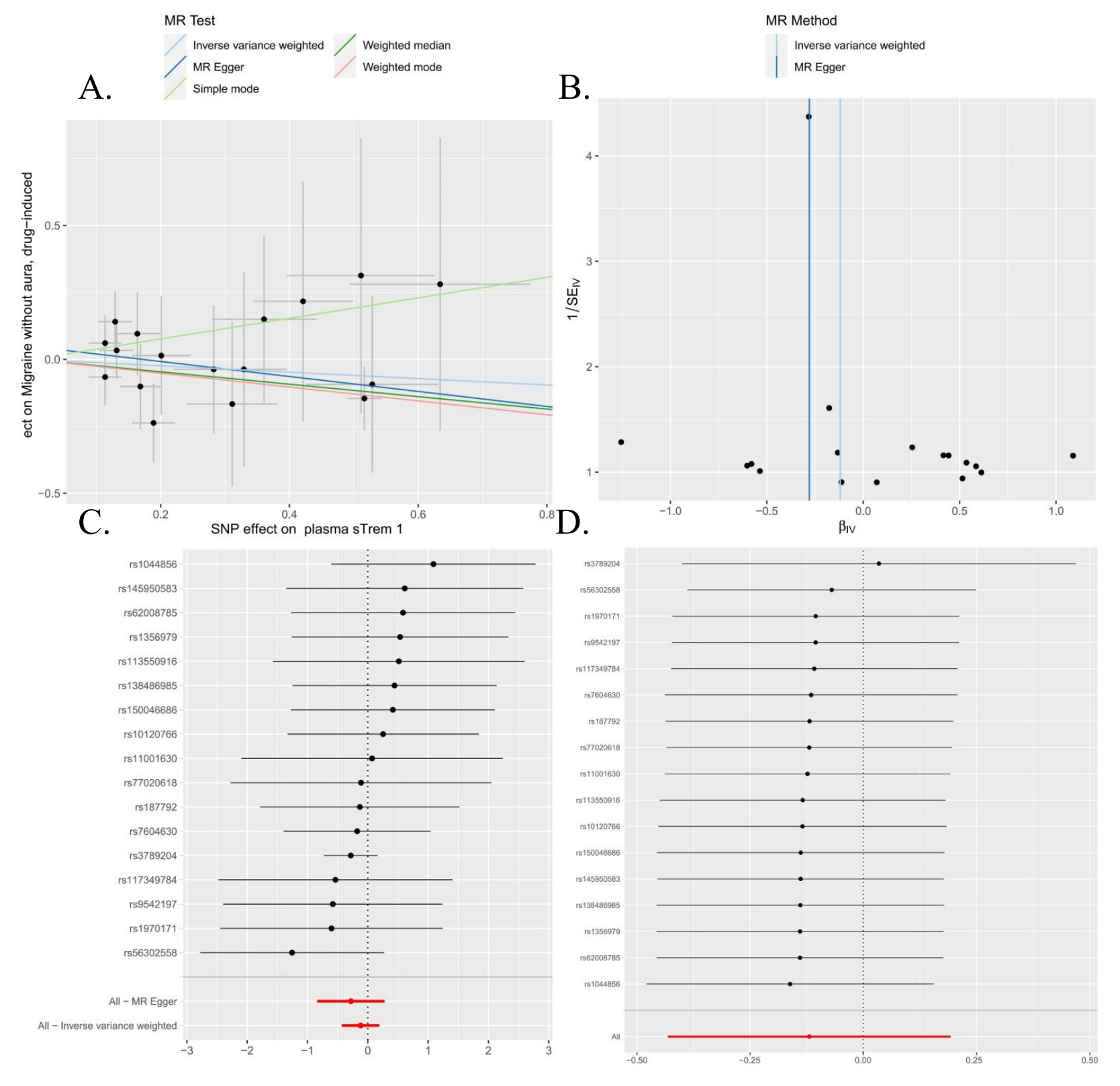
**

**Figure S15**. The causal effect of plasma sTrem1 on migraine (without aura and triptan purchases) risk. (A) Scatter plot, (B) Funnel plot, (C) Forest plot, and (D) Leave one out plot.

**
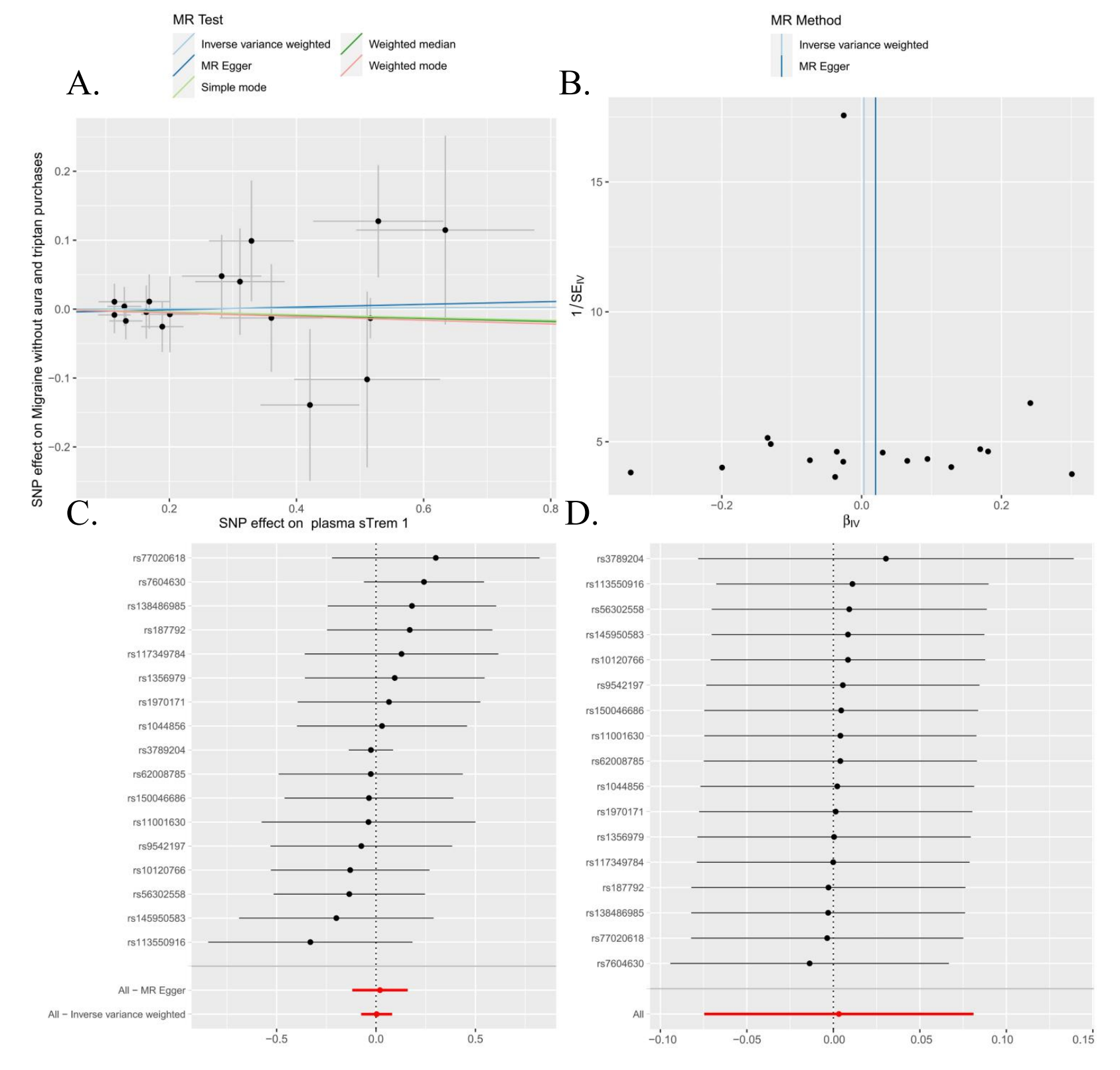
**
