# Supplementary material for: The associations between plasma soluble Trem1 and neurological diseases: a Mendelian randomization study
Source: J Neuroinflammation. 2022 Sep 6;19:218. doi: 10.1186/s12974-022-02582-z (PMC9446564; doi:10.1186/s12974-022-02582-z)
Supplement: Supplementary file 2 — Additional file 2. Information of investigators of each GWAS dataset in the MR study. [file 12974_2022_2582_MOESM2_ESM.docx]

**Additional File:**

**Supplemental TEXT. Information of investigators of each GWAS dataset in the MR study.**

**1. Genomic atlas of the human plasma proteome**

Benjamin B Sun^1^, Joseph C Maranville^2,3^, James E Peters^1,4^, David Stacey^1^, James R Staley^1^, James Blackshaw^1^, Stephen Burgess^1,5^, Tao Jiang^1^, Ellie Paige^1,6^, Praveen Surendran^1^, Clare Oliver-Williams^1,7^, Mihir A Kamat^1^, Bram P Prins^1^, Sheri K Wilcox^8^, Erik S Zimmerman^8^, An Chi^2^, Narinder Bansal^1,9^, Sarah L Spain^10^, Angela M Wood^1^, Nicholas W Morrell^4,11^, John R Bradley^12^, Nebojsa Janjic^8^, David J Roberts^13,14^, Willem H Ouwehand^4,15,16,17,18^, John A Todd^19^, Nicole Soranzo^4,15,17,18^, Karsten Suhre^20^, Dirk S Paul^1^, Caroline S Fox^2^, Robert M Plenge^2,3^, John Danesh^21,22,23,24^, Heiko Runz^2,25^, Adam S Butterworth^26,27^

^1^MRC/BHF Cardiovascular Epidemiology Unit, Department of Public Health and Primary Care, University of Cambridge, Cambridge, UK.

^2^MRL, Merck & Co., Inc., Kenilworth, NJ, USA.

^3^Celgene Inc., Cambridge, MA, USA.

^4^British Heart Foundation Cambridge Centre of Excellence, Division of Cardiovascular Medicine, Addenbrooke's Hospital, Cambridge, UK.

^5^MRC Biostatistics Unit, University of Cambridge, Cambridge, UK.

^6^National Centre for Epidemiology and Population Health, The Australian National University, Canberra, Australian Capital Territory, Australia.

^7^Homerton College, Cambridge, UK.

^8^SomaLogic Inc, Boulder, CO, USA.

^9^Population Health Sciences, Bristol Medical School, University of Bristol, Bristol, UK.

^10^Wellcome Trust Sanger Institute, Wellcome Trust Genome Campus, Hinxton, Cambridge, UK.

^11^Division of Respiratory Medicine, Department of Medicine, University of Cambridge, Cambridge, UK.

^12^NIHR Cambridge Biomedical Research Centre/BioResource, Cambridge University Hospitals, Cambridge, UK.

^13^National Health Service (NHS) Blood and Transplant and Radcliffe Department of Medicine, NIHR Oxford Biomedical Research Centre, University of Oxford, John Radcliffe Hospital, Oxford, UK.

^14^BRC Haematology Theme and Department of Haematology, Churchill Hospital, Oxford, UK.

^15^Department of Haematology, University of Cambridge, Cambridge Biomedical Campus, Cambridge, UK.

^16^National Health Service (NHS) Blood and Transplant, Cambridge Biomedical Campus, Cambridge, UK.

^17^Department of Human Genetics, Wellcome Trust Sanger Institute, Wellcome Trust Genome Campus, Hinxton, Cambridge, UK.

^18^NIHR Blood and Transplant Research Unit in Donor Health and Genomics, Department of Public Health and Primary Care, University of Cambridge, Cambridge, UK.

^19^JDRF/Wellcome Trust Diabetes and Inflammation Laboratory, Wellcome Trust Centre for Human Genetics, Nuffield Department of Medicine, NIHR Oxford Biomedical Research Centre, University of Oxford, Oxford, UK.

^20^Department of Physiology and Biophysics, Weill Cornell Medicine-Qatar, Doha, Qatar.

^21^MRC/BHF Cardiovascular Epidemiology Unit, Department of Public Health and Primary Care, University of Cambridge, Cambridge, UK. jd292@medschl.cam.ac.uk.

^22^British Heart Foundation Cambridge Centre of Excellence, Division of Cardiovascular Medicine, Addenbrooke's Hospital, Cambridge, UK. jd292@medschl.cam.ac.uk.

^23^Department of Human Genetics, Wellcome Trust Sanger Institute, Wellcome Trust Genome Campus, Hinxton, Cambridge, UK. jd292@medschl.cam.ac.uk.

^24^NIHR Blood and Transplant Research Unit in Donor Health and Genomics, Department of Public Health and Primary Care, University of Cambridge, Cambridge, UK. jd292@medschl.cam.ac.uk.

^25^Biogen Inc., Cambridge, MA, USA.

^26^MRC/BHF Cardiovascular Epidemiology Unit, Department of Public Health and Primary Care, University of Cambridge, Cambridge, UK. asb38@medschl.cam.ac.uk.

^27^NIHR Blood and Transplant Research Unit in Donor Health and Genomics, Department of Public Health and Primary Care, University of Cambridge, Cambridge, UK. asb38@medschl.cam.ac.uk.

**2. Genetic meta-analysis of diagnosed Alzheimer's disease identifies new risk loci and implicates Aβ, tau, immunity and lipid processing**

Brian W Kunkle^1^, Benjamin Grenier-Boley^2,3,4^, Rebecca Sims^5,6^, Joshua C Bis^7^, Vincent Damotte^2,3,4^, Adam C Naj^8^, Anne Boland^9^, Maria Vronskaya^5^, Sven J van der Lee^10^, Alexandre Amlie-Wolf^11^, Céline Bellenguez^2 3 4^, Aura Frizatti^5^, Vincent Chouraki^2,3,4,12,13^, Eden R Martin ^14^, Kristel Sleegers^15,16^, Nandini Badarinarayan^5^, Johanna Jakobsdottir^17^, Kara L Hamilton-Nelson^14^, Sonia Moreno-Grau^18,19^, Robert Olaso^9^, Rachel Raybould^5,6^, Yuning Chen^20^, Amanda B Kuzma^11^, Mikko Hiltunen^21,22^, Taniesha Morgan^5^, Shahzad Ahmad^10^, Badri N Vardarajan^23,24,25^, Jacques Epelbaum^26^, Per Hoffmann^27,28,29^, Merce Boada^18,19^, Gary W Beecham^14^, Jean-Guillaume Garnier^9^, Denise Harold^30^, Annette L Fitzpatrick^31,32^, Otto Valladares^11^, Marie-Laure Moutet^9^, Amy Gerrish^33^, Albert V Smith^34,35^, Liming Qu^11^, Delphine Bacq^9^, Nicola Denning^5,6^, Xueqiu Jian^36^, Yi Zhao^11^, Maria Del Zompo^37^, Nick C Fox^33,38^, Seung-Hoan Choi^18^, Ignacio Mateo^39^, Joseph T Hughes^40^, Hieab H Adams^10^, John Malamon^11^, Florentino Sanchez-Garcia^41^, Yogen Patel^40^, Jennifer A Brody^7^, Beth A Dombroski^11^, Maria Candida Deniz Naranjo^41^, Makrina Daniilidou^42^, Gudny Eiriksdottir^17^, Shubhabrata Mukherjee^43^, David Wallon^44^, James Uphill^45^, Thor Aspelund^17,46^, Laura B Cantwell^11^, Fabienne Garzia^9^, Daniela Galimberti^47,48^, Edith Hofer^49,50^, Mariusz Butkiewicz^51^, Bertrand Fin^9^, Elio Scarpini^47,48^, Chloe Sarnowski^20^, Will S Bush^51^, Stéphane Meslage^9^, Johannes Kornhuber^52^, Charles C White^53^, Yuenjoo Song^51^, Robert C Barber^54^, Sebastiaan Engelborghs ^55,56^, Sabrina Sordon^57^, Dina Voijnovic^10^, Perrie M Adams^58^, Rik Vandenberghe^59^, Manuel Mayhaus^57^, L Adrienne Cupples^12,20^, Marilyn S Albert^60^, Peter P De Deyn^55,56^, Wei Gu^57^, Jayanadra J Himali^12,13,20^, Duane Beekly^61^, Alessio Squassina^37^, Annette M Hartmann^62^, Adelina Orellana^18^, Deborah Blacker^63,64^, Eloy Rodriguez-Rodriguez^39^, Simon Lovestone^65^, Melissa E Garcia^66^, Rachelle S Doody^67^, Carmen Munoz-Fernadez^41^, Rebecca Sussams^68^, Honghuang Lin^69^, Thomas J Fairchild^70^, Yolanda A Benito^41^, Clive Holmes^68^, Hata Karamujić-Čomić^10^, Matthew P Frosch^71^, Hakan Thonberg^72,73^, Wolfgang Maier^74,75^, Gennady Roshchupkin^10^, Bernardino Ghetti^76^, Vilmantas Giedraitis^77^, Amit Kawalia^78^, Shuo Li^20^, Ryan M Huebinger^79^, Lena Kilander^77^, Susanne Moebus^80^, Isabel Hernández^18,19^, M Ilyas Kamboh^81,82,83^, RoseMarie Brundin^77^, James Turto^84^, Qiong Yang^20^, Mindy J Katz^85^, Letizia Concari^86,87^, Jenny Lord^84^, Alexa S Beiser^12,13,20^, C Dirk Keene^88^, Seppo Helisalmi^21,22^, Iwona Kloszewska^89^, Walter A Kukull^32^, Anne Maria Koivisto^21,22^, Aoibhinn Lynch^90,91^, Lluís Tarraga ^18,19^, Eric B Larson^92^, Annakaisa Haapasalo^93^, Brian Lawlor^90,91^, Thomas H Mosley^94^, Richard B Lipton^85^, Vincenzo Solfrizzi^95^, Michael Gill^90,91^, W T Longstreth Jr^32,96^, Thomas J Montine^88^, Vincenza Frisardi^97^, Monica Diez-Fairen^98,99^, Fernando Rivadeneira^10,100,101^, Ronald C Petersen^102^, Vincent Deramecourt^103^, Ignacio Alvarez^98,99^, Francesca Salani^104^, Antonio Ciaramella^104^, Eric Boerwinkle^105,106^, Eric M Reiman^107,108,109,110^, Nathalie Fievet^2,3,4^, Jerome I Rotter^111^, Joan S Reisch^112^, Olivier Hanon^113^, Chiara Cupidi^114^, A G Andre Uitterlinden^10,100,101^, Donald R Royall^115^, Carole Dufouil^116,117^, Raffaele Giovanni Maletta^114^, Itziar de Rojas^18,19^, Mary Sano^118^, Alexis Brice^119,120^, Roberta Cecchetti^121^, Peter St George-Hyslop^122,123^, Karen Ritchie^124,125,126^, Magda Tsolaki^42^, Debby W Tsuang^127,128^, Bruno Dubois^129,130,131,132^, David Craig^133^, Chuang-Kuo Wu^134^, Hilkka Soininen^21,22^, Despoina Avramidou^42^, Roger L Albin^135,136,137^, Laura Fratiglioni^138^, Antonia Germanou^42^, Liana G Apostolova^139,140,141,142^, Lina Keller^138^, Maria Koutroumani^42^, Steven E Arnold^143^, Francesco Panza^97^, Olymbia Gkatzima^42^, Sanjay Asthana^144,145,146^, Didier Hannequin^40^, Patrice Whitehead^14^, Craig S Atwood^140,141,142^, Paolo Caffarra^83,84^, Harald Hampel^147,148,149,150^, Inés Quintela^151^, Ángel Carracedo^151^, Lars Lannfelt^77^, David C Rubinsztein^122,152^, Lisa L Barnes^153,154,155^, Florence Pasquier^103^, Lutz Frölich^156^, Sandra Barral^23,24,25^, Bernadette McGuinness^133^, Thomas G Beach^157^, Janet A Johnston^133^, James T Becker^81,158,159^, Peter Passmore^133^, Eileen H Bigio^160,161^, Jonathan M Schott ^33^, Thomas D Bird^96,127^, Jason D Warren^33^, Bradley F Boeve^102^, Michelle K Lupton^40,162^, James D Bowen^163^, Petra Proitsi^40^, Adam Boxer^164^, John F Powell^40^, James R Burke^165^, John S K Kauwe^166^, Jeffrey M Burns^167^, Michelangelo Mancuso^168^, Joseph D Buxbaum^118,169,170^, Ubaldo Bonuccelli^168^, Nigel J Cairns^171^, Andrew McQuillin^172^, Chuanhai Cao^173^, Gill Livingston^172^, Chris S Carlson^145,146^, Nicholas J Bass^172^, Cynthia M Carlsson^174^, John Hardy^175^, Regina M Carney^176^, Jose Bras^38,177^, Minerva M Carrasquillo^178^, Rita Guerreiro^38,177^, Mariet Allen^178^, Helena C Chui^179^, Elizabeth Fisher^177^, Carlo Masullo^180^, Elizabeth A Crocco^181^, Charles DeCarli ^182^, Gina Bisceglio^178^, Malcolm Dick^183^, Li Ma^178^, Ranjan Duara^184^, Neill R Graff-Radford^178^, Denis A Evans^185^, Angela Hodges^186^, Kelley M Faber^139^, Martin Scherer^187^, Kenneth B Fallon^188^, Matthias Riemenschneider^57^, David W Fardo^189^, Reinhard Heun^75^, Martin R Farlow^141^, Heike Kölsch^75^, Steven Ferris^190^, Markus Leber^191^, Tatiana M Foroud^139^, Isabella Heuser^192^, Douglas R Galasko^193^, Ina Giegling^62^, Marla Gearing^194,195^, Michael Hüll^196^, Daniel H Geschwind^197^, John R Gilbert^14^, John Morris^198,199^, Robert C Green^200^, Kevin Mayo^198,201,202^, John H Growdon^203^, Thomas Feulner^57^, Ronald L Hamilton^204^, Lindy E Harrell^205^, Dmitriy Drichel^206^, Lawrence S Honig^23^, Thomas D Cushion^5,6^, Matthew J Huentelman^107^, Paul Hollingworth^5^, Christine M Hulette^207^, Bradley T Hyman^203^, Rachel Marshall^5^, Gail P Jarvik^208,209^, Alun Meggy^5^, Erin Abner^210^, Georgina E Menzies^5,6^, Lee-Way Jin^211^, Ganna Leonenko^5^, Luis M Real^211^, Gyungah R Jun^212^, Clinton T Baldwin^212^, Detelina Grozeva^5^, Anna Karydas^163^, Giancarlo Russo^213^, Jeffrey A Kaye^214,215^, Ronald Kim^216^, Frank Jessen^74,75,191^, Neil W Kowall^13,217^, Bruno Vellas^218^, Joel H Kramer^219^, Emma Vardy^220^, Frank M LaFerla^221^, Karl-Heinz Jöckel^80^, James J Lah^222^, Martin Dichgans^223,224^, James B Leverenz^225^, David Mann^226^, Allan I Levey^222^, Stuart Pickering-Brown^226^, Andrew P Lieberman^227^, Norman Klopp^228^, Kathryn L Lunetta^20^, H-Erich Wichmann^229,230,231^, Constantine G Lyketsos^232^, Kevin Morgan^233^, Daniel C Marson^205^, Kristelle Brown^84^, Frank Martiniuk^234^, Christopher Medway^84^, Deborah C Mash^235^, Markus M Nöthen^27,28^, Eliezer Masliah^193,236^, Nigel M Hooper^226^, Wayne C McCormick^43^, Antonio Daniele^237^, Susan M McCurry^238^, Anthony Bayer^239^, Andrew N McDavid^173^, John Gallacher^65^, Ann C McKee^13,217^, Hendrik van den Bussche^187^, Marsel Mesulam^161, 240^, Carol Brayne^241^, Bruce L Miller^242^, Steffi Riedel-Heller^243^, Carol A Miller^244^, Joshua W Miller^245^, Ammar Al-Chalabi^246^, John C Morris^171,201^, Christopher E Shaw^246,247^, Amanda J Myers^181^, Jens Wiltfang^248,249,250^, Sid O'Bryant^54^, John M Olichney^182^, Victoria Alvarez^251^, Joseph E Parisi^252^, Andrew B Singleton^253^, Henry L Paulson^135,137^, John Collinge^45^, William R Perry^14^, Simon Mead^45^, Elaine Peskind^128^, David H Cribbs^254^, Martin Rossor^33^, Aimee Pierce^254^, Natalie S Ryan^45^, Wayne W Poon^183^, Benedetta Nacmias^255,256^, Huntington Potter^257^, Sandro Sorbi^255,258^, Joseph F Quinn^187,188^, Eleonora Sacchinelli^104^, Ashok Raj^173^, Gianfranco Spalletta^259,260^, Murray Raskind^128^, Carlo Caltagirone^259^, Paola Bossù^104^, Maria Donata Orfei^259^, Barry Reisberg^190,261^, Robert Clarke^262^, Christiane Reitz^23,24,263^, A David Smith^264^, John M Ringman^265^, Donald Warden^264^, Erik D Roberson^205^, Gordon Wilcock ^264^, Ekaterina Rogaeva^123^, Amalia Cecilia Bruni^114^, Howard J Rosen^164^, Maura Gallo^114^, Roger N Rosenberg^266^, Yoav Ben-Shlomo^267^, Mark A Sager^145^, Patrizia Mecocci^121^, Andrew J Saykin^139,141^, Pau Pastor^98,99^, Michael L Cuccaro^14^, Jeffery M Vance^14^, Julie A Schneider^153,155,268^, Lori S Schneider^179,269^, Susan Slifer^14^, William W Seeley^164^, Amanda G Smith^173^, Joshua A Sonnen^88^, Salvatore Spina^76^, Robert A Stern^13^, Russell H Swerdlow^167^, Mitchell Tang^11^, Rudolph E Tanzi^203^, John Q Trojanowski^270^, Juan C Troncoso^271^, Vivianna M Van Deerlin^270^, Linda J Van Eldik^272^, Harry V Vinters^273,274^, Jean Paul Vonsattel^275^, Sandra Weintraub^161,276^, Kathleen A Welsh-Bohmer^165,277^, Kirk C Wilhelmsen^278^, Jennifer Williamson^23^, Thomas S Wingo^222,279^, Randall L Woltjer^280^, Clinton B Wright^281^, Chang-En Yu ^43^, Lei Yu^153,155^, Yasaman Saba^282^, Alberto Pilotto^283,284^, Maria J Bullido^19,285,286^, Oliver Peters ^192,287^, Paul K Crane^43^, David Bennett^153,155^, Paola Bosco^288^, Eliecer Coto^251^, Virginia Boccardi^121^, Phil L De Jager^289^, Alberto Lleo^19,290^, Nick Warner^291^, Oscar L Lopez^81,83,158^, Martin Ingelsson^77^, Panagiotis Deloukas^292^, Carlos Cruchaga^198,199^, Caroline Graff^72,73^, Rhian Gwilliam^292^, Myriam Fornage^36^, Alison M Goate^169,293^, Pascual Sanchez-Juan^39^, Patrick G Kehoe^294^, Najaf Amin^10^, Nilifur Ertekin-Taner^178,295^, Claudine Berr^124,125^, Stéphanie Debette^119,120^, Seth Love^294^, Lenore J Launer^66^, Steven G Younkin^178,295^, Jean-Francois Dartigues^296^, Chris Corcoran^297^, M Arfan Ikram^10,298,299^, Dennis W Dickson^178^, Gael Nicolas^44^, Dominique Campion^44,300^, JoAnn Tschanz^297^, Helena Schmidt^282,301^, Hakon Hakonarson^302,303^, Jordi Clarimon^19,290^, Ron Munger^297^, Reinhold Schmidt^49^, Lindsay A Farrer^13,20,212,304,305^, Christine Van Broeckhoven^15,16^, Michael C O'Donovan^5^, Anita L DeStefano^13,20^, Lesley Jones^5,6^, Jonathan L Haines^51^, Jean-Francois Deleuze^9^, Michael J Owen^5^, Vilmundur Gudnason^17,35^, Richard Mayeux^23,24,25^, Valentina Escott-Price^5,6^, Bruce M Psaty^7,32,306,307^, Alfredo Ramirez^78,191^, Li-San Wang^11^, Agustin Ruiz^18,19^, Cornelia M van Duijn^10^, Peter A Holmans^5^, Sudha Seshadri^12,13,308^, Julie Williams^5,6^, Phillippe Amouyel^2,3,4,309^, Gerard D Schellenberg^11^, Jean-Charles Lambert^310,311,312^, Margaret A Pericak-Vance^313^, Alzheimer Disease Genetics Consortium (ADGC),; European Alzheimer’s Disease Initiative (EADI),; Cohorts for Heart and Aging Research in Genomic Epidemiology Consortium (CHARGE),; Genetic and Environmental Risk in AD/Defining Genetic, Polygenic and Environmental Risk for Alzheimer’s Disease Consortium (GERAD/PERADES),

^1^John P. Hussman Institute for Human Genomics, University of Miami Miller School of Medicine, Miami, FL, USA. bkunkle@miami.edu.

^2^Inserm, U1167, RID-AGE-Risk Factors and Molecular Determinants of Aging-Related Diseases, Lille, France.

^3^Institut Pasteur de Lille, Lille, France.

^4^Univ. Lille, U1167-Excellence Laboratory LabEx DISTALZ, Lille, France.

^5^Division of Psychological Medicine and Clinical Neurosciences, MRC Centre for Neuropsychiatric Genetics and Genomics, Cardiff University, Cardiff, UK.

^6^UK Dementia Research Institute at Cardiff, Cardiff University, Cardiff, UK.

^7^Cardiovascular Health Research Unit, Department of Medicine, University of Washington, Seattle, WA, USA.

^8^Department of Biostatistics and Epidemiology/Center for Clinical Epidemiology and Biostatistics, University of Pennsylvania Perelman School of Medicine, Philadelphia, PA, USA.

^9^Centre National de Recherche en Génomique Humaine, Institut de Biologie François Jacob, CEA, Université Paris-Saclay, and LabEx GENMED, Evry, France.

^10^Department of Epidemiology, Erasmus Medical Center, Rotterdam, the Netherlands.

^11^Penn Neurodegeneration Genomics Center, Department of Pathology and Laboratory Medicine, University of Pennsylvania Perelman School of Medicine, Philadelphia, PA, USA.

^12^Framingham Heart Study, Framingham, MA, USA.

^13^Department of Neurology, Boston University School of Medicine, Boston, MA, USA.

^14^John P. Hussman Institute for Human Genomics, University of Miami Miller School of Medicine, Miami, FL, USA.

^15^Neurodegenerative Brain Diseases Group, Center for Molecular Neurology, VIB, Antwerp, Belgium.

^16^Laboratory for Neurogenetics, Institute Born-Bunge, University of Antwerp, Antwerp, Belgium.

^17^Icelandic Heart Association, Kopavogur, Iceland.

^18^Research Center and Memory Clinic of Fundació ACE, Institut Català de Neurociències Aplicades-Universitat Internacional de Catalunya, Barcelona, Spain.

^19^Centro de Investigación Biomédica en Red de Enfermedades Neurodegenerativas, Instituto de Salud Carlos III, Madrid, Spain.

^20^Department of Biostatistics, Boston University School of Public Health, Boston, MA, USA.

^21^Institute of Biomedicine, University of Eastern Finland, Kuopio, Finland.

^22^Department of Neurology, Kuopio University Hospital, Kuopio, Finland.

^23^Taub Institute on Alzheimer's Disease and the Aging Brain, Department of Neurology, Columbia University, New York, NY, USA.

^24^Gertrude H. Sergievsky Center, Columbia University, New York, NY, USA.

^25^Department of Neurology, Columbia University, New York, NY, USA.

^26^UMR 894, Center for Psychiatry and Neuroscience, Inserm, Université Paris Descartes, Paris, France.

^27^Institute of Human Genetics, University of Bonn, Bonn, Germany.

^28^Department of Genomics, Life & Brain Center, University of Bonn, Bonn, Germany.

^29^Division of Medical Genetics, University Hospital and Department of Biomedicine, University of Basel, Basel, Switzerland.

^30^School of Biotechnology, Dublin City University, Dublin, Ireland.

^31^Department of Family Medicine, University of Washington, Seattle, WA, USA.

^32^Department of Epidemiology, University of Washington, Seattle, WA, USA.

^33^Dementia Research Centre, Department of Neurodegenerative Disease, UCL Institute of Neurology, London, UK.

^34^Department of Biostatistics, University of Michigan, Ann Arbor, MI, USA.

^35^Faculty of Medicine, University of Iceland, Reykjavik, Iceland.

^36^Brown Foundation Institute of Molecular Medicine, University of Texas Health Sciences Center at Houston, Houston, TX, USA.

^37^Section of Neuroscience and Clinical Pharmacology, Department of Biomedical Sciences, University of Cagliari, Cagliari, Italy.

^38^UK Dementia Research Institute at UCL, Department of Neurodegenerative Disease, UCL Institute of Neurology, London, UK.

^39^Neurology Service and CIBERNED, 'Marqués de Valdecilla' University Hospital (University of Cantabria and IDIVAL), Santander, Spain.

^40^Department of Basic and Clinical Neuroscience, Institute of Psychiatry, Psychology and Neuroscience, King's College London, London, UK.

^41^Department of Immunology, Hospital Universitario Doctor Negrín, Las Palmas de Gran Canaria, Spain.

^42^Department of Neurology, Medical School, Aristotle University of Thessaloniki, Thessaloniki, Greece.

^43^Department of Medicine, University of Washington, Seattle, WA, USA.

^44^Normandie University, UNIROUEN, Inserm U1245, and Rouen University Hospital, Department of Neurology, Department of Genetics and CNR-MAJ, Normandy Center for Genomic and Personalized Medicine, Rouen, France.

^45^Department of Neurodegenerative Disease, MRC Prion Unit at UCL, Institute of Prion Diseases, London, UK.

^46^Centre for Public Health, University of Iceland, Reykjavik, Iceland.

^47^Fondazione IRCCS Ca' Granda, Ospedale Maggiore Policlinico, Neurodegenerative Diseases Unit, Milan, Italy.

^48^University of Milan, Centro Dino Ferrari, Milan, Italy.

^49^Clinical Division of Neurogeriatrics, Department of Neurology, Medical University Graz, Graz, Austria.

^50^Institute for Medical Informatics, Statistics and Documentation, Medical University of Graz, Graz, Austria.

^51^Institute for Computational Biology, Department of Population & Quantitative Health Sciences, Case Western Reserve University, Cleveland, OH, USA.

^52^Department of Psychiatry and Psychotherapy, University of Erlangen-Nuremberg, Erlangen, Germany.

^53^Program in Medical and Population Genetics, Broad Institute, Cambridge, MA, USA.

^54^Department of Pharmacology and Neuroscience, University of North Texas Health Science Center, Fort Worth, TX, USA.

^55^Laboratory for Neurochemistry and Behavior, Institute Born-Bunge, University of Antwerp, Antwerp, Belgium.

^56^Department of Neurology and Memory Clinic, Hospital Network Antwerp, Antwerp, Belgium.

^57^Department of Psychiatry and Psychotherapy, University Hospital, Saarland, Germany.

^58^Department of Psychiatry, University of Texas Southwestern Medical Center, Dallas, TX, USA.

^59^Laboratory for Cognitive Neurology, Department of Neurology, University Hospital and University of Leuven, Leuven, Belgium.

^60^Department of Neurology, Johns Hopkins University, Baltimore, MD, USA.

^61^National Alzheimer's Coordinating Center, University of Washington, Seattle, WA, USA.

^62^Department of Psychiatry, Martin Luther University Halle-Wittenberg, Halle, Germany.

^63^Department of Epidemiology, Harvard T.H. Chan School of Public Health, Harvard University, Boston, MA, USA.

^64^Department of Psychiatry, Massachusetts General Hospital/Harvard Medical School, Boston, MA, USA.

^65^Department of Psychiatry, University of Oxford, Oxford, UK.

^66^Laboratory of Epidemiology and Population Sciences, National Institute on Aging, Bethesda, MD, USA.

^67^Alzheimer's Disease and Memory Disorders Center, Baylor College of Medicine, Houston, TX, USA.

^68^Division of Clinical Neurosciences, School of Medicine, University of Southampton, Southampton, UK.

^69^Section of Computational Biomedicine, Department of Medicine, Boston University School of Medicine, Boston, MA, USA.

^70^Office of Strategy and Measurement, University of North Texas Health Science Center, Fort Worth, TX, USA.

^71^C.S. Kubik Laboratory for Neuropathology, Massachusetts General Hospital, Charlestown, MA, USA.

^72^Theme Aging, Unit for Hereditary Dementias, Karolinska University Hospital-Solna, Stockholm, Sweden.

^73^Karolinska Institutet, Department of Neurobiology, Care Sciences and Society, Alzheimer Research Center, Division of Neurogeriatrics, Solna, Sweden.

^74^German Center for Neurodegenerative Diseases, Bonn, Germany.

^75^Department of Psychiatry and Psychotherapy, University of Bonn, Bonn, Germany.

^76^Department of Pathology and Laboratory Medicine, Indiana University, Indianapolis, IN, USA.

^77^Department of Public Health and Caring Sciences/Geriatrics, Uppsala University, Uppsala, Sweden.

^78^Department for Neurodegenerative Diseases and Geriatric Psychiatry, University Hospital Bonn, Bonn, Germany.

^79^Department of Surgery, University of Texas Southwestern Medical Center, Dallas, TX, USA.

80Institute for Medical Informatics, Biometry and Epidemiology, University Hospital of Essen, University Duisburg-Essen, Essen, Germany.

^81^Department of Psychiatry, University of Pittsburgh, Pittsburgh, PA, USA.

^82^Department of Human Genetics, University of Pittsburgh, Pittsburgh, PA, USA.

^83^Alzheimer's Disease Research Center, University of Pittsburgh, Pittsburgh, PA, USA.

^84^Institute of Genetics, Queen's Medical Centre, University of Nottingham, Nottingham, UK.

^85^Department of Neurology, Albert Einstein College of Medicine, Bronx, NY, USA.

^86^Section of Neuroscience, DIMEC-University of Parma, Parma, Italy.

^87^FERB-Alzheimer Center, Gazzaniga (Bergamo), Italy.

^88^Department of Pathology, University of Washington, Seattle, WA, USA.

^89^Elderly and Psychiatric Disorders Department, Medical University of Lodz, Lodz, Poland.

^90^Mercer's Institute for Research on Aging, St. James's Hospital and Trinity College, Dublin, Ireland.

^91^St. James's Hospital and Trinity College, Dublin, Ireland.

^92^Kaiser Permanente Washington Health Research Institute, Seattle, WA, USA.

^93^A.I. Virtanen Institute for Molecular Sciences, University of Eastern Finland, Kuopio, Finland.

^94^Departments of Medicine, Geriatrics, Gerontology and Neurology, University of Mississippi Medical Center, Jackson, MS, USA.

^95^Interdisciplinary Department of Medicine, Geriatric Medicine and Memory Unity, University of Bari, Bari, Italy.

^96^Department of Neurology, University of Washington, Seattle, WA, USA.

^97^Department of Geriatrics, Center for Aging Brain, University of Bari, Bari, Italy.

^98^Fundació per la Recerca Biomèdica i Social Mútua Terrassa, Terrassa, Barcelona, Spain.

^99^Memory Disorders Unit, Department of Neurology, Hospital Universitari Mutua de Terrassa, Terrassa, Barcelona, Spain.

^100^Department of Internal Medicine, Erasmus University Medical Center, Rotterdamt, the Netherlands.

^101^Netherlands Consortium on Health Aging and National Genomics Initiative, Leiden, the Netherlands.

^102^Department of Neurology, Mayo Clinic, Rochester, MN, USA.

^103^CHU Lille, Memory Center of Lille (Centre Mémoire de Ressources et de Recherche), Lille, France.

^104^Department of Clinical and Behavioral Neurology, Experimental Neuropsychobiology Laboratory, IRCCS Santa Lucia Foundation, Rome, Italy.

^105^School of Public Health, Human Genetics Center, University of Texas Health Science Center at Houston, Houston, TX, USA.

^106^Human Genome Sequencing Center, Baylor College of Medicine, Houston, TX, USA.

^107^Neurogenomics Division, Translational Genomics Research Institute, Phoenix, AZ, USA.

^108^Arizona Alzheimer's Consortium, Phoenix, AZ, USA.

^109^Banner Alzheimer's Institute, Phoenix, AZ, USA.

^110^Department of Psychiatry, University of Arizona, Phoenix, AZ, USA.

^111^Institute for Translational Genomics and Population Sciences, Departments of Pediatrics and Medicine, Los Angeles BioMedical Research Institute at Harbor-UCLA Medical Center, Torrance, CA, USA.

^112^Department of Clinical Sciences, University of Texas Southwestern Medical Center, Dallas, TX, USA.

^113^University Paris Descartes, EA 4468, AP-HP, Geriatrics Department, Hôpital Broca, Paris, France.

^114^Regional Neurogenetic Centre (CRN), ASP Catanzaro, Lamezia Terme, Italy.

^115^Departments of Psychiatry, Medicine, Family & Community Medicine, South Texas Veterans Health Administration Geriatric Research Education & Clinical Center (GRECC), UT Health Science Center at San Antonio, San Antonio, TX, USA.

^116^University of Bordeaux, Inserm 1219, Bordeaux, France.

^117^Department of Neurology, Bordeaux University Hospital / CHU de Bordeaux, Bordeaux, France.

^118^Department of Psychiatry, Icahn School of Medicine at Mount Sinai, New York, NY, USA.

^119^Inserm U1127, CNRS UMR 7225, Sorbonne Universités, UPMC Université Paris 06, UMRS 1127, Institut du Cerveau et de la Moelle Épinière, Paris, France.

^120^AP-HP, Department of Genetics, Pitié-Salpêtrière Hospital, Paris, France.

^121^Section of Gerontology and Geriatrics, Department of Medicine, University of Perugia, Perugia, Italy.

^122^Cambridge Institute for Medical Research, University of Cambridge, Cambridge, UK.

^123^Tanz Centre for Research in Neurodegenerative Disease, University of Toronto, Toronto, Ontario, Canada.

^124^Inserm U1061 Neuropsychiatry, La Colombière Hospital, Montpellier, France.

^125^Montpellier University, Montpellier, France.

^126^Department of Clinical Brain Sciences, University of Edinburgh, Edinburgh, UK.

^127^VA Puget Sound Health Care System/>GRECC, Seattle, WA, USA.

^128^Department of Psychiatry and Behavioral Sciences, University of Washington School of Medicine, Seattle, WA, USA.

^129^Institut de la Mémoire et de la Maladie d'Alzheimer and Institut du Cerveau et de la Moelle Épinière, Département de Neurologie, Hôpital de la Pitié-Salpêtrière, Paris, France.

^130^Institut des Neurosciences Translationnelles de Paris, Institut du Cerveau et de la Moelle Épinière, Paris, France.

^131^Inserm, CNRS, UMR-S975, Institut du Cerveau et de la Moelle Epinière, Paris, France.

^132^Sorbonne Universités, Université Pierre et Marie Curie, Hôpital de la Pitié-Salpêtrière, AP-HP, Paris, France.

^133^Ageing Group, Centre for Public Health, School of Medicine, Dentistry and Biomedical Sciences, Queen's University Belfast, Belfast, UK.

^134^Departments of Neurology, Pharmacology & Neuroscience, Texas Tech University Health Science Center, Lubbock, TX, USA.

^135^Department of Neurology, University of Michigan, Ann Arbor, MI, USA.

^136^Geriatric Research, Education and Clinical Center (GRECC), VA Ann Arbor Healthcare System (VAAAHS), Ann Arbor, MI, USA.

^137^Michigan Alzheimer Disease Center, Ann Arbor, MI, USA.

^138^Aging Research Center, Department of Neurobiology, Care Sciences and Society, Karolinska Institutet and Stockholm University, Stockholm, Sweden.

^139^Indiana Alzheimer's Disease Center, Indiana University School of Medicine, Indianapolis, IN, USA.

^140^Department of Medical and Molecular Genetics, Indiana University, Indianapolis, IN, USA.

^141^Department of Neurology, Indiana University, Indianapolis, IN, USA.

^142^Department of Radiology and Imaging Sciences, Indiana University, Indianapolis, IN, USA.

^143^Department of Psychiatry, University of Pennsylvania Perelman School of Medicine, Philadelphia, PA, USA.

^144^Geriatric Research, Education and Clinical Center (GRECC), University of Wisconsin, Madison, WI, USA.

^145^Department of Medicine, University of Wisconsin, Madison, WI, USA.

^146^Wisconsin Alzheimer's Disease Research Center, Madison, WI, USA.

^147^AXA Research Fund & Sorbonne University Chair, Paris, France.

^148^Sorbonne University, GRC n° 21, Alzheimer Precision Medicine (APM), AP-HP, Pitié-Salpêtrière Hospital, Paris, France.

^149^Brain & Spine Institute, Inserm U 1127, CNRS UMR 7225, Paris, France.

^150^Institute of Memory and Alzheimer's Disease, Department of Neurology, Pitié-Salpêtrière Hospital, AP-HP, Paris, France.

^151^Grupo de Medicina Xenomica, Universidade de Santiago de Compostela, Centro Nacional de Genotipado, Centro de Investigación Biomédica en Red de Enfermedades Raras, Santiago de Compostela, Spain.

^152^UK Dementia Research Institute, University of Cambridge, Cambridge, UK.

^153^Department of Neurological Sciences, Rush University Medical Center, Chicago, IL, USA.

^154^Department of Behavioral Sciences, Rush University Medical Center, Chicago, IL, USA.

^155^Rush Alzheimer's Disease Center, Rush University Medical Center, Chicago, IL, USA.

^156^Central Institute of Mental Health, Medical Faculty Mannheim, University of Heidelberg, Heidelberg, Germany.

^157^Civin Laboratory for Neuropathology, Banner Sun Health Research Institute, Phoenix, AZ, USA.

^158^Department of Neurology, University of Pittsburgh, Pittsburgh, PA, USA.

^159^Department of Psychology, University of Pittsburgh School of Medicine, Pittsburgh, PA, USA.

^160^Department of Pathology, Northwestern University Feinberg School of Medicine, Chicago, IL, USA.

^161^Mesulam Center for Cognitive Neurology and Alzheimer's Disease, Northwestern University Feinberg School of Medicine, Chicago, IL, USA.

^162^Genetic Epidemiology, QIMR Berghofer Medical Research Institute, Herston, Queensland, Australia.

^163^Swedish Medical Center, Seattle, WA, USA.

^164^Department of Neurology, University of California, San Francisco, San Francisco, CA, USA.

^165^Department of Neurology, Duke University, Durham, NC, USA.

^166^Departments of Biology, Brigham Young University, Provo, UT, USA.

^167^University of Kansas Alzheimer's Disease Center, University of Kansas Medical Center, Kansas City, KS, USA.

^168^Department of Experimental and Clinical Medicine, Neurological Institute, University of Pisa, Pisa, Italy.

^169^Department of Genetics and Genomic Sciences, Icahn School of Medicine at Mount Sinai, New York, NY, USA.

^170^Department of Neuroscience, Icahn School of Medicine at Mount Sinai, New York, NY, USA.

^171^Department of Pathology and Immunology, Washington University, St. Louis, MO, USA.

^172^Division of Psychiatry, University College London, London, UK.

^173^USF Health Byrd Alzheimer's Institute, University of South Florida, Tampa, FL, USA.

^174^Fred Hutchinson Cancer Research Center, Seattle, WA, USA.

^175^Department of Molecular Neuroscience, UCL, Institute of Neurology, London, UK.

^176^Mental Health & Behavioral Science Service, Bruce W. Carter VA Medical Center, Miami, FL, USA.

^177^Department of Neurodegenerative Disease, UCL Institute of Neurology, London, UK.

^178^Department of Neuroscience, Mayo Clinic, Jacksonville, FL, USA.

^179^Department of Neurology, University of Southern California, Los Angeles, CA, USA.

^180^Department of Neurology, Catholic University of Rome, Rome, Italy.

^181^Department of Psychiatry and Behavioral Sciences, Miller School of Medicine, University of Miami, Miami, FL, USA.

^182^Department of Neurology, University of California, Davis, Sacramento, CA, USA.

^183^Institute for Memory Impairments and Neurological Disorders, University of California, Irvine, Irvine, CA, USA.

^184^Wien Center for Alzheimer's Disease and Memory Disorders, Mount Sinai Medical Center, Miami Beach, FL, USA.

^185^Rush Institute for Healthy Aging, Department of Internal Medicine, Rush University Medical Center, Chicago, IL, USA.

^186^Department of Old Age Psychiatry, Institute of Psychiatry, Psychology and Neuroscience, King's College London, London, UK.

^187^Department of Primary Medical Care, University Medical Centre Hamburg-Eppendorf, Hamburg, Germany.

^188^Department of Pathology, University of Alabama at Birmingham, Birmingham, AL, USA.

^189^Sanders-Brown Center on Aging, Department of Biostatistics, University of Kentucky, Lexington, KY, USA.

^190^Department of Psychiatry, New York University, New York, NY, USA.

^191^Department of Psychiatry and Psychotherapy, University of Cologne, Cologne, Germany.

^192^Department of Psychiatry and Psychotherapy, Charité University Medicine, Berlin, Germany.

^193^Department of Neurosciences, University of California, San Diego, La Jolla, CA, USA.

^194^Department of Pathology and Laboratory Medicine, Emory University, Atlanta, GA, USA.

^195^Emory Alzheimer's Disease Center, Emory University, Atlanta, GA, USA.

1^96^Department of Psychiatry, University of Freiburg, Freiburg, Germany.

^197^Neurogenetics Program, University of California, Los Angeles, Los Angeles, CA, USA.

^198^Department of Psychiatry, Washington University School of Medicine, St. Louis, MO, USA.

^199^Hope Center Program on Protein Aggregation and Neurodegeneration, Washington University School of Medicine, St. Louis, MO, USA.

^200^Division of Genetics, Department of Medicine and Partners Center for Personalized Genetic Medicine, Brigham and Women's Hospital and Harvard Medical School, Boston, MA, USA.

^201^Department of Neurology, Washington University, St. Louis, MO, USA.

^202^Department of Genetics, Washington University, St. Louis, MO, USA.

^203^Department of Neurology, Massachusetts General Hospital/Harvard Medical School, Boston, MA, USA.

^204^Department of Pathology (Neuropathology), University of Pittsburgh, Pittsburgh, PA, USA.

^205^Department of Neurology, University of Alabama at Birmingham, Birmingham, AL, USA.

^206^Cologne Center for Genomics, University of Cologne, Cologne, Germany.

^207^Department of Pathology, Duke University, Durham, NC, USA.

^208^Department of Genome Sciences, University of Washington, Seattle, WA, USA.

^209^Department of Medicine (Medical Genetics), University of Washington, Seattle, WA, USA.

^210^Sanders-Brown Center on Aging, College of Public Health, Department of Epidemiology, University of Kentucky, Lexington, KY, USA.

^211^Unidad Clínica de Enfermedades Infecciosas y Microbiología, Hospital Universitario de Valme, Sevilla, Spain.

^212^Department of Medicine (Biomedical Genetics), Boston University School of Medicine, Boston, MA, USA.

^213^Functional Genomics Center Zurich, ETH/University of Zurich, Zurich, Switzerland.

^214^Department of Neurology, Oregon Health &Science University, Portland, OR, USA.

^215^Department of Neurology, Portland Veterans Affairs Medical Center, Portland, OR, USA.

^216^Department of Pathology and Laboratory Medicine, University of California, Irvine, Irvine, CA, USA.

^217^Department of Pathology, Boston University School of Medicine, Boston University, Boston, MA, USA.

^218^Inserm U558, University of Toulouse, Toulouse, France.

^219^Department of Neuropsychology, University of California San Francisco, San Francisco, CA, USA.

^220^Institute for Ageing and Health, Newcastle University, Newcastle upon Tyne, UK.

^221^Department of Neurobiology and Behavior, University of California, Irvine, Irvine, CA, USA.

^222^Department of Neurology, Emory University, Atlanta, GA, USA.

^223^Institute for Stroke and Dementia Research, Klinikum der Universität München, Munich, Germany.

^224^German Center for Neurodegenerative Diseases, Munich, Germany.

^225^Cleveland Clinic Lou Ruvo Center for Brain Health, Cleveland Clinic, Cleveland, OH, USA.

^226^Division of Neuroscience and Experimental Psychology, School of Biological Sciences, Faculty of Biology, Medicine and Health, University of Manchester, Manchester Academic Health Science Centre, Manchester, UK.

^227^Department of Pathology, University of Michigan, Ann Arbor, MI, USA.

^228^Institute of Epidemiology, Helmholtz Zentrum München, German Research Center for Environmental Health, Neuherberg, Munich, Germany.

^229^Helmholtz Center Munich, Institute of Epidemiology, Neuherberg, Munich, Germany.

^230^Ludwig-Maximilians University Chair of Epidemiology, Munich, Germany.

^231^Joint Biobank Munich and KORA Biobank, Baltimore, MD, USA.

^232^Department of Psychiatry, Johns Hopkins University, Baltimore, MD, USA.

^233^Human Genetics, Schools of Life Sciences and Medicine, University of Nottingham, Nottingham, UK.

^234^Department of Medicine-Pulmonary, New York University, New York, NY, USA.

^235^Department of Neurology, University of Miami, Miami, FL, USA.

^236^Department of Pathology, University of California, San Diego, La Jolla, CA, USA.

^237^Institute of Neurology, Catholic University of Sacred Hearth, Rome, Italy.

^238^School of Nursing Northwest Research Group on Aging, University of Washington, Seattle, WA, USA.

^239^Institute of Primary Care and Public Health, Cardiff University, University Hospital of Wales, Cardiff, UK.

^240^Department of Neurology, Northwestern University Feinberg School of Medicine, Chicago, IL, USA.

^241^Cambridge Institute of Public Health, University of Cambridge School of Clinical Medicine, Cambridge, UK.

^242^Weill Institute for Neurosciences, Memory and Aging Center, University of California, San Francisco, San Francisco, CA, USA.

^243^Institute of Social Medicine, Occupational Health and Public Health, University of Leipzig, Leipzig, Germany.

^244^Department of Pathology, University of Southern California, Los Angeles, CA, USA.

^245^Department of Pathology and Laboratory Medicine, University of California, Davis, Sacramento, CA, USA.

^246^Institute of Psychiatry, Psychology and Neuroscienceó, King's College London, London, UK.

^247^UK Dementia Research Institute, King's College London, London, UK.

^248^Department of Psychiatry and Psychotherapy, University Medical Center Goettingen, Goettingen, Germany.

^249^German Center for Neurodegenerative Diseases, Goettingen, Germany.

^250^IBiMED, Medical Sciences Department, University of Aveiro, Aveiro, Portugal.

^251^Molecular Genetics Laboratory-Hospital, University of Central Asturias, Oviedo, Spain.

^252^Department of Laboratory Medicine and Pathology, Mayo Clinic, Rochester, MN, USA.

^253^Molecular Genetics Section, Laboratory of Neurogenetics, National Institute on Aging, National Institutes of Health, Bethesda, MD, USA.

^254^Department of Neurology, University of California, Irvine, Irvine, CA, USA.

^255^Department of Neuroscience, Psychology, Drug Research and Child Health, University of Florence, Florence, Italy.

^256^Centro di Ricerca, Trasferimento e Alta Formazione DENOTHE, University of Florence, Florence, Italy.

^257^Department of Neurology, University of Colorado School of Medicine, Aurora, CO, USA.

^258^IRCCS Fondazione Don Carlo Gnocchi, Florence, Italy.

^259^Laboratory of Neuropsychiatry, IRCCS Santa Lucia Foundation, Rome, Italy.

^260^Division of Neuropsychiatry, Department of Psychiatry and Behavioral Sciences, Baylor College of Medicine, Houston, TX, USA.

^261^Alzheimer's Disease Center, New York University, New York, NY, USA.

^262^Oxford Healthy Aging Project, Clinical Trial Service Unit, University of Oxford, Oxford, UK.

^263^Department of Epidemiology, Columbia University, New York, NY, USA.

^264^Oxford Project to Investigate Memory and Ageing, University of Oxford, Nuffield Department of Clinical Neurosciences, John Radcliffe Hospital, Oxford, UK.

^265^Department of Neurology, Keck School of Medicine at the University of Southern California, Los Angeles, Los Angeles, CA, USA.

^266^Department of Neurology, University of Texas Southwestern Medical Center, Dallas, TX, USA.

^267^Population Health Sciences, Bristol Medical School, University of Bristol, Bristol, UK.

^268^Department of Pathology (Neuropathology), Rush University Medical Center, Chicago, IL, USA.

^269^Department of Psychiatry, University of Southern California, Los Angeles, CA, USA.

^270^Department of Pathology and Laboratory Medicine, University of Pennsylvania Perelman School of Medicine, Philadelphia, PA, USA.

^271^Department of Pathology, Johns Hopkins University, Baltimore, MD, USA.

^272^Sanders-Brown Center on Aging, Department of Neuroscience, University of Kentucky, Lexington, KY, USA.

^273^Department of Neurology, University of California, Los Angeles, Los Angeles, CA, USA.

^274^Department of Pathology and Laboratory Medicine, University of California, Los Angeles, Los Angeles, CA, USA.

^275^Taub Institute on Alzheimer's Disease and the Aging Brain, Department of Pathology, Columbia University, New York, NY, USA.

^276^Department of Psychiatry and Behavioral Sciences, Northwestern University Feinberg School of Medicine, Chicago, IL, USA.

^277^Department of Psychiatry and Behavioral Sciences, Duke University, Durham, NC, USA.

^278^Department of Genetics, University of North Carolina at Chapel Hill, Chapel Hill, NC, USA.

^279^Department of Human Genetics, Emory University, Atlanta, GA, USA.

^280^Department of Pathology, Oregon Health & Science University, Portland, OR, USA.

^281^National Institute of Neurological Disorders and Stroke, Bethesda, MD, USA.

^282^Gottfried Schatz Research Center for Cell Signaling, Metabolism and Aging, Division of Molecular Biology and Biochemistry, Medical University Graz, Graz, Austria.

^283^Gerontology and Geriatrics Research Laboratory, IRCCS Casa Sollievo della Sofferenza, San Giovanni Rotondo, Italy.

^284^Department Geriatric Care, Orthogeriatrics and Rehabilitation, Galliera Hospital, Genova, Italy.

^285^IdiPAZ, Instituto de Investigación Sanitaria la Paz, Madrid, Spain.

^286^Centro de Biologia Molecular Severo Ochoa (CSIC-UAM), Madrid, Spain.

^287^German Center for Neurodegenerative Diseases, Berlin, Germany.

^288^Instituto di Ricovero e Cura a Carattere Scientifico, Associazione Oasi Maria Santissima Srl, Troina, Italy.

^289^Center for Translational and Computational Neuroimmunology, Department of Neurology, Columbia University Medical Center, New York, NY, USA.

^290^Memory Unit, Neurology Department and Sant Pau Biomedical Research Institute, Hospital de la Santa Creu i Sant Pau, Autonomous University Barcelona, Barcelona, Spain.

^291^Somerset Partnership NHS Trust, Somerset, UK.

^292^The Wellcome Trust Sanger Institute, Hinxton, Cambridge, UK.

^293^Ronald M. Loeb Center for Alzheimer's Disease, Department of Neuroscience, Icahn School of Medicine at Mount Sinai, New York, NY, USA.

^294^University of Bristol Medical School, Learning & Research level 2, Southmead Hospital, Bristol, UK.

^295^Department of Neurology, Mayo Clinic, Jacksonville, FL, USA.

^296^Memory Research and Resources Center, CMRR de Bordeaux, Bordeaux, France.

^297^Utah State University, Logan, UT, USA.

^298^Department of Neurology, Erasmus MC University Medical Center, Rotterdam, the Netherlands.

^299^Departments of Radiology, Erasmus MC University Medical Center, Rotterdam, the Netherlands.

^300^Department of Research Rouvray Psychiatric Hospital, Sotteville-lès-Rouen, France.

^301^Department of Neurology, Medical University Graz, Graz, Austria.

^302^Center for Applied Genomics, Children's Hospital of Philadelphia, The Perelman School of Medicine, University of Pennsylvania, Philadelphia, PA, USA.

^303^Division of Human Genetics, Department of Pediatrics, The Perelman School of Medicine, University of Pennsylvania, Philadelphia, PA, USA.

^304^Department of Ophthalmology, Boston University School of Medicine, Boston University, Boston, MA, USA.

^305^Department of Epidemiology, Boston University School of Public Health, Boston, MA, USA.

^306^Department of Health Services, University of Washington, Seattle, WA, USA.

^307^Kaiser Permanente, Washington Health Research Institute, Seattle, WA, USA.

^308^Glenn Biggs Institute for Alzheimer's and Neurodegenerative Diseases, San Antonio, TX, USA.

^309^Centre Hospitalier Universitaire de Lille, Lille, France.

^310^Inserm, U1167, RID-AGE-Risk Factors and Molecular Determinants of Aging-Related Diseases, Lille, France. jean-charles.lambert@pasteur-lille.fr.

^311^Institut Pasteur de Lille, Lille, France. jean-charles.lambert@pasteur-lille.fr.

^312^Univ. Lille, U1167-Excellence Laboratory LabEx DISTALZ, Lille, France. jean-charles.lambert@pasteur-lille.fr.

^313^John P. Hussman Institute for Human Genomics, University of Miami Miller School of Medicine, Miami, FL, USA. mpericak@miami.edu.

**3. Identification of novel risk loci, causal insights, and heritable risk for Parkinson's disease: a meta-analysis of genome-wide association studies.**

Mike A Nalls^1^, Cornelis Blauwendraat^2^, Costanza L Vallerga^3^, Karl Heilbron^4^, Sara Bandres-Ciga^2^, Diana Chang^5^, Manuela Tan^6^, Demis A Kia^6^, Alastair J Noyce ^7^, Angli Xue^8^, Jose Bras ^9^, Emily Young^10^, Rainer von Coelln^11^, Javier Simón-Sánchez^12^, Claudia Schulte^12^, Manu Sharma^13^, Lynne Krohn^14^, Lasse Pihlstrøm^15^, Ari Siitonen^16^, Hirotaka Iwaki^17^, Hampton Leonard^18^, Faraz Faghri^19^, J Raphael Gibbs^2^, Dena G Hernandez^2^, Sonja W Scholz^20^, Juan A Botia^21^, Maria Martinez^22^, Jean-Christophe Corvol^23^, Suzanne Lesage^23^, Joseph Jankovic^10^, Lisa M Shulman^11^, Margaret Sutherland^24^, Pentti Tienari^25^, Kari Majamaa^16^, Mathias Toft^26^, Ole A Andreassen^27^, Tushar Bangale^5^, Alexis Brice^23^, Jian Yang^8^, Ziv Gan-Or^28^, Thomas Gasser^12^, Peter Heutink^12^, Joshua M Shulman^29^, Nicholas W Wood^6^, David A Hinds^4^, John A Hardy^30^, Huw R Morris^31^, Jacob Gratten^32^, Peter M Visscher^8^, Robert R Graham^5^, Andrew B Singleton^2^, 23andMe Research Team; System Genomics of Parkinson's Disease Consortium; International Parkinson's Disease Genomics Consortium

^1^Laboratory of Neurogenetics, National Institute on Aging, National Institutes of Health, Bethesda, MD, USA; Data Tecnica International, Glen Echo, MD, USA. Electronic address: mike@datatecnica.com.

^2^Laboratory of Neurogenetics, National Institute on Aging, National Institutes of Health, Bethesda, MD, USA.

^3^Institute for Molecular Bioscience, The University of Queensland, Brisbane, QLD, Australia.

^4^23andMe, Sunnyvale, CA, USA.

^5^Department of Human Genetics, Genentech, South San Francisco, CA, USA.

^6^Department of Molecular Neuroscience, UCL Queen Square Institute of Neurology, London, UK; Department of Clinical and Movement Neuroscience and UCL Movement Disorders Centre, UCL Queen Square Institute of Neurology, London, UK.

^7^Department of Molecular Neuroscience, UCL Queen Square Institute of Neurology, London, UK; Preventive Neurology Unit, Wolfson Institute of Preventive Medicine, Queen Mary University of London, London, UK.

^8^Queensland Brain Institute, The University of Queensland, Brisbane, QLD, Australia; Institute for Molecular Bioscience, The University of Queensland, Brisbane, QLD, Australia.

^9^Department of Neurodegenerative Diseases, UCL Queen Square Institute of Neurology, London, UK; Center for Neurodegenerative Science, Van Andel Research Institute, Grand Rapids, MI, USA.

^10^Department of Neurology, Baylor College of Medicine, Houston, TX, USA.

^11^Department of Neurology, University of Maryland School of Medicine, Baltimore, MD, USA.

^12^Department for Neurodegenerative Diseases, Hertie Institute for Clinical Brain Research, University of Tübingen, Tübingen, Germany; German Center for Neurodegenerative Diseases, Tübingen, Germany.

^13^Centre for Genetic Epidemiology, Institute for Clinical Epidemiology and Applied Biometry, University of Tübingen, Tübingen, Germany.

^14^Department of Human Genetics, McGill University, Montreal, QC, Canada; Montreal Neurological Institute, McGill University, Montreal, QC, Canada.

^15^Department of Neurology, Oslo University Hospital, Oslo, Norway.

^16^Institute of Clinical Medicine, Department of Neurology, University of Oulu, Oulu, Finland; Department of Neurology and Medical Research Center, Oulu University Hospital, Oulu, Finland.

^17^Laboratory of Neurogenetics, National Institute on Aging, National Institutes of Health, Bethesda, MD, USA; Data Tecnica International, Glen Echo, MD, USA; The Michael J Fox Foundation, New York, NY, USA.

^18^Laboratory of Neurogenetics, National Institute on Aging, National Institutes of Health, Bethesda, MD, USA; Data Tecnica International, Glen Echo, MD, USA.

^19^Laboratory of Neurogenetics, National Institute on Aging, National Institutes of Health, Bethesda, MD, USA; Department of Computer Science, University of Illinois Urbana-Champaign, Champaign, IL, USA.

^20^National Institute of Neurological Disorders and Stroke, National Institutes of Health, Bethesda, MD, USA; Department of Neurology, Johns Hopkins University Medical Center, Baltimore, MD, USA.

^21^Department of Molecular Neuroscience, UCL Queen Square Institute of Neurology, London, UK; Departamento de Ingeniería de la Información y las Comunicaciones, Universidad de Murcia, Spain.

^22^Institut national de la santé et de la recherche médicale Unité mixte de recherche 1220, Toulouse, France; Paul Sabatier University, Toulouse, France.

^23^Institut national de la santé et de la recherche médicale U1127, CNRS UMR 7225, Paris, France; Sorbonne Université centre national de la recherche médicale, unité mixte de recherche 1127, Paris, France; Assistance Publique Hôpitaux de Paris, Paris, France; Institut du Cerveau et de la Moelle épinière, Paris, France.

^24^National Institute of Neurological Disorders and Stroke, National Institutes of Health, Bethesda, MD, USA.

^25^Clinical Neurosciences, Neurology, University of Helsinki, Helsinki, Finland; Helsinki University Hospital, Helsinki, Finland.

^26^Department of Neurology, Oslo University Hospital, Oslo, Norway; Institute of Clinical Medicine, University of Oslo, Oslo, Norway.

^27^Division of Mental Health and Addiction, Oslo University Hospital, Oslo, Norway; Jebsen Centre for Psychosis Research, University of Oslo, Oslo, Norway.

^28^Department of Human Genetics, McGill University, Montreal, QC, Canada; Montreal Neurological Institute, McGill University, Montreal, QC, Canada; Department of Neurology and Neurosurgery, McGill University, Montreal, QC, Canada.

^29^Department of Neurology, Baylor College of Medicine, Houston, TX, USA; Department of Molecular and Human Genetics, Baylor College of Medicine, Houston, TX, USA; Department of Neuroscience, Baylor College of Medicine, Houston, TX, USA; Jan and Dan Duncan Neurological Research Institute, Texas Children's Hospital, Houston, TX, USA.

^30^Department of Molecular Neuroscience, UCL Queen Square Institute of Neurology, London, UK.

^31^Department of Clinical and Movement Neuroscience and UCL Movement Disorders Centre, UCL Queen Square Institute of Neurology, London, UK.

^32^Institute for Molecular Bioscience, The University of Queensland, Brisbane, QLD, Australia; Mater Research Institute, The University of Queensland, Brisbane, QLD, Australia.

**4. Genome-wide Analyses Identify KIF5A as a Novel ALS Gene**

Aude Nicolas ^1^, Kevin P Kenna ^2^, Alan E Renton ^3^, Nicola Ticozzi ^4^, Faraz Faghri ^5^, Ruth Chia ^1^, Janice A Dominov ^2^, Brendan J Kenna ^2^, Mike A Nalls ^6^, Pamela Keagle ^2^, Alberto M Rivera 1, Wouter van Rheenen 7, Natalie A Murphy 1, Joke J F A van Vugt 7, Joshua T Geiger ^8^, Rick A Van der Spek ^7^, Hannah A Pliner ^1^, Shankaracharya ^2^, Bradley N Smith ^9^, Giuseppe Marangi ^10^, Simon D Topp ^9^, Yevgeniya Abramzon ^11^, Athina Soragia Gkazi ^9^, John D Eicher ^12^, Aoife Kenna ^2^, ITALSGEN Consortium; Gabriele Mora ^13^, Andrea Calvo ^14^, Letizia Mazzini ^15^, Nilo Riva ^16^, Jessica Mandrioli ^17^, Claudia Caponnetto ^18^, Stefania Battistini ^19^, Paolo Volanti ^13^, Vincenzo La Bella ^20^, Francesca L Conforti ^21^, Giuseppe Borghero ^22^, Sonia Messina ^23^, Isabella L Simone ^24^, Francesca Trojsi ^25^, Fabrizio Salvi ^26^, Francesco O Logullo ^27^, Sandra D'Alfonso ^28^, Lucia Corrado ^28^, Margherita Capasso ^29^, Luigi Ferrucci ^30^, Genomic Translation for ALS Care (GTAC) Consortium; Cristiane de Araujo Martins Moreno ^31^, Sitharthan Kamalakaran ^32^, David B Goldstein ^32^, ALS Sequencing Consortium; Aaron D Gitler ^33^, Tim Harris ^34^, Richard M Myers ^35^, NYGC ALS Consortium; Hemali Phatnani ^36^, Rajeeva Lochan Musunuri ^37^, Uday Shankar Evani ^37^, Avinash Abhyankar ^37^, Michael C Zody ^37^, Answer ALS Foundation; Julia Kaye ^38^, Steven Finkbeiner ^39^, Stacia K Wyman ^38^, Alex LeNail ^40^, Leandro Lima ^38^, Ernest Fraenkel ^41^, Clive N Svendsen ^42^, Leslie M Thompson ^43^, Jennifer E Van Eyk ^44^, James D Berry ^45^, Timothy M Miller ^46^, Stephen J Kolb ^47^, Merit Cudkowicz ^45^, Emily Baxi ^48^, Clinical Research in ALS and Related Disorders for Therapeutic Development (CReATe) Consortium; Michael Benatar ^49^, J Paul Taylor ^50^, Evadnie Rampersaud ^51^, Gang Wu ^51^, Joanne Wuu ^49^, SLAGEN Consortium; Giuseppe Lauria 52, Federico Verde 53, Isabella Fogh 54, Cinzia Tiloca ^53^, Giacomo P Comi ^55^, Gianni Sorarù ^56^, Cristina Cereda ^57^, French ALS Consortium; Philippe Corcia ^58^, Hannu Laaksovirta ^59^, Liisa Myllykangas ^60^, Lilja Jansson ^59^, Miko Valori ^59^, John Ealing ^61^, Hisham Hamdalla ^61^, Sara Rollinson ^62^, Stuart Pickering-Brown ^62^, Richard W Orrell ^63^, Katie C Sidle ^64^, Andrea Malaspina ^65^, John Hardy ^64^, Andrew B Singleton ^66^, Janel O Johnson ^1^, Sampath Arepalli ^67^, Peter C Sapp ^2^, Diane McKenna-Yasek ^2^, Meraida Polak ^68^, Seneshaw Asress ^68^, Safa Al-Sarraj ^9^, Andrew King ^9^, Claire Troakes ^9^, Caroline Vance ^9^, Jacqueline de Belleroche ^69^, Frank Baas ^70^, Anneloor L M A Ten Asbroek ^71^, José Luis Muñoz-Blanco ^72^, Dena G Hernandez ^67^, Jinhui Ding ^73^, J Raphael Gibbs ^73^, Sonja W Scholz 74, Mary Kay Floeter ^75^, Roy H Campbell ^76^, Francesco Landi ^77^, Robert Bowser ^78^, Stefan M Pulst ^79^, John M Ravits ^80^, Daniel J L MacGowan ^81^, Janine Kirby ^82^, Erik P Pioro ^83^, Roger Pamphlett ^84^, James Broach ^85^, Glenn Gerhard ^86^, Travis L Dunckley ^87^, Christopher B Brady ^88^, Neil W Kowall ^89^, Juan C Troncoso ^90^, Isabelle Le Ber ^91^, Kevin Mouzat ^92^, Serge Lumbroso ^92^, Terry D Heiman-Patterson ^93^, Freya Kamel ^94^, Ludo Van Den Bosch ^95^, Robert H Baloh ^96^, Tim M Strom ^97^, Thomas Meitinger ^98^, Aleksey Shatunov ^9^, Kristel R Van Eijk ^7^, Mamede de Carvalho ^99^, Maarten Kooyman ^100^, Bas Middelkoop ^7^, Matthieu Moisse ^95^, Russell L McLaughlin ^101^, Michael A Van Es ^7^, Markus Weber ^102^, Kevin B Boylan ^103^, Marka Van Blitterswijk ^104^, Rosa Rademakers ^104^, Karen E Morrison ^105^, A Nazli Basak ^106^, Jesús S Mora ^107^, Vivian E Drory ^108^, Pamela J Shaw ^82^, Martin R Turner ^109^, Kevin Talbot ^109^, Orla Hardiman ^110^, Kelly L Williams ^111^, Jennifer A Fifita ^111^, Garth A Nicholson ^112^, Ian P Blair ^111^, Guy A Rouleau ^113^, Jesús Esteban-Pérez ^114^, Alberto García-Redondo ^114^, Ammar Al-Chalabi ^9^, Project MinE ALS Sequencing Consortium; Ekaterina Rogaeva ^115^, Lorne Zinman ^116^, Lyle W Ostrow ^48^, Nicholas J Maragakis ^48^, Jeffrey D Rothstein ^48^, Zachary Simmons ^117^, Johnathan Cooper-Knock ^82^, Alexis Brice ^91^, Stephen A Goutman ^118^, Eva L Feldman ^118^, Summer B Gibson ^79^, Franco Taroni ^119^, Antonia Ratti ^4^, Cinzia Gellera ^119^, Philip Van Damme ^12^0, Wim Robberecht ^120^, Pietro Fratta ^121^, Mario Sabatelli ^122^, Christian Lunetta ^123^, Albert C Ludolph ^124^, Peter M Andersen ^125^, Jochen H Weishaupt ^124^, William Camu ^126^, John Q Trojanowski ^127^, Vivianna M Van Deerlin ^127^, Robert H Brown Jr ^2^, Leonard H van den Berg ^7^, Jan H Veldink ^7^, Matthew B Harms ^31^, Jonathan D Glass ^68^, David J Stone ^128^, Pentti Tienari ^59^, Vincenzo Silani ^4^, Adriano Chiò ^129^, Christopher E Shaw ^9^, Bryan J Traynor ^130^, John E Landers ^131^

^1^Neuromuscular Diseases Research Section, Laboratory of Neurogenetics, National Institute on Aging, NIH, Porter Neuroscience Research Center, Bethesda, MD 20892, USA.

^2^Department of Neurology, University of Massachusetts Medical School, Worcester, MA 01605, USA.

^3^Neuromuscular Diseases Research Section, Laboratory of Neurogenetics, National Institute on Aging, NIH, Porter Neuroscience Research Center, Bethesda, MD 20892, USA; Department of Neuroscience, Icahn School of Medicine at Mount Sinai, New York, NY 10029, USA; Ronald M. Loeb Center for Alzheimer's Disease, Icahn School of Medicine at Mount Sinai, New York, NY 10029, USA.

^4^Department of Neurology and Laboratory of Neuroscience, IRCCS Istituto Auxologico Italiano, Milan, Italy; Department of Pathophysiology and Transplantation, "Dino Ferrari" Center - Università degli Studi di Milano, Milan 20122, Italy.

^5^Molecular Genetics Section, Laboratory of Neurogenetics, National Institute on Aging, NIH, Porter Neuroscience Research Center, Bethesda, MD 20892, USA; Department of Computer Science, University of Illinois at Urbana-Champaign, Urbana, IL, USA.

^6^Molecular Genetics Section, Laboratory of Neurogenetics, National Institute on Aging, NIH, Porter Neuroscience Research Center, Bethesda, MD 20892, USA; Data Tecnica International, Glen Echo, MD, USA.

^7^Department of Neurology, Brain Center Rudolf Magnus, University Medical Center Utrecht, Utrecht, the Netherlands.

^8^Neurodegenerative Diseases Research Unit, National Institute of Neurological Disorders and Stroke, NIH, Bethesda, MD 20892, USA.

^9^Maurice Wohl Clinical Neuroscience Institute, Department of Basic and Clinical Neuroscience, King's College London, London SE5 9RS, UK.

^10^Neuromuscular Diseases Research Section, Laboratory of Neurogenetics, National Institute on Aging, NIH, Porter Neuroscience Research Center, Bethesda, MD 20892, USA; Institute of Genomic Medicine, Catholic University, Roma, Italy.

^11^Neuromuscular Diseases Research Section, Laboratory of Neurogenetics, National Institute on Aging, NIH, Porter Neuroscience Research Center, Bethesda, MD 20892, USA; Sobell Department of Motor Neuroscience and Movement Disorders, University College London, Institute of Neurology, London, UK.

^12^Genetics and Pharmacogenomics, MRL, Merck & Co., Inc., Boston, MA 02115, USA.

^13^ALS Center, Salvatore Maugeri Foundation, IRCCS, Mistretta, Messina, Italy.

^14^"Rita Levi Montalcini" Department of Neuroscience, University of Turin, Turin, Italy.

^15^"Maggiore della Carità" University Hospital, Novara, Italy.

^16^Department of Neurology, Institute of Experimental Neurology, Division of Neuroscience, San Raffaele Scientific Institute, Milan, Italy.

^17^Department of Neuroscience, St. Agostino Estense Hospital, Azienda Ospedaliero Universitaria di Modena, Modena, Italy.

^18^Department of Neurosciences, Ophthalmology, Genetics, Rehabilitation, Maternal and Child Health, Ospedale Policlinico San Martino, Genoa, Italy.

^19^Department of Medical, Surgical and Neurological Sciences, University of Siena, Siena, Italy.

20ALS Clinical Research Center, University of Palermo, Palermo, Italy.

^21^Institute of Neurological Sciences, National Research Council, Mangone, Cosenza, Italy.

^22^Department of Neurology, Azienda Universitario Ospedaliera di Cagliari and University of Cagliari, Cagliari, Italy.

^23^Department of Clinical and Experimental Medicine, University of Messina and Nemo Sud Clinical Center for Neuromuscular Diseases, Aurora Foundation, Messina, Italy.

^24^Department of Basic Medical Sciences, Neurosciences and Sense Organs, University of Bari, Bari, Italy.

^25^Department of Medical, Surgical, Neurological, Metabolic and Aging Sciences, University of Campania "Luigi Vanvitelli," Naples, Italy.

^26^"Il Bene" Center for Immunological and Rare Neurological Diseases at Bellaria Hospital, IRCCS, Istituto delle Scienze Neurologiche, Bologna, Italy.

^27^Neurological Clinic, Marche Polytechnic University, Ancona, Italy.

^28^Department of Health Sciences, University of Eastern Piedmont, Novara, Italy.

^29^Department of Neurology, University of Chieti, Chieti, Italy.

^30^Longitudinal Studies Section, Clinical Research Branch, National Institute on Aging, NIH, Baltimore, MD 21224, USA.

^31^Department of Neurology, Columbia University, New York, NY 10032, USA.

^32^Institute for Genomic Medicine, Columbia University, New York, NY 10032, USA.

^33^Department of Genetics, Stanford University School of Medicine, Stanford, CA 94305, USA.

^34^Bioverativ, 225 2nd Avenue, Waltham, MA 02145, USA.

^35^HudsonAlpha Institute for Biotechnology, Huntsville, AL 35806, USA.

^36^Center for Genomics of Neurodegenerative Diseases (CGND), New York Genome Center, New York, NY, USA.

^37^Computational Biology, New York Genome Center, New York, NY, USA.

^38^Gladstone Institute of Neurological Disease, San Francisco, CA, USA.

^39^Gladstone Institute of Neurological Disease, San Francisco, CA, USA; Departments of Neurology and Physiology, University of California, San Francisco, San Francisco, CA, USA.

^40^Department of Biological Engineering, Massachusetts Institute of Technology, 77 Massachusetts Avenue, Cambridge, MA 02139, USA.

^41^Department of Biological Engineering, Massachusetts Institute of Technology, 77 Massachusetts Avenue, Cambridge, MA 02139, USA; Broad Institute, 415 Main Street, Cambridge, MA 02142, USA.

^42^Board of Governors Regenerative Medicine Institute, Cedars-Sinai Medical Center, Los Angeles, CA 90048, USA; Department of Biomedical Sciences, Cedars-Sinai Medical Center, Los Angeles, CA 90048, USA.

^43^Department of Neurobiology and Behavior, Institute of Memory Impairment and Neurological Disorders, University of California, Irvine, Irvine, CA 92697, USA; Department of Psychiatry and Human Behavior, Institute of Memory Impairment and Neurological Disorders, University of California, Irvine, Irvine, CA 92697, USA.

^44^The Heart Institute and Department of Medicine, Cedars-Sinai Medical Center, Los Angeles, CA, USA.

^45^Harvard Medical School, Department of Neurology, Massachusetts General Hospital (MGH), Boston, MA, USA; Neurological Clinical Research Institute (NCRI), Massachusetts General Hospital, Boston, MA, USA.

^46^Department of Neurology, Washington University School of Medicine, St. Louis, MO, USA.

^47^Department of Neurology, The Ohio State University Wexner Medical Center, Columbus, OH, USA.

^48^Department of Neurology, Johns Hopkins University, Baltimore, MD 21287, USA.

^49^Department of Neurology, University of Miami, Miami, FL 33136, USA.

^50^Howard Hughes Medical Institute, Chevy Chase, MD 20815, USA; Department of Cell and Molecular Biology, St. Jude Children's Research Hospital, Memphis, TN 38105, USA.

^51^Department of Computational Biology, St. Jude Children's Research Hospital, Memphis, TN 38105, USA.

^52^3rd Neurology Unit, Motor Neuron Diseases Center, Fondazione IRCCS Istituto Neurologico "Carlo Besta," and Department of Biomedical and Clinical Sciences "Luigi Sacco," University of Milan, Milan, Italy.

^53^Department of Neurology and Laboratory of Neuroscience, IRCCS Istituto Auxologico Italiano, Milan, Italy.

^54^Department of Neurology and Laboratory of Neuroscience, IRCCS Istituto Auxologico Italiano, Milan, Italy; Maurice Wohl Clinical Neuroscience Institute, Department of Basic and Clinical Neuroscience, King's College London, London SE5 9RS, UK.

^55^Neurology Unit, IRCCS Foundation Ca' Granda Ospedale Maggiore Policlinico, Milan, Italy.

^56^Department of Neurosciences, University of Padova, Padova, Italy.

^57^Genomic and Post-Genomic Center, IRCCS Mondino Foundation, Pavia, Italy.

^58^ALS Center, CHU Bretonneau, Tours University, Tours, France.

^59^Department of Neurology, Helsinki University Hospital and Molecular Neurology Programme, Biomedicum, University of Helsinki, Helsinki FIN-02900, Finland.

^60^Department of Pathology, University of Helsinki and Helsinki University Hospital, Helsinki, Finland.

^61^Greater Manchester Neurosciences Centre, Salford Royal NHS Foundation Trust, Salford M6 8HD, UK.

^62^Faculty of Human and Medical Sciences, University of Manchester, Manchester M13 9PT, UK.

^63^Department of Clinical Neuroscience, Institute of Neurology, University College London, London NW3 2PG, UK.

^64^Department of Molecular Neuroscience and Reta Lila Weston Laboratories, Institute of Neurology, University College London, Queen Square House, London WC1N 3BG, UK.

^65^Centre for Neuroscience and Trauma, Blizard Institute, Queen Mary University of London, NorthEast London and Essex Regional Motor Neuron Disease Care Centre, London E1 2AT, UK.

^66^Molecular Genetics Section, Laboratory of Neurogenetics, National Institute on Aging, NIH, Porter Neuroscience Research Center, Bethesda, MD 20892, USA.

^67^Genomics Technology Group, Laboratory of Neurogenetics, National Institute on Aging, NIH, Porter Neuroscience Research Center, Bethesda, MD 20892, USA.

^68^Department of Neurology, Emory University School of Medicine, Atlanta, GA 30322, USA.

^69^Division of Brain Sciences, Department of Medicine, Imperial College London, London W120NN, UK.

^70^Department of Clinical Genetics, Leiden University Medical Center, Leiden, the Netherlands.

^71^Department of Neurogenetics and Neurology, Academic Medical Centre, Amsterdam, the Netherlands.

^72^ALS-Neuromuscular Unit, Hospital General Universitario Gregorio Marañón, IISGM, Madrid, Spain.

^73^Computational Biology Group, Laboratory of Neurogenetics, National Institute on Aging, NIH, Porter Neuroscience Research Center, Bethesda, MD 20892, USA.

^74^Neurodegenerative Diseases Research Unit, National Institute of Neurological Disorders and Stroke, NIH, Bethesda, MD 20892, USA; Department of Neurology, Johns Hopkins University, Baltimore, MD 21287, USA.

^75^Motor Neuron Disorders Unit, National Institute of Neurological Disorders and Stroke, NIH, Bethesda, MD 20892, USA.

^76^Department of Computer Science, University of Illinois at Urbana-Champaign, Urbana, IL, USA.

^77^Center for Geriatric Medicine, Department of Geriatrics, Neurosciences and Orthopedics, Catholic University of Sacred Heart, Rome 00168, Italy.

^78^Division of Neurology, Barrow Neurological Institute, Phoenix, AZ, USA.

^79^Department of Neurology, University of Utah School of Medicine, Salt Lake City, UT, USA.

^80^Department of Neuroscience, University of California, San Diego, La Jolla, CA, USA.

^81^Mount Sinai Beth Israel Hospital, Mount Sinai School of Medicine, New York, NY, USA.

^82^Sheffield Institute for Translational Neuroscience (SITraN), University of Sheffield, Sheffield, UK.

^83^Department of Neurology, Neuromuscular Center, Neurological Institute, Cleveland Clinic, Cleveland, OH, USA.

^84^Discipline of Pathology, Brain and Mind Centre, The University of Sydney, 94 Mallett Street, Camperdown, NSW 2050, Australia.

^85^Department of Biochemistry, Penn State College of Medicine, Hershey, PA, USA.

^86^Department of Pathology, Penn State College of Medicine, Hershey, PA, USA.

^87^Neurogenomics Division, Translational Genomics Research Institute, Phoenix, AZ, USA.

^88^Research and Development Service, Veterans Affairs Boston Healthcare System, Boston, MA, USA; Department of Neurology, Program in Behavioral Neuroscience, Boston University School of Medicine, Boston, MA, USA.

^89^Neurology Service, VA Boston Healthcare System and Boston University Alzheimer's Disease Center, Boston, MA 02130, USA.

^90^Departments of Pathology and Neurology, Johns Hopkins University School of Medicine, Baltimore, MD 21205, USA.

^91^Sorbonne Universités, UPMC Univ Paris 06, Inserm, CNRS, Institut du Cerveau et la Moelle (ICM), Assistance Publique Hôpitaux de Paris (AP-HP) - Hôpital Pitié-Salpêtrière, Paris, France.

^92^INM, University Montpellier, Montpellier, France; Department of Biochemistry, CHU Nîmes, Nîmes, France.

^93^Department of Neurology, Drexel University College of Medicine, Philadelphia, PA, USA; Department of Neurology, Lewis Katz School of Medicine, Temple University, Philadelphia, PA, USA.

^94^Epidemiology Branch, National Institute of Environmental Health Sciences, Durham, NC 27709, USA.

^95^KU Leuven - University of Leuven, Department of Neurosciences, Experimental Neurology and Leuven Research Institute for Neuroscience and Disease (LIND), B-3000 Leuven, Belgium; VIB, Center for Brain and Disease Research, Laboratory of Neurobiology, Leuven, Belgium.

^96^Department of Neurology, Cedars-Sinai Medical Center, Los Angeles, CA, USA.

^97^Institute of Human Genetics, Technische Universität München, Munich, Germany; Institute of Human Genetics, Helmholtz Zentrum München, German Research Center for Environmental Health, Neuherberg, Germany.

^98^Institute of Human Genetics, Technische Universität München, Munich, Germany; Institute of Human Genetics, Helmholtz Zentrum München, German Research Center for Environmental Health, Neuherberg, Germany; Munich Cluster for Systems Neurology (SyNergy), Munich, Germany.

^99^Institute of Physiology, Institute of Molecular Medicine, Faculty of Medicine, University of Lisbon, Lisbon, Portugal; Department of Neurosciences, Hospital de Santa Maria-CHLN, Lisbon, Portugal.

^100^SURFsara, Amsterdam, the Netherlands.

^101^Population Genetics Laboratory, Smurfit Institute of Genetics, Trinity College Dublin, Dublin, Republic of Ireland.

^102^Neuromuscular Diseases Center/ALS Clinic, Kantonsspital St. Gallen, St. Gallen, Switzerland.

^103^Department of Neurology, Mayo Clinic Florida, Jacksonville, FL 32224, USA.

^104^Department of Neuroscience, Mayo Clinic, Jacksonville, FL, USA.

^105^Faculty of Medicine, University of Southampton, Southampton, UK.

^106^Suna and Inan Kırac Foundation, Neurodegeneration Research Laboratory, Bogazici University, Istanbul, Turkey.

^107^ALS Unit/Neurology, Hospital San Rafael, Madrid, Spain.

^108^Department of Neurology, Tel-Aviv Sourasky Medical Centre, Tel-Aviv, Israel.

^109^Nuffield Department of Clinical Neurosciences, University of Oxford, Oxford, UK.

^110^Academic Unit of Neurology, Trinity Biomedical Sciences Institute, Trinity College Dublin, Dublin, Republic of Ireland.

^111^Centre for MND Research, Faculty of Medicine and Health Sciences, Macquarie University, Sydney, NSW 2109, Australia.

^112^Centre for MND Research, Faculty of Medicine and Health Sciences, Macquarie University, Sydney, NSW 2109, Australia; ANZAC Research Institute, Concord Hospital, University of Sydney, Sydney, NSW 2139, Australia.

^113^Montreal Neurological Institute, Department of Neurology and Neurosurgery, McGill University, Montreal, QC, Canada.

^114^Unidad de ELA, Instituto de Investigación Hospital 12 de Octubre de Madrid, SERMAS, and Centro de Investigación Biomédica en Red de Enfermedades Raras (CIBERER U-723), Madrid, Spain.

^115^Tanz Centre for Research of Neurodegenerative Diseases, Division of Neurology, Department of Medicine, University of Toronto, Toronto, ON M5S 3H2, Canada.

^116^Division of Neurology, Department of Internal Medicine, Sunnybrook Health Sciences Centre, University of Toronto, Toronto, ON M4N 3M5, Canada.

^117^Department of Neurology, Penn State Hershey Medical Center, Hershey, PA, USA.

^118^Department of Neurology, University of Michigan, Ann Arbor, MI, USA.

^119^Unit of Genetics of Neurodegenerative and Metabolic Diseases, Fondazione IRCCS Istituto Neurologico "Carlo Besta," Milan 20133, Italy.

^120^KU Leuven - University of Leuven, Department of Neurosciences, Experimental Neurology and Leuven Research Institute for Neuroscience and Disease (LIND), B-3000 Leuven, Belgium; VIB, Center for Brain and Disease Research, Laboratory of Neurobiology, Leuven, Belgium; University Hospitals Leuven, Department of Neurology, Leuven, Belgium.

^121^Sobell Department of Motor Neuroscience and Movement Disorders, University College London, Institute of Neurology, London, UK.

^122^Centro Clinico NeMO, Institute of Neurology, Catholic University, Largo F. Vito 1, 00168 Rome, Italy.

^123^NEuroMuscular Omnicenter (NEMO), Serena Onlus Foundation, Milan, Italy.

^124^Neurology Department, Ulm University, Albert-Einstein-Allee 11, 89081 Ulm, Germany.

^125^Department of Pharmacology and Clinical Neuroscience, Umeå University, Umeå SE-90185, Sweden.

^126^ALS Center, CHU Gui de Chauliac, University of Montpellier, Montpellier, France.

^127^Department of Pathology and Laboratory Medicine, University of Pennsylvania, Philadelphia, PA, USA.

^128^Genetics and Pharmacogenomics, MRL, Merck & Co., Inc., West Point, PA 19486, USA.

^129^"Rita Levi Montalcini" Department of Neuroscience, University of Turin, Turin, Italy; Neuroscience Institute of Torino, Turin 10124, Italy.

^130^Neuromuscular Diseases Research Section, Laboratory of Neurogenetics, National Institute on Aging, NIH, Porter Neuroscience Research Center, Bethesda, MD 20892, USA; Department of Neurology, Johns Hopkins University, Baltimore, MD 21287, USA. Electronic address: bryan.traynor@nih.gov.

^131^Department of Neurology, University of Massachusetts Medical School, Worcester, MA 01605, USA. Electronic address: [john.landers@umassmed.edu](mailto:john.landers@umassmed.edu).

**5. International Multiple Sclerosis Genetics Consortium:**

Nikolaos A Patsopoulos, Sergio E Baranzini, Adam Santaniello, Parisa Shoostari, Chris Cotsapas, Garrett Wong, Ashley H Beecham, Tojo James, Joseph Replogle, Ioannis S Vlachos, Cristin McCabe, Tune H Pers, Aaron Brandes, Charles White, Brendan Keenan, Maria Cimpean, Phoebe Winn, Ioannis-Pavlos Panteliadis, Allison Robbins, Till F M Andlauer, Onigiusz Zarzycki, Bénédicte Dubois, An Goris, Helle Bach Søndergaard, Finn Sellebjerg, Per Soelberg Sorensen, Henrik Ullum, Lise Wegner Thørner, Janna Saarela, Isabelle Cournu-Rebeix, Vincent Damotte, Bertrand Fontaine, Lena Guillot-Noel, Mark Lathrop, Sandra Vukusic, Achim Berthele, Viola Pongratz, Dorothea Buck, Christiane Gasperi, Christiane Graetz, Verena Grummel, Bernhard Hemmer, Muni Hoshi, Benjamin Knier, Thomas Korn, Christina M Lill, Felix Luessi, Mark Mühlau, Frauke Zipp, Efthimios Dardiotis, Cristina Agliardi, Antonio Amoroso, Nadia Barizzone, Maria D Benedetti, Luisa Bernardinelli, Paola Cavalla, Ferdinando Clarelli, Giancarlo Comi, Daniele Cusi, Federica Esposito, Laura Ferrè, Daniela Galimberti, Clara Guaschino, Maurizio A Leone, Vittorio Martinelli, Lucia Moiola, Marco Salvetti, Melissa Sorosina, Domizia Vecchio, Andrea Zauli, Silvia Santoro, Nicasio Mancini, Miriam Zuccalà, Julia Mescheriakova, Cornelia van Duijn, Steffan D Bos, Elisabeth G Celius, Anne Spurkland, Manuel Comabella, Xavier Montalban, Lars Alfredsson, Izaura L Bomfim, David Gomez-Cabrero, Jan Hillert, Maja Jagodic, Magdalena Lindén, Fredrik Piehl, Ilijas Jelčić, Roland Martin, Mirela Sospedra, Amie Baker, Maria Ban, Clive Hawkins, Pirro Hysi, Seema Kalra, Fredrik Karpe, Jyoti Khadake, Genevieve Lachance, Paul Molyneux, Matthew Neville, John Thorpe, Elizabeth Bradshaw, Stacy J Caillier, Peter Calabresi, Bruce A C Cree, Anne Cross, Mary Davis, Paul W I de Bakker, Silvia Delgado, Marieme Dembele, Keith Edwards, Kate Fitzgerald, Irene Y Frohlich, Pierre-Antoine Gourraud, Jonathan L Haines, Hakon Hakonarson, Dorlan Kimbrough, Noriko Isobe, Ioanna Konidari, Ellen Lathi, Michelle H Lee, Taibo Li, David An, Andrew Zimmer, Lohith Madireddy, Clara P Manrique, Mitja Mitrovic, Marta Olah, Ellis Patrick, Margaret A Pericak-Vance, Laura Piccio, Cathy Schaefer, Howard Weiner, Kasper Lage, Alastair Compston, David Hafler, Hanne F Harbo, Stephen L Hauser, Graeme Stewart, Sandra D'Alfonso, Georgios Hadjigeorgiou, Bruce Taylor, Lisa F Barcellos, David Booth, Rogier Hintzen, Ingrid Kockum, Filippo Martinelli-Boneschi, Jacob L McCauley, Jorge R Oksenberg, Annette Oturai, Stephen Sawcer, Adrian J Ivinson, Tomas Olsson, Philip L De Jager

**6. International League Against Epilepsy Consortium on Complex Epilepsies**

Bassel Abou-Khalil, Pauls Auce, Andreja Avbersek, Melanie Bahlo, David J Balding, Thomas Bast, Larry Baum, Albert J Becker, Felicitas Becker, Bianca Berghuis, Samuel F Berkovic, Katja E Boysen, Jonathan P Bradfield, Lawrence C Brody, Russell J Buono, Ellen Campbell, Gregory D Cascino, Claudia B Catarino, Gianpiero L Cavalleri, Stacey S Cherny, Krishna Chinthapalli, Alison J Coffey, Alastair Compston, Antonietta Coppola, Patrick Cossette, John J Craig, Gerrit-Jan de Haan, Peter De Jonghe, Carolien G F de Kovel, Norman Delanty, Chantal Depondt, Orrin Devinsky, Dennis J Dlugos, Colin P Doherty, Christian E Elger, Johan G Eriksson, Thomas N Ferraro, Martha Feucht, Ben Francis, Andre Franke, Jacqueline A French, Saskia Freytag, Verena Gaus, Eric B Geller, Christian Gieger, Tracy Glauser, Simon Glynn, David B Goldstein, Hongsheng Gui, Youling Guo, Kevin F Haas, Hakon Hakonarson, Kerstin Hallmann, Sheryl Haut, Erin L Heinzen, Ingo Helbig, Christian Hengsbach, Helle Hjalgrim, Michele Iacomino, Andrés Ingason, Jennifer Jamnadas-Khoda, Michael R Johnson, Reetta Kälviäinen, Anne-Mari Kantanen, Dalia Kasperavičiūte, Dorothee Kasteleijn-Nolst Trenite, Heidi E Kirsch, Robert C Knowlton, Bobby P C Koeleman, Roland Krause, Martin Krenn, Wolfram S Kunz, Ruben Kuzniecky, Patrick Kwan, Dennis Lal, Yu-Lung Lau, Anna-Elina Lehesjoki, Holger Lerche, Costin Leu, Wolfgang Lieb, Dick Lindhout, Warren D Lo, Iscia Lopes-Cendes, Daniel H Lowenstein, Alberto Malovini, Anthony G Marson, Thomas Mayer, Mark McCormack, James L Mills, Nasir Mirza, Martina Moerzinger, Rikke S Møller, Anne M Molloy, Hiltrud Muhle, Mark Newton, Ping-Wing Ng, Markus M Nöthen, Peter Nürnberg, Terence J O'Brien, Karen L Oliver, Aarno Palotie, Faith Pangilinan, Sarah Peter, Slavé Petrovski, Annapurna Poduri, Michael Privitera, Rodney Radtke, Sarah Rau, Philipp S Reif, Eva M Reinthaler, Felix Rosenow, Josemir W Sander, Thomas Sander, Theresa Scattergood, Steven C Schachter, Christoph J Schankin, Ingrid E Scheffer, Bettina Schmitz, Susanne Schoch, Pak C Sham, Jerry J Shih, Graeme J Sills, Sanjay M Sisodiya, Lisa Slattery, Alexander Smith, David F Smith, Michael C Smith, Philip E Smith, Anja C M Sonsma, Doug Speed, Michael R Sperling, Bernhard J Steinhoff, Ulrich Stephani, Remi Stevelink, Konstantin Strauch, Pasquale Striano, Hans Stroink, Rainer Surges, K Meng Tan, Liu Lin Thio, G Neil Thomas, Marian Todaro, Rossana Tozzi, Maria S Vari, Eileen P G Vining, Frank Visscher, Sarah von Spiczak, Nicole M Walley, Yvonne G Weber, Zhi Wei, Judith Weisenberg, Christopher D Whelan, Peter Widdess-Walsh, Markus Wolff, Stefan Wolking, Wanling Yang, Federico Zara, Fritz Zimprich

## 7. Members of the MEGASTROKE Consortium

Rainer Malik^1^, Ganesh Chauhan^2^, Matthew Traylor^3^, Muralidharan Sargurupremraj^4,5^, Yukinori Okada^6,7,8^, Aniket Mishra^4,5^, Loes Rutten-Jacobs ^3^, Anne-Katrin Giese ^9^, Sander W van der Laan ^10^, Solveig Gretarsdottir ^11^, Christopher D Anderson ^12,13,14,14^, Michael Chong ^15^, Hieab HH Adams ^16,17^, Tetsuro Ago ^18^, Peter Almgren ^19^, Philippe Amouyel ^20,21^, Hakan Ay ^22,13^, Traci M Bartz ^23^, Oscar R Benavente ^24^, Steve Bevan ^25^, Giorgio B Boncoraglio ^26^, Robert D Brown, Jr. ^27^, Adam S Butterworth ^28,29^, Caty Carrera ^30,31^, Cara L Carty ^32,33^, Daniel I Chasman ^34,35^, Wei-Min Chen ^36^, John W Cole ^37^, Adolfo Correa ^38^, Ioana Cotlarciuc ^39^, Carlos Cruchaga ^40,41^, John Danesh ^28,42,43,44^, Paul IW de Bakker ^45,46^, Anita L DeStefano ^47,48^, Marcel den Hoed ^49^, Qing Duan ^50^, Stefan T Engelter ^51,52^, Guido J Falcone ^53,54^, Rebecca F Gottesman ^55^, Raji P Grewal ^56^, Vilmundur Gudnason ^57,58^, Stefan Gustafsson ^59^, Jeffrey Haessler ^60^, Tamara B Harris ^61^, Ahamad Hassan ^62^, Aki S Havulinna ^63,64^, Susan R Heckbert ^65^, Elizabeth G Holliday ^66,67^, George Howard ^68^, Fang-Chi Hsu ^69^, Hyacinth I Hyacinth ^70^, M Arfan Ikram ^16^, Erik Ingelsson ^71,72^, Marguerite R Irvin ^73^, Xueqiu Jian ^74^, Jordi Jiménez-Conde ^75^, Julie A Johnson ^76,77^, J Wouter Jukema ^78^, Masahiro Kanai ^6,7,79^, Keith L Keene ^80,81^, Brett M Kissela ^82^, Dawn O Kleindorfer ^82^, Charles Kooperberg ^60^, Michiaki Kubo ^83^, Leslie A Lange ^84^, Carl D Langefeld ^85^, Claudia Langenberg ^86^, Lenore J Launer ^87^, Jin-Moo Lee ^88^, Robin Lemmens ^89,90^, Didier Leys ^91^, Cathryn M Lewis ^92,93^, Wei-Yu Lin ^28,94^, Arne G Lindgren ^95,96^, Erik Lorentzen ^97^, Patrik K Magnusson ^98^, Jane Maguire ^99^, Ani Manichaikul ^36^, Patrick F McArdle ^100^, James F Meschia ^101^, Braxton D Mitchell ^100,102^, Thomas H Mosley ^103,104^, Michael A Nalls ^105,106^, Toshiharu Ninomiya ^107^, Martin J O'Donnell ^15,108^, Bruce M Psaty ^109,110,111,112^, Sara L Pulit ^113,45^, Kristiina Rannikmäe ^114,115^, Alexander P Reiner ^65,116^, Kathryn M Rexrode ^117^, Kenneth Rice ^118^, Stephen S Rich ^36^, Paul M Ridker ^34,35^, Natalia S Rost ^9,13^, Peter M Rothwell ^119^, Jerome I Rotter ^120,121^, Tatjana Rundek ^122^, Ralph L Sacco ^122^, Saori Sakaue ^7,123^, Michele M Sale ^124^, Veikko Salomaa ^63^, Bishwa R Sapkota ^125^, Reinhold Schmidt ^126^, Carsten O Schmidt ^127^, Ulf Schminke ^128^, Pankaj Sharma ^39^, Agnieszka Slowik ^129^, Cathie LM Sudlow ^114,115^, Christian Tanislav ^130^, Turgut Tatlisumak ^131,132^, Kent D Taylor ^120,121^, Vincent NS Thijs ^133,134^, Gudmar Thorleifsson ^11^, Unnur Thorsteinsdottir ^11^, Steffen Tiedt ^1^, Stella Trompet ^135^, Christophe Tzourio ^5,136,137^, Cornelia M van Duijn ^138,139^, Matthew Walters ^140^, Nicholas J Wareham ^86^, Sylvia Wassertheil-Smoller ^141^, James G Wilson ^142^, Kerri L Wiggins ^109^, Qiong Yang ^47^, Salim Yusuf ^15^, Najaf Amin ^16^, Hugo S Aparicio ^185,48^, Donna K Arnett ^186^, John Attia ^187^, Alexa S Beiser ^47,48^, Claudine Berr ^188^, Julie E Buring ^34,35^, Mariana Bustamante ^189^, Valeria Caso ^190^, Yu-Ching Cheng ^191^, Seung Hoan Choi ^192,48^, Ayesha Chowhan ^185,48^, Natalia Cullell ^31^, Jean-François Dartigues ^193,194^, Hossein Delavaran ^95,96^, Pilar Delgado ^195^, Marcus Dörr ^196,197^, Gunnar Engström ^19^, Ian Ford ^198^, Wander S Gurpreet ^199^, Anders Hamsten ^200,201^, Laura Heitsch ^202^, Atsushi Hozawa ^203^, Laura Ibanez ^204^, Andreea Ilinca ^95,96^, Martin Ingelsson ^205^, Motoki Iwasaki ^206^, Rebecca D Jackson ^207^, Katarina Jood ^208^, Pekka Jousilahti ^63^, Sara Kaffashian ^4,5^, Lalit Kalra ^209^, Masahiro Kamouchi ^210^, Takanari Kitazono ^211^, Olafur Kjartansson ^212^, Manja Kloss ^213^, Peter J Koudstaal ^214^, Jerzy Krupinski ^215^, Daniel L Labovitz ^216^, Cathy C Laurie ^118^, Christopher R Levi ^217^, Linxin Li ^218^, Lars Lind ^219^, Cecilia M Lindgren ^220,221^, Vasileios Lioutas ^222,48^, Yong Mei Liu ^223^, Oscar L Lopez ^224^, Hirata Makoto ^225^, Nicolas Martinez-Majander ^172^, Koichi Matsuda ^225^, Naoko Minegishi ^203^, Joan Montaner ^226^, Andrew P Morris ^227,228^, Elena Muiño ^31^, Martina Müller-Nurasyid ^229,230,231^, Bo Norrving ^95,96^, Soichi Ogishima ^203^, Eugenio A Parati ^232^, Leema Reddy Peddareddygari ^56^, Nancy L Pedersen ^98,233^, Joanna Pera ^129^, Markus Perola ^63,234^, Alessandro Pezzini ^235^, Silvana Pileggi ^236^, Raquel Rabionet ^237^, Iolanda Riba-Llena ^30^, Marta Ribasés ^238^, Jose R Romero ^185,48^, Jaume Roquer ^239,240^, Anthony G Rudd ^241,242^, Antti-Pekka Sarin ^243,244^, Ralhan Sarju ^199^, Chloe Sarnowski ^47,48^, Makoto Sasaki ^245^, Claudia L Satizabal ^185,48^, Mamoru Satoh ^245^, Naveed Sattar ^246^, Norie Sawada ^206^, Gerli Sibolt ^172^, Ásgeir Sigurdsson ^247^, Albert Smith ^248^, Kenji Sobue ^245^, Carolina Soriano-Tárraga ^240^, Tara Stanne ^249^, O Colin Stine ^250^, David J Stott ^251^, Konstantin Strauch ^229,252^, Takako Takai ^203^, Hideo Tanaka ^253,254^, Kozo Tanno ^245^, Alexander Teumer ^255^, Liisa Tomppo ^172^, Nuria P Torres-Aguila ^31^, Emmanuel Touze ^256,257^, Shoichiro Tsugane ^206^, Andre G Uitterlinden ^258^, Einar M Valdimarsson ^259^, Sven J van der Lee ^16^, Henry Völzke ^255^, Kenji Wakai ^253^, David Weir ^260^, Stephen R Williams ^261^, Charles DA Wolfe ^241,242^, Quenna Wong ^118^, Huichun Xu ^191^, Taiki Yamaji ^206^, Dharambir K Sanghera ^125,169,170^, Olle Melander ^19^, Christina Jern ^171^, Daniel Strbian ^172,173^, Israel Fernandez-Cadenas ^31,30^, W T Longstreth, Jr ^174,65^, Arndt Rolfs ^175^, Jun Hata ^107^, Daniel Woo ^82^, Jonathan Rosand ^12,13,14^, Guillaume Pare ^15^, Jemma C Hopewell ^176^, Danish Saleheen ^177^, Kari Stefansson ^11,178^, Bradford B Worrall ^179^, Steven J Kittner ^37^, Sudha Seshadri ^180,48^, Myriam Fornage ^74,181^, Hugh S Markus ^3^, Joanna MM Howson ^28^, Yoichiro Kamatani ^6,182^, Stephanie Debette ^4,5^, Martin Dichgans ^1,183,184^

^1^ Institute for Stroke and Dementia Research (ISD), University Hospital, LMU Munich, Munich, Germany

^2^ Centre for Brain Research, Indian Institute of Science, Bangalore, India

^3^ Stroke Research Group, Division of Clinical Neurosciences, University of Cambridge, UK

^4^ INSERM U1219 Bordeaux Population Health Research Center, Bordeaux, France

^5^ University of Bordeaux, Bordeaux, France

^6^ Laboratory for Statistical Analysis, RIKEN Center for Integrative Medical Sciences, Yokohama, Japan

^7^ Department of Statistical Genetics, Osaka University Graduate School of Medicine, Osaka, Japan

^8^Laboratory of Statistical Immunology, Immunology Frontier Research Center (WPI-IFReC), Osaka University, Suita, Japan

^9^ Department of Neurology, Massachusetts General Hospital, Harvard Medical School, Boston, MA, USA

^10^ Laboratory of Experimental Cardiology, Division of Heart and Lungs, University Medical Center Utrecht, University of Utrecht, Utrecht,Netherlands

^11^ deCODE genetics/AMGEN inc, Reykjavik, Iceland

^12^ Center for Genomic Medicine, Massachusetts General Hospital (MGH), Boston, MA, USA

^13^ J. Philip Kistler Stroke Research Center, Department of Neurology, MGH, Boston, MA, USA

^14^Program in Medical and Population Genetics, Broad Institute, Cambridge, MA, USA

^15^ Population Health Research Institute, McMaster University, Hamilton, Canada

^16^ Department of Epidemiology, Erasmus University Medical Center, Rotterdam, Netherlands

^17^ Department of Radiology and Nuclear Medicine, Erasmus University Medical Center, Rotterdam, Netherlands

^18^ Department of Medicine and Clinical Science, Graduate School of Medical Sciences, Kyushu University, Fukuoka, Japan

^19^ Department of Clinical Sciences, Lund University, Malmö, Sweden

^20^ Univ. Lille, Inserm, Institut Pasteur de Lille, LabEx DISTALZ-UMR1167, Risk factors and molecular determinants of aging-related diseases, F-59000 Lille, France

^21^ Centre Hosp. Univ Lille, Epidemiology and Public Health Department, F-59000 Lille, France

^22^ AA Martinos Center for Biomedical Imaging, Department of Radiology, Massachusetts General Hospital, Harvard Medical School, Boston, MA, USA

^23^ Cardiovascular Health Research Unit, Departments of Biostatistics and Medicine, University of Washington, Seattle, WA, USA

^24^ Division of Neurology, Faculty of Medicine, Brain Research Center, University of British Columbia, Vancouver, Canada

^25^ School of Life Science, University of Lincoln, Lincoln, UK

^26^ Department of Cerebrovascular Diseases, Fondazione IRCCS Istituto Neurologico "Carlo Besta", Milano, Italy

^27^ Department of Neurology, Mayo Clinic Rochester, Rochester, MN, USA

^28^ MRC/BHF Cardiovascular Epidemiology Unit, Department of Public Health and Primary Care, University of Cambridge, Cambridge, UK

^29^ The National Institute for Health Research Blood and Transplant Research Unit in Donor Health and Genomics, University of Cambridge, UK

^30^ Neurovascular Research Laboratory, Vall d'Hebron Institut of Research, Neurology and Medicine Departments-Universitat Autònoma de Barcelona, Vall d’Hebrón Hospital, Barcelona, Spain

^31^ Stroke Pharmacogenomics and Genetics, Fundacio Docència i Recerca MutuaTerrassa, Terrassa, Spain

^32^ Children's Research Institute, Children's National Medical Center, Washington, DC, USA

^33^Center for Translational Science, George Washington University, Washington, DC, USA

^34^Division of Preventive Medicine, Brigham and Women's Hospital, Boston, MA, USA

^35^ Harvard Medical School, Boston, MA, USA

^36^ Center for Public Health Genomics, Department of Public Health Sciences, University of Virginia, Charlottesville, VA, USA

^37^ Department of Neurology, University of Maryland School of Medicine and Baltimore VAMC, Baltimore, MD, USA

^38^ Departments of Medicine, Pediatrics and Population Health Science, University of Mississippi Medical Center, Jackson, MS, USA

^39^ Institute of Cardiovascular Research, Royal Holloway University of London, UK & Ashford and St Peters Hospital, Surrey UK

^40^ Department of Psychiatry,The Hope Center Program on Protein Aggregation and Neurodegeneration (HPAN),Washington University, School of Medicine, St. Louis, MO, USA

^41^ Department of Developmental Biology, Washington University School of Medicine, St. Louis, MO, USA

^42^ NIHR Blood and Transplant Research Unit in Donor Health and Genomics, Department of Public Health and Primary Care, University of Cambridge, Cambridge, UK

^43^ Wellcome Trust Sanger Institute, Wellcome Trust Genome Campus, Hinxton, Cambridge, UK

^44^ British Heart Foundation, Cambridge Centre of Excellence, Department of Medicine, University of Cambridge, Cambridge, UK

^45^ Department of Medical Genetics, University Medical Center Utrecht, Utrecht, Netherlands

^46^ Department of Epidemiology, Julius Center for Health Sciences and Primary Care, University Medical Center Utrecht, Utrecht, Netherlands

^47^ Boston University School of Public Health, Boston, MA, USA

^48^ Framingham Heart Study, Framingham, MA, USA

^49^ Department of Immunology, Genetics and Pathology and Science for Life Laboratory, Uppsala University, Uppsala, Sweden

^50^Department of Genetics, University of North Carolina, Chapel Hill, NC, USA

^51^ Department of Neurology and Stroke Center, Basel University Hospital, Switzerland

^52^ Neurorehabilitation Unit, University and University Center for Medicine of Aging and Rehabilitation Basel, Felix Platter Hospital, Basel, Switzerland

^53^ Department of Neurology, Yale University School of Medicine, New Haven, CT, USA

^54^ Program in Medical and Population Genetics, The Broad Institute of Harvard and MIT, Cambridge, MA, USA

^55^ Department of Neurology, Johns Hopkins University School of Medicine, Baltimore, MD, USA

^56^ Neuroscience Institute, SF Medical Center, Trenton, NJ, USA

^57^ Icelandic Heart Association Research Institute, Kopavogur, Iceland

^58^ University of Iceland, Faculty of Medicine, Reykjavik, Iceland

^59^ Department of Medical Sciences, Molecular Epidemiology and Science for Life Laboratory, Uppsala University, Uppsala, Sweden

^60^ Division of Public Health Sciences, Fred Hutchinson Cancer Research Center, Seattle, WA, USA

^61^ Laboratory of Epidemiology and Population Science, National Institute on Aging, National Institutes of Health, Bethesda, MD, USA

^62^ Department of Neurology, Leeds General Infirmary, Leeds Teaching Hospitals NHS Trust, Leeds, UK

^63^ National Institute for Health and Welfare, Helsinki, Finland

^64^ FIMM - Institute for Molecular Medicine Finland, Helsinki, Finland

^65^Department of Epidemiology, University of Washington, Seattle, WA, USA

^66^ Public Health Stream, Hunter Medical Research Institute, New Lambton, Australia

^67^Faculty of Health and Medicine, University of Newcastle, Newcastle, Australia

^68^ School of Public Health, University of Alabama at Birmingham, Birmingham, AL, USA

^69^Department of Biostatistical Sciences, Wake Forest School of Medicine, Winston-Salem, NC, USA

^70^ Aflac Cancer and Blood Disorder Center, Department of Pediatrics, Emory University School of Medicine, Atlanta, GA, USA

^71^ Department of Medicine, Division of Cardiovascular Medicine, Stanford University School of Medicine, CA, USA

^72^ Department of Medical Sciences, Molecular Epidemiology and Science for Life Laboratory, Uppsala University, Uppsala, Sweden

^73^ Epidemiology, School of Public Health, University of Alabama at Birmingham, USA

^74^ Brown Foundation Institute of Molecular Medicine, University of Texas Health Science Center at Houston, Houston, TX, USA

^75^ Neurovascular Research Group (NEUVAS), Neurology Department, Institut Hospital del Mar d'Investigació Mèdica, Universitat Autònoma de Barcelona, Barcelona, Spain

^76^ Department of Pharmacotherapy and Translational Research and Center for Pharmacogenomics, University of Florida, College of Pharmacy, Gainesville, FL, USA

^77^ Division of Cardiovascular Medicine, College of Medicine, University of Florida, Gainesville, FL, USA

^78^ Department of Cardiology, Leiden University Medical Center, Leiden, the Netherlands

^79^Program in Bioinformatics and Integrative Genomics, Harvard Medical School, Boston, MA, USA

^80^ Department of Biology, East Carolina University, Greenville, NC, USA

^81^ Center for Health Disparities, East Carolina University, Greenville, NC, USA

^82^ University of Cincinnati College of Medicine, Cincinnati, OH, USA

^83^ RIKEN Center for Integrative Medical Sciences, Yokohama, Japan

^84^ Department of Medicine, University of Colorado Denver, Anschutz Medical Campus, Aurora, CO, USA

^85^Center for Public Health Genomics and Department of Biostatistical Sciences, Wake Forest School of Medicine, Winston-Salem, NC, USA

^86^ MRC Epidemiology Unit, University of Cambridge School of Clinical Medicine, Institute of Metabolic Science, Cambridge Biomedical Campus, Cambridge, UK

^87^ Intramural Research Program, National Institute on Aging, National Institutes of Health, Bethesda, MD, USA

^88^ Department of Neurology, Radiology, and Biomedical Engineering, Washington University School of Medicine, St. Louis, MO, USA

^89^ KU Leuven – University of Leuven, Department of Neurosciences, Experimental Neurology, Leuven, Belgium

^90^ VIB Center for Brain & Disease Research, University Hospitals Leuven, Department of Neurology, Leuven, Belgium

^91^ Univ.-Lille, INSERM U 1171. CHU Lille. Lille, France

^92^ Department of Medical and Molecular Genetics, King's College London, London, UK

^93^ SGDP Centre, Institute of Psychiatry, Psychology & Neuroscience, King's College London, London, UK

^94^ Northern Institute for Cancer Research, Paul O'Gorman Building, Newcastle University, Newcastle, UK

^95^ Department of Clinical Sciences Lund, Neurology, Lund University, Lund, Sweden

^96^ Department of Neurology and Rehabilitation Medicine, Skåne University Hospital, Lund, Sweden

^97^ Bioinformatics Core Facility, University of Gothenburg, Gothenburg, Sweden

^98^ Department of Medical Epidemiology and Biostatistics, Karolinska Institutet, Stockholm, Sweden

^99^ University of Technology Sydney, Faculty of Health, Ultimo, Australia

^100^ Department of Medicine, University of Maryland School of Medicine, MD, USA

^101^ Department of Neurology, Mayo Clinic, Jacksonville, FL, USA

^102^ Geriatrics Research and Education Clinical Center, Baltimore Veterans Administration Medical Center, Baltimore, MD, USA

^103^ Division of Geriatrics, School of Medicine, University of Mississippi Medical Center, Jackson, MS, USA

^104^ Memory Impairment and Neurodegenerative Dementia Center, University of Mississippi Medical Center, Jackson, MS, USA

^105^ Laboratory of Neurogenetics, National Institute on Aging, National institutes of Health, Bethesda, MD, USA

^106^ Data Tecnica International, Glen Echo MD, USA

^107^ Department of Epidemiology and Public Health, Graduate School of Medical Sciences, Kyushu University, Fukuoka, Japan

^108^ Clinical Research Facility, Department of Medicine, NUI Galway, Galway, Ireland

^109^ Cardiovascular Health Research Unit, Department of Medicine, University of Washington, Seattle, WA, USA

^110^ Department of Epidemiology, University of Washington, Seattle, WA

^111^ Department of Health Services, University of Washington, Seattle, WA, USA

^112^ Kaiser Permanente Washington Health Research Institute, Seattle, WA, USA

^113^ Brain Center Rudolf Magnus, Department of Neurology, University Medical Center Utrecht, Utrecht, The Netherlands

^114^ Usher Institute of Population Health Sciences and Informatics, University of Edinburgh, Edinburgh, UK

^115^ Centre for Clinical Brain Sciences, University of Edinburgh, Edinburgh, UK

^116^ Fred Hutchinson Cancer Research Center, University of Washington, Seattle, WA, USA

^117^Department of Medicine, Brigham and Women's Hospital, Boston, MA, USA

^118^Department of Biostatistics, University of Washington, Seattle, WA, USA

^119^ Nuffield Department of Clinical Neurosciences, University of Oxford, UK

^120^ Institute for Translational Genomics and Population Sciences, Los Angeles Biomedical Research Institute at Harbor-UCLA Medical Center, Torrance, CA, USA

^121^ Division of Genomic Outcomes, Department of Pediatrics, Harbor-UCLA Medical Center, Torrance, CA, USA

^122^ Department of Neurology, Miller School of Medicine, University of Miami, Miami, FL, USA

^123^Department of Allergy and Rheumatology, Graduate School of Medicine, the University of Tokyo, Tokyo, Japan

^124^Center for Public Health Genomics, University of Virginia, Charlottesville, VA, USA

^125^ Department of Pediatrics, College of Medicine, University of Oklahoma Health Sciences Center, Oklahoma City, OK, USA

^126^Department of Neurology, Medical University of Graz, Graz, Austria

^127^ University Medicine Greifswald, Institute for Community Medicine, SHIP-KEF, Greifswald, Germany

^128^ University Medicine Greifswald, Department of Neurology, Greifswald, Germany

^129^ Department of Neurology, Jagiellonian University, Krakow, Poland

^130^ Department of Neurology, Justus Liebig University, Giessen, Germany

^131^ Department of Clinical Neurosciences/Neurology, Institute of Neuroscience and Physiology, Sahlgrenska Academy at University of Gothenburg, Gothenburg, Sweden

^132^ Sahlgrenska University Hospital, Gothenburg, Sweden

^133^ Stroke Division, Florey Institute of Neuroscience and Mental Health, University of Melbourne, Heidelberg, Australia

^134^ Austin Health, Department of Neurology, Heidelberg, Australia

^135^ Department of Internal Medicine, Section Gerontology and Geriatrics, Leiden University Medical Center, Leiden, the Netherlands

^136^ INSERM U1219, Bordeaux, France

^137^Department of Public Health, Bordeaux University Hospital, Bordeaux, France

^138^ Genetic Epidemiology Unit, Department of Epidemiology, Erasmus University Medical Center Rotterdam, Netherlands

^139^ Center for Medical Systems Biology, Leiden, Netherlands

^140^School of Medicine, Dentistry and Nursing at the University of Glasgow, Glasgow, UK

^141^Department of Epidemiology and Population Health, Albert Einstein College of Medicine, NY, USA

^142^Department of Physiology and Biophysics, University of Mississippi Medical Center, Jackson, MS, USA

^143^ A full list of members and affiliations appears in the Supplementary Note

^144^Department of Human Genetics, McGill University, Montreal, Canada

^145^Department of Pathophysiology, Institute of Biomedicine and Translation Medicine, University of Tartu, Tartu, Estonia

^146^ Department of Cardiac Surgery, Tartu University Hospital, Tartu, Estonia

^147^ Clinical Gene Networks AB,Stockholm, Sweden

^148^ Department of Genetics and Genomic Sciences, The Icahn Institute for Genomics and Multiscale Biology Icahn School of Medicine at Mount Sinai, New York, NY, USA

^149^Department of Pathophysiology, Institute of Biomedicine and Translation Medicine, University of Tartu, Biomeedikum, Tartu, Estonia

^150^ Integrated Cardio Metabolic Centre, Department of Medicine, Karolinska Institutet, Karolinska Universitetssjukhuset, Huddinge, Sweden

^151^ Clinical Gene Networks AB, Stockholm, Sweden

^152^ Sorbonne Universités, UPMC Univ. Paris 06, INSERM, UMR_S 1166, Team Genomics & Pathophysiology of Cardiovascular Diseases, Paris, France

^153^ ICAN Institute for Cardiometabolism and Nutrition, Paris, France

^154^Department of Biomedical Engineering, University of Virginia, Charlottesville, VA, USA

^155^ Group Health Research Institute, Group Health Cooperative, Seattle, WA, USA

^156^ Seattle Epidemiologic Research and Information Center, VA Office of Research and Development, Seattle, WA, USA

^157^ Cardiovascular Research Center, Massachusetts General Hospital, Boston, MA, USA

^158^Department of Medical Research, Bærum Hospital, Vestre Viken Hospital Trust, Gjettum, Norway

^159^ Saw Swee Hock School of Public Health, National University of Singapore and National University Health System, Singapore

^160^ National Heart and Lung Institute, Imperial College London, London, UK

^161^ Department of Gene Diagnostics and Therapeutics, Research Institute, National Center for Global Health and Medicine, Tokyo, Japan

^162^ Department of Epidemiology, Tulane University School of Public Health and Tropical Medicine, New Orleans, LA, USA

^163^ Department of Cardiology,University Medical Center Groningen, University of Groningen, Netherlands

^164^ MRC-PHE Centre for Environment and Health, School of Public Health, Department of Epidemiology and Biostatistics, Imperial College London, London, UK

^165^Department of Epidemiology and Biostatistics, Imperial College London, London, UK

^166^Department of Cardiology, Ealing Hospital NHS Trust, Southall, UK

^167^ National Heart, Lung and Blood Research Institute, Division of Intramural Research, Population Sciences Branch, Framingham, MA, USA

^168^ A full list of members and affiliations appears at the end of the manuscript

^169^ Department of Phamaceutical Sciences, Collge of Pharmacy, University of Oklahoma Health Sciences Center, Oklahoma City, OK, USA

^170^ Oklahoma Center for Neuroscience, Oklahoma City, OK, USA

^171^ Department of Pathology and Genetics, Institute of Biomedicine, The Sahlgrenska Academy at University of Gothenburg, Gothenburg, Sweden

^172^Department of Neurology, Helsinki University Hospital, Helsinki, Finland

^173^ Clinical Neurosciences, Neurology, University of Helsinki, Helsinki, Finland

^174^ Department of Neurology, University of Washington, Seattle, WA, USA

^175^ Albrecht Kossel Institute, University Clinic of Rostock, Rostock, Germany

^176^ Clinical Trial Service Unit and Epidemiological Studies Unit, Nuffield Department of Population Health, University of Oxford, Oxford, UK

^177^ Department of Genetics, Perelman School of Medicine, University of Pennsylvania, PA, USA

^178^ Faculty of Medicine, University of Iceland, Reykjavik, Iceland

^179^ Departments of Neurology and Public Health Sciences, University of Virginia School of Medicine, Charlottesville, VA, USA

^180^ Department of Neurology, Boston University School of Medicine, Boston, MA, USA

^181^ Human Genetics Center, University of Texas Health Science Center at Houston, Houston, TX, USA

^182^Center for Genomic Medicine, Kyoto University Graduate School of Medicine, Kyoto, Japan

^183^ Munich Cluster for Systems Neurology (SyNergy), Munich, Germany

^184^ German Center for Neurodegenerative Diseases (DZNE), Munich, Germany

^185^ Boston University School of Medicine, Boston, MA, USA

^186^ University of Kentucky College of Public Health, Lexington, KY, USA

^187^University of Newcastle and Hunter Medical Research Institute, New Lambton, Australia

^188^ Univ. Montpellier, Inserm, U1061, Montpellier, France

^189^Centre for Research in Environmental Epidemiology, Barcelona, Spain

^190^ Department of Neurology, Università degli Studi di Perugia, Umbria, Italy

^191^Department of Medicine, University of Maryland School of Medicine, Baltimore, MD, USA

^192^ Broad Institute, Cambridge, MA, USA

^193^ Univ. Bordeaux, Inserm, Bordeaux Population Health Research Center, UMR 1219, Bordeaux, France

^194^ Bordeaux University Hospital, Department of Neurology, Memory Clinic, Bordeaux, France

^195^ Neurovascular Research Laboratory. Vall d'Hebron Institut of Research, Neurology and Medicine Departments-Universitat Autònoma de Barcelona. Vall d’Hebrón Hospital, Barcelona, Spain

^196^ University Medicine Greifswald, Department of Internal Medicine B, Greifswald, Germany

^197^ DZHK, Greifswald, Germany

^198^ Robertson Center for Biostatistics, University of Glasgow, Glasgow, UK

^199^ Hero DMC Heart Institute, Dayanand Medical College & Hospital, Ludhiana, India

^200^ Atherosclerosis Research Unit, Department of Medicine Solna, Karolinska Institutet, Stockholm, Sweden

^201^ Karolinska Institutet, Stockholm, Sweden

^202^ Division of Emergency Medicine, and Department of Neurology, Washington University School of Medicine, St. Louis, MO, USA

^203^ Tohoku Medical Megabank Organization, Sendai, Japan

^204^ Department of Psychiatry, Washington University School of Medicine, St. Louis, MO, USA

^205^ Department of Public Health and Caring Sciences / Geriatrics, Uppsala University, Uppsala, Sweden

^206^ Epidemiology and Prevention Group, Center for Public Health Sciences, National Cancer Center, Tokyo, Japan

^207^ Department of Internal Medicine and the Center for Clinical and Translational Science, The Ohio State University, Columbus, OH, USA

^208^ Institute of Neuroscience and Physiology, the Sahlgrenska Academy at University of Gothenburg, Goteborg, Sweden

^209^Department of Basic and Clinical Neurosciences, King's College London, London, UK

^210^ Department of Health Care Administration and Management, Graduate School of Medical Sciences, Kyushu University, Japan

^211^Department of Medicine and Clinical Science, Graduate School of Medical Sciences, Kyushu University, Japan

^212^ Landspitali National University Hospital, Departments of Neurology & Radiology, Reykjavik, Iceland

^213^ Department of Neurology, Heidelberg University Hospital, Germany

^214^ Department of Neurology, Erasmus University Medical Center

^215^ Hospital Universitari Mutua Terrassa, Terrassa (Barcelona), Spain

^216^ Albert Einstein College of Medicine, Montefiore Medical Center, New York, NY, USA

^217^ John Hunter Hospital, Hunter Medical Research Institute and University of Newcastle, Newcastle, NSW, Australia

^218^ Centre for Prevention of Stroke and Dementia, Nuffield Department of Clinical Neurosciences, University of Oxford, UK

^219^ Department of Medical Sciences, Uppsala University, Uppsala, Sweden

^220^ Genetic and Genomic Epidemiology Unit, Wellcome Trust Centre for Human Genetics, University of Oxford, Oxford, UK

^221^ The Wellcome Trust Centre for Human Genetics, Oxford, UK

^222^ Beth Israel Deaconess Medical Center, Boston, MA, USA

^223^ Wake Forest School of Medicine, Wake Forest, NC, USA

^224^ Department of Neurology, University of Pittsburgh, Pittsburgh, PA, USA

^225^ BioBank Japan, Laboratory of Clinical Sequencing, Department of Computational biology and medical Sciences, Graduate school of Frontier Sciences, The University of Tokyo, Tokyo, Japan

^226^ Neurovascular Research Laboratory, Vall d'Hebron Institut of Research, Neurology and Medicine Departments-Universitat Autònoma de Barcelona. Vall d’Hebrón Hospital, Barcelona, Spain

^227^Department of Biostatistics, University of Liverpool, Liverpool, UK

^228^ Wellcome Trust Centre for Human Genetics, University of Oxford, Oxford, UK

^229^ Institute of Genetic Epidemiology, Helmholtz Zentrum München - German Research Center for Environmental Health, Neuherberg, Germany

^230^Department of Medicine I, Ludwig-Maximilians-Universität, Munich, Germany

^231^ DZHK (German Centre for Cardiovascular Research), partner site Munich Heart Alliance, Munich, Germany

^232^ Department of Cerebrovascular Diseases, Fondazione IRCCS Istituto Neurologico “Carlo Besta”, Milano, Italy

^233^ Karolinska Institutet, MEB, Stockholm, Sweden

^234^University of Tartu, Estonian Genome Center, Tartu, Estonia, Tartu, Estonia

^235^Department of Clinical and Experimental Sciences, Neurology Clinic, University of Brescia, Italy

^236^ Translational Genomics Unit, Department of Oncology, IRCCS Istituto di Ricerche Farmacologiche Mario Negri, Milano, Italy

^237^Department of Genetics, Microbiology and Statistics, University of Barcelona, Barcelona, Spain

^238^ Psychiatric Genetics Unit, Group of Psychiatry, Mental Health and Addictions, Vall d’Hebron Research Institute (VHIR), Universitat Autònoma de Barcelona, Biomedical Network Research Centre on Mental Health (CIBERSAM), Barcelona, Spain

^239^ Department of Neurology, IMIM-Hospital del Mar, and Universitat Autònoma de Barcelona, Spain

^240^ IMIM (Hospital del Mar Medical Research Institute), Barcelona, Spain

^241^ National Institute for Health Research Comprehensive Biomedical Research Centre, Guy's & St. Thomas' NHS Foundation Trust and King's College London, London, UK

^242^ Division of Health and Social Care Research, King's College London, London, UK

^243^ FIMM-Institute for Molecular Medicine Finland, Helsinki, Finland

^244^ THL-National Institute for Health and Welfare, Helsinki, Finland

^245^ Iwate Tohoku Medical Megabank Organization, Iwate Medical University, Iwate, Japan

^246^ BHF Glasgow Cardiovascular Research Centre, Faculty of Medicine, Glasgow, UK

^247^ deCODE Genetics/Amgen, Inc., Reykjavik, Iceland

^248^ Icelandic Heart Association, Reykjavik, Iceland

^249^ Institute of Biomedicine, the Sahlgrenska Academy at University of Gothenburg, Goteborg, Sweden

^250^Department of Epidemiology, University of Maryland School of Medicine, Baltimore, MD, USA

^251^ Institute of Cardiovascular and Medical Sciences, Faculty of Medicine, University of Glasgow, Glasgow, UK

^252^Chair of Genetic Epidemiology, IBE, Faculty of Medicine, LMU Munich, Germany

^253^Division of Epidemiology and Prevention, Aichi Cancer Center Research Institute, Nagoya, Japan

^254^ Department of Epidemiology, Nagoya University Graduate School of Medicine, Nagoya, Japan

^255^ University Medicine Greifswald, Institute for Community Medicine, SHIP-KEF, Greifswald, Germany

^256^Department of Neurology, Caen University Hospital, Caen, France

^257^ University of Caen Normandy, Caen, France

^258^Department of Internal Medicine, Erasmus University Medical Center, Rotterdam, Netherlands

^259^ Landspitali University Hospital, Reykjavik, Iceland

^260^ Survey Research Center, University of Michigan, Ann Arbor, MI, USA

^261^ University of Virginia Department of Neurology, Charlottesville, VA, USA
